# Analysis of 26 Studies of the Impact of Coconut Oil on Lipid Parameters: Beyond Total and LDL Cholesterol

**DOI:** 10.3390/nu17030514

**Published:** 2025-01-30

**Authors:** Mary T. Newport, Fabian M. Dayrit

**Affiliations:** 1 Independent Researcher, Spring Hill, FL 34610, USA; 2 Department of Chemistry, Ateneo de Manila University, Loyola Heights, Quezon City 1108, Philippines; fdayrit@ateneo.edu

**Keywords:** coconut oil, saturated fat, dietary fat, medium-chain fatty acids, lipid parameters, lipid ratios, cardiovascular health, dietary guidelines, *trans*-fat, Friedewald formula

## Abstract

Coconut oil (CNO) is often characterized as an “artery-clogging fat” because it is a predominantly saturated fat that ostensibly raises total cholesterol (TChol) and LDL cholesterol (LDL-C). Whereas previous analyses assessed CNO based on the relative effects on lipid parameters against other fats and oils, this analysis focuses on the effects of CNO itself. Here, we review the literature on CNO and analyze 984 lipid profile data sets from 26 CNO studies conducted over the past 40 years. This analysis shows considerable heterogeneity among CNO studies regarding participant selection, the amount consumed, and the study duration. The analysis reveals that, overall, CNO consumption gives variable TChol and LDL-C values, but that the HDL-cholesterol (HDL-C) values increase and triglycerides (TG) decrease. This holistic lipid assessment, together with the consideration of lipid ratios, shows that CNO does not pose a health risk for heart disease. Because the predominantly medium-chain fatty acid profile of CNO is significantly different from that of lard and palm oil, studies using these as reference materials do not apply to CNO. This paper concludes that the recommendation to avoid consuming coconut oil due to the risk of heart disease is not justified.

## 1. Introduction

The steady rise in coronary artery disease (CAD) deaths in the United States between 1900 and 1980 was blamed on saturated fat. Dietary fat guidelines put forth by the American Heart Association (AHA) since 1961 [1] and the US Department of Agriculture (USDA) and Department of Health and Human Services (HHS) in the *Dietary Guidelines for Americans* since 1980 [2] have recommended avoiding the consumption of foods high in saturated fat, including coconut oil (CNO). Given that CNO accounted for less than 1 percent of edible oil consumption in the United States throughout the twentieth century [3], CNO consumption cannot explain this steady rise in CAD. Neither was CNO included in any of the large long-term interventional dietary fat studies conducted in the US [4]. Nevertheless, CNO was characterized as an “artery-clogging fat” for decades, without evidence to justify this claim. The principal objections against CNO are that it is a saturated fat and that it raises the levels of total cholesterol (TChol) and LDL cholesterol (LDL-C). However, these objections ignore the differences between medium-chain fatty acids (MCFAs), which are predominant in CNO, and saturated long-chain fatty acids (LCFAs), which characterize the other members of the so-called group of saturated fats. Although the average TChol and LDL-C levels tended to be lower when saturated fat was replaced with polyunsaturated fat, long-term studies failed to demonstrate that higher intake of saturated fat was linked to a higher incidence of death from CAD and the overall incidence of cardiovascular disease (CVD). Likewise, population and prospective studies of people consuming CNO versus another oil, to be discussed below, have found no difference in adverse cardiovascular events or mortality.

The principal objective of this paper is to review the literature to show whether CNO presents a risk of heart disease. It is not the objective of this paper to compare CNO with other oils and fats with respect to their effects on lipid parameters and related health impacts. This paper is divided into the following sections: (1) a review of the major issues regarding CNO and saturated fat; (2) an analysis of the data from 26 CNO studies; (3) the results of the analysis; (4) a discussion; and (5) summary and conclusions. 

## 2. Review of the Major Issues Regarding Coconut Oil and Saturated Fat

This section will discuss the basic suppositions that underlie our understanding of saturated fat in general and CNO in particular and its effects on serum lipids. This section will also discuss medium-chain fatty acids and *trans*-fats and review relevant dietary and clinical studies that have been conducted on CNO and polyunsaturated fat.

### 2.1. “Saturated Fats” Are a Heterogenous Group

It is often assumed that “saturated fats” are all the same. Historically, in commerce before the 20th century, fats and oils were classified primarily based on their physical properties, such as being solid or liquid at room temperature, which was assumed to be 25 °C (77 °F). The commercial classification of solid fat with saturated fat arose from the mistaken assumption that fats and oils that are solid at room temperature are saturated. Unfortunately, this erroneous definition of solid fat with saturated fat has not been corrected, even with the availability of chemical analytical instrumentation—in particular, gas chromatography, which was already being used for the analysis of fatty acids in 1952 [5].

However, the group of “saturated fats” as currently defined is quite heterogeneous. More precisely, saturated fats can be divided into four groups: plant oils that are predominantly medium-chain fatty acids (MCFAs, C6:0 to C12:0); plant oils that are predominantly long-chain fatty acids (LCFAs, ≥C14); animal fats that are mainly LCFAs; and industrial *trans*-fats [4]. Upon ingestion, medium-chain triglycerides (MCTs), which contain MCFAs, are hydrolyzed without the need for pancreatic enzymes, and MCFAs are absorbed and transported mainly through the portal vein to the liver, where they are partly converted to ketones, with the remaining MCFAs available to directly provide fuel to most tissues. MCFAs are not stored much in the adipose tissue and promote the browning of white fat [6]. Long-chain triglycerides (LCTs) require pancreatic enzymes for digestion and are hydrolyzed to fatty acids that are repackaged after absorption into chylomicrons and are transported in the blood stream [7]. LCFAs are not converted to ketones when consuming a typical high-carbohydrate diet but are stored in the adipose tissue [8,9]. The mitochondrial metabolism of MCFAs is more rapid than that of LCFAs. In particular, while MCFAs can passively diffuse across the mitochondrial membrane in the liver and kidney, LCFAs require carnitine palmitoyltransferase (CPT) and carnitine acylcarnitine translocase (CACT), which slow LCFA oxidation [10]. Recently, various saturated fats have been shown to have different metabolic effects (see below Section 2.3).

**Figure 1:**
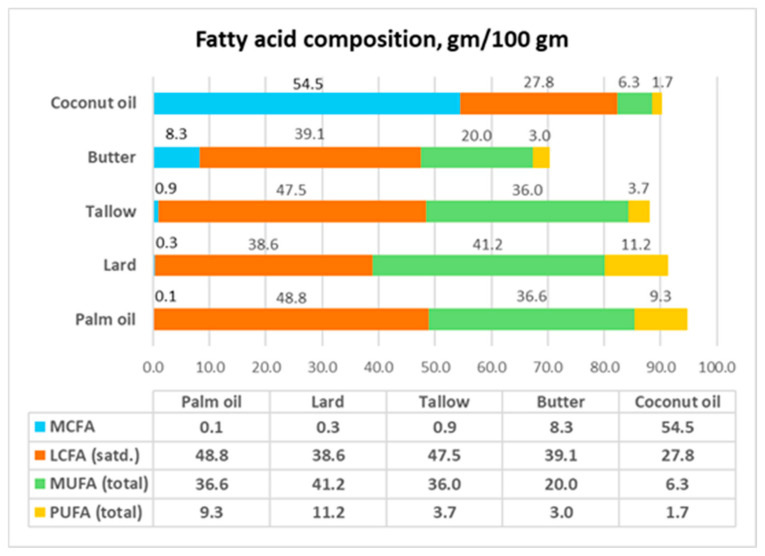
Comparison of fatty acid content of CNO and other so-called saturated fats, gm/100 gm. Data are from Table 1.

From 1980 to 2005, the *Dietary Guidelines for Americans* (DGA) conflated all types of natural saturated fat with industrial *trans*-fats and specifically mentioned CNO as a fat to avoid [4]. (*Trans*-fats, which were considered as saturated fat, will be discussed below.) Table 1 gives the fatty acid composition and cholesterol content of CNO and other plant oils and animal fats, and Figure 1 compares the MCFA, LCFA, monounsaturated fatty acid (MUFA), and polyunsaturated fatty acid (PUFA) content of the group of so-called saturated fats only. The fatty acid composition is given as (gm fatty acid/100 gm fat or oil), which is more accurate and quantitative than the %fatty acid profile, which is often used. The comparison shows the following points with respect to CNO.

- Only CNO can be considered a saturated fat, having total saturated fatty acid (SFA) content of 83 gm/100 gm. In comparison, the total SFA content of palm oil, lard, tallow, and butter is less than 50 gm/100 gm.
- Lard is commonly used as the control diet to represent saturated fat. However, lard has higher MUFA+PUFA content (52.4 gm/100 gm) than total SFAs (38.9 gm/100 gm). The predominant fatty acid in lard is oleic acid (C18:1) (41.2 gm/100 gm). This means that lard should be considered instead as an MUFA-SFA fat. This requires the reassessment of the conclusions from feeding and diet studies that used lard as a reference fat.
- CNO has an MCFA composition of 54 gm/100 gm. In contrast, none of the other members of this group has a significant amount of MCFAs.
- CNO contains 41.8 gm lauric acid (C12:0) per 100 gm, while butter, which is the next closest member, only has 2.6 gm C12:0 per 100 gm.
- The plant oils—CNO and palm oil—have 0 mg of cholesterol/100 gm, while the animal fats have high amounts of cholesterol. As will be discussed later, because the presence of cholesterol in animal fat may have metabolic effects on the gut microflora, plant oils and animal fats should not be directly compared regarding their metabolic effects.
- CNO and palm oil, which are assumed to be similar, have very different MCFA, saturated LCFA, MUFA, and PUFA compositions.

These differences show that the composition of CNO is significantly different from that of the other members of this group of so-called saturated fats. Likewise, the grouping of CNO and palm oil as “tropical oils” is a misleading label. Thus, the results of studies on animal fats and palm oil cannot be automatically extended to CNO. Coconut oil should be considered sui generis.

### 2.2. Effects of Dietary Cholesterol

Dietary cholesterol and heart disease were linked to saturated fat by Ancel Keys during his early studies in the 1950s [13,14]. The DGA warned against both saturated fat and dietary cholesterol from its first edition in 1980 until 2010. In 2010, the DGA discontinued the warning against cholesterol but retained the warning against saturated fat and further recommended a limit of 10 percent of the total calories. In 2020, the National Lipid Association Expert Panel concluded that the CVD risk of dietary cholesterol in normal individuals is modest, although the presence of hyper-responders should be recognized [15]. Recent epidemiological and clinical studies give a more favorable assessment of the effects of dietary cholesterol on blood lipids in terms of raising HDL-C [16]. Although the effects of dietary cholesterol on lipid parameters are now thought to be minimal, recent studies on germ- and pathogen-free obesity mouse models have shown that dietary cholesterol may have significant effects on the gut microbiome. A study comparing palm oil, which contains no cholesterol, and lard identified the effect of the cholesterol in lard on the gut microbiome as the likely mechanism for the opposite effects of palm oil and lard on obesity [17]. A recent review of the mechanisms of action of cholesterol on the gut microflora highlights the complex effects of dietary and plasma cholesterol [18], and an earlier study reported that dietary saturated fat and dietary cholesterol act independently on lipid parameters [19].

Since plant oils contain zero cholesterol, the results of studies using animal fat cannot be assumed to apply to plant oils, such as CNO and palm oil. Because lard is a common ingredient that is used as a reference material for saturated fat in dietary formulations in animal and clinical studies, the relevance of the results of these studies to CNO should be reassessed.

### 2.3. Medium-Chain Fatty Acids and Long-Chain Fatty Acids Are Metabolically Different

The generalizations about saturated fat fail to recognize that MCFAs, which are the predominant SFAs in CNO, are digested and absorbed differently and have biological properties that differ from the saturated LCFAs that are predominant in palm oil and animal fats. MCT oil, which is composed mainly of C8:0 and C10:0, has been used to treat malabsorption syndromes and malnutrition in infants through adults since the 1960s, and CNO has been added to most commercial infant formulas since the 1980s to provide the MCFAs found in human milk [9]. CNO and MCT oil are often used in the ketogenic diet and have been shown for more than a century to be effective in reducing or eliminating seizures in people with drug-resistant epilepsy. More recently, CNO and MCT oil, with or without a ketogenic diet, have been studied for people with age-related memory impairment, mild cognitive impairment, Alzheimer’s disease, and Parkinson’s disease, in which glucose hypometabolism is a factor, due the propensity of MCFAs to be converted to ketones, which provide an alternative fuel to glucose for the brain [20].

The generalization that CNO has the same metabolic effects as palm oil and animal fats is erroneous. More specifically, C12:0, the principal fatty acid in CNO, and palmitic acid (C16:0), the principal SFA in palm oil and animal fats (see Table 1), have been shown to have different physiological effects. C16:0 is common in the diet and is produced by the liver from excess carbohydrates through de novo lipogenesis. The high dietary intake of C16:0 has been shown in animal models to lead to hepatocyte lipid accumulation, which may cause steatosis [21]. Markers of liver injury and inflammation were significantly higher in mice that were provided the C16:0 diet, but not in those that were fed the C12:0 diet [22]. C16:0 triggers mechanisms linked with insulin resistance and inflammation, while C12:0 does not [23]. Because C16:0 tends to cause inflammation, it will also tend to raise LDL-C and lower HDL-C levels [24]. The higher inherent hepatic concentration of C16:0 compared to C12:0 means that the metabolic effects of palm oil (43.5 gm C16:0/100 gm) in feeding studies cannot be applied to CNO. CNO only has 8.6 gm C16:0/100 gm, which is comparable to the amounts found in MUFA and PUFA vegetable oils, such as corn, olive, safflower, soybean, and sunflower (Table 1).

### 2.4. Trans-Fat and Saturated Fat

Hydrogenation is a process in which vegetable oils are subjected to high heat and pressure using a catalyst, along with the injection of hydrogen gas, which transforms unsaturated fat to saturated fat, along with a complex mixture of *trans*-fatty acids with their positional isomers. Before the labeling of *trans*-fat was required in the US by the Food and Drug Administration (FDA) in 2006, industrial oils and fats and foods with these fats often contained considerable amounts of *trans*-fat. For example, one tablespoon of margarine (14 gm) could contain up to 2.4 gm *trans*-fat, shortening (14 gm) could contain 1.6 gm *trans*-fat, and a serving of cake with icing (74 gm) could have 7.2 gm *trans*-fat [25,26,27]. From the time that these were introduced into the food supply in the 1900s up to 2005, industrial *trans*-fats—in particular, margarine and shortening—were considered to be saturated fats as well because they were solid at room temperature [28,29]. Many studies that included both diet-controlled subjects and free-living populations were seriously flawed because these studies conflated “saturated fats” such as hydrogenated fats that contain *trans*-fat, such as margarine and shortening, with natural solid fats and oils, such as lard, butter, and CNO [4]. As early as 1953, Ancel Keys acknowledged that, based on USDA data, from 45 to 50 per cent of fats and oils in the US market were hydrogenated [30]. However, although he was aware that *trans*-fats raised TChol, he still used hydrogenated CNO in his feeding studies [31]. The use of shortening, margarines, and oils with significant amounts of *trans*-fat was widespread in US [26] and European households [32]. In 1995, a US expert panel on *trans*-fat reported that, “From the late 1960s until the mid-1980s, most household salad and cooking oils were prepared from partially hydrogenated soybean oil” [33]. It was only in 2018 that the FDA regulations limited, but did not completely eliminate, *trans*-fat from the US food supply [34].

In many feeding studies before 2000, solid margarine and partially hydrogenated oils with *trans*-fat were fed to test diet participants to replace saturated fat with polyunsaturated fat. The control groups consuming their usual diets and the test diet groups were all likely consuming *trans*-fat. The results of these studies should be reassessed.

### 2.5. The Problem of Intra-Individual and Inter-Individual Differences in Cholesterol Measurements

**Figure 2:**
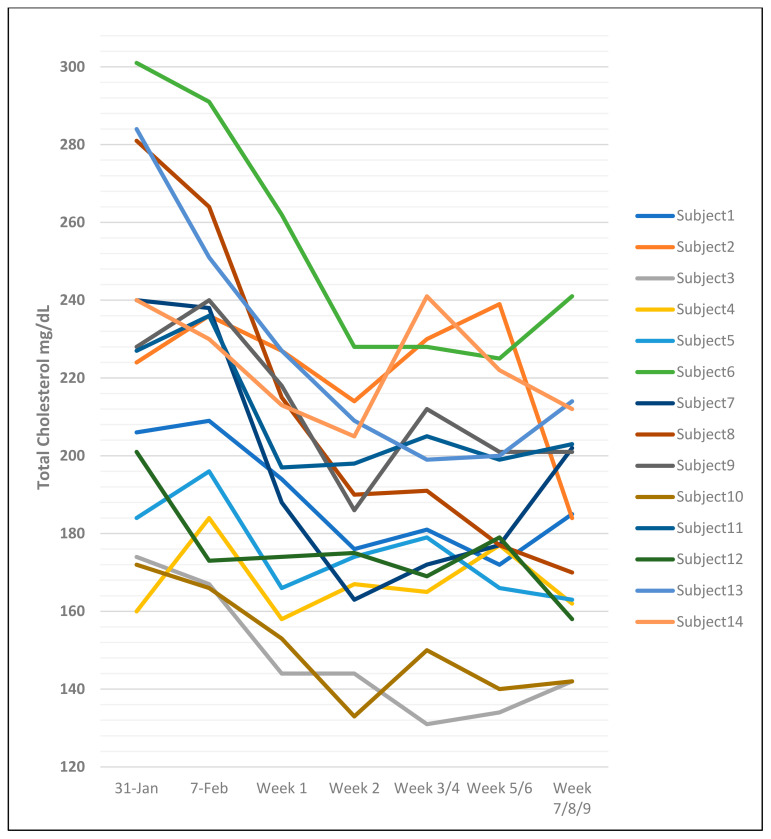
Total cholesterol levels for 14 men consuming a high-fat vs. a low-fat diet. This plot was produced from the data published in Table V of an article by Keys in 1957 including the dates [14]. The TChol results for the individual men were substantially different for January 31 and February 7, when all men were consuming an identical “house diet” with 37–42% of the total calories as fat. The results from week 1 onwards were obtained while the subjects consumed a “low-fat base diet” with 8–15% fat. Most men had a precipitous drop in TChol, reaching the lowest value between weeks 1 and 3, but it usually increased thereafter. These results show that the reliability of the data for individual prediction is low because of the large intra- and inter-individual variability.

Nearly every published dietary fat study reported by Ancel Keys and co-workers from the 1950s to the 1980s discussed the problem of intra-individual and inter-individual differences in TChol measurements. Even samples drawn at the same time from the same individual could have different results, and Keys noted an average of up to 12% variability in samples drawn a week apart from the same individual [14]. In the same paper, Keys used a study of 14 hospitalized men to illustrate the large variability in values within and between individuals receiving the same dietary fat intervention. The subjects were described as “clinically healthy men of the same age and engaged in the same activities and eating precisely the same diet”, which was abruptly decreased from 37–42% to 8–15% total fat (see Figure 2). The starting values for TChol ranged from 160 to 301 mg/dL, and the TChol values fell precipitously for most, but not all, of the men, reaching the lowest values between weeks 1 and 3, and appeared to be increasing by weeks 5 to 9, although this pattern was not true for all individuals. Three men with the highest baseline TChol of 280 to 301 mg/dL had the largest decreases during the first 2 to 3 weeks of the low-fat diet. The TChol levels for some men increased significantly from the lowest value at between 5 and 9 weeks, despite maintaining the very low-fat diet (see Figure 2). Keys stated in the discussion, “These predictions are for groups averages; the reliability of individual prediction is low because of the intra- and inter-variability noted above and because the magnitude (of the change in TChol) for a given dietary change tends to be related to the characteristic serum cholesterol level of the individual” [14].

### 2.6. Changes in Lipid Profile Values with Addition of Dietary Fat May Be Transient

Many dietary fat studies were conducted for just one to six weeks based on the assumption that the lipid profile had stabilized during that time. However, there are few reliable studies to show whether any differences in the lipid profile related to a change in dietary fat during the first few weeks are sustained if the intervention is continued for months or years and whether the response has clinical significance. The National Diet Heart Study (NDHS), which was conducted by Ancel Keys and others from 1963 to 1965, included 1807 male participants who had frequent lipid profile assessments for 6 to 18 months before and during assignment to several diets that compared the total percentage of fat and various ratios of PUFAs to SFAs but were also designed for weight loss, which can impact serum cholesterol. Participants received “fat-modified fabricated foods”, which included *trans*-fats. The average TChol levels decreased in all groups, with the maximum reductions at 2 and 6 weeks, but trended upward thereafter. Six months after the study was completed, 253 men from all diet groups had essentially returned to their original baseline values, which suggests that the effect of manipulating dietary fats may be temporary and that an individual’s metabolism may readjust over time to a genetically determined set point. There were no differences in adverse cardiac events between the control groups, who consumed a typical American diet, and the high-PUFA diet groups. However, the study was not powered to evaluate clinical outcomes [35]. The NDHS study was not renewed for the next phase.

### 2.7. Reviews of Prospective Studies Comparing Coconut Oil with Other Dietary Oils and Fats

The argument used to justify the recommendation to avoid CNO consumption is based mainly on studies that compared, as the primary outcome, the relative effects on the serum lipid profile between CNO and monounsaturated fatty acid (MUFA) and PUFA dietary oils. The underlying presumption is that reducing TChol and LDL-C by increasing PUFA and decreasing CNO intake will reduce the risk of CVD events and deaths. However, such comparisons do not actually assess CNO itself regarding its risk for CVD. Further, many of these studies were short-term—between 3- and 6-week durations—based on the assumption that the observed change in the lipid profile accurately predicted CVD outcomes. None of the studies were adequately powered and/or of a long enough duration to be able to claim a causal connection between the consumption of CNO and the increased incidence of cardiac events or mortality. For example, a long-term 2-year study that compared CNO and sunflower oil, a MUFA oil, used as a cooking oil reported no difference in CVD risk factors and events in 190 subjects with CAD [36].

### 2.8. Reviews of Interventional Studies Comparing Coconut Oil to Other Oils and Fats

Five recent reviews focused on studies involving CNO that were conducted from the mid-1980s to the late 2010s. In a 2016 review, Eyres et al. [37] narrowed their analysis to just eight heterogeneous interventional studies comparing CNO to one or more oils. The feeding periods lasted from only 9 days to 6 weeks, with 9 to 54 participants, and included two studies that used a test oil that was supposed to be CNO but had a fatty acid composition of 36.8% C12:0 and 11.0% oleic acid (C18:1) [38,39], which deviates significantly from the median values given in the Codex Alimentarius of 49% and 7.5%, respectively [12], suggesting possible contamination. Eyres pointed out several inconsistencies in the results of the selected studies and noted methodological flaws in some of the studies and the limited number of interventions. They asserted that studies of traditional populations consuming CNO with low CVD incidence do not prove causation, since many other factors could be involved, such as the effect of the consumption of other traditional foods along with coconut meat and the low consumption of processed food, which is a common feature of Western diets. Nevertheless, they concluded that, “Collectively, these results do not provide evidence that CNO acts consistently different from other saturated fats in terms of its effects on blood lipids and lipoproteins”. Conversely, the results also do not show that CNO acts in the same way as other saturated fats or that CNO is harmful to health outcomes.

The systematic review and meta-analysis by Schwingshackl et al. (2018) [40] provided tables showing estimated differences in effect for TChol, LDL-C, HDL-C, and TG values between 13 different dietary oils and fats, including CNO, in 55 comparison groups, totaling 2065 participants from 54 randomized control trials (RCTs) reported between 1984 and 2018, lasting from 3 to 27 weeks. The comparisons were performed based on the substitution of one oil or fat for another at 10% of the daily energy. Of the 150 comparisons, there were only 26 instances of statistically significant differences for LDL-C and TG and 39 for HDL-C for all oils and fats combined. There were comparisons of CNO with 12 other oils and fats. CNO had no significant effects on LDL-C or TG at a 95% confidence interval, but CNO had the greatest effect of increasing HDL-C among all fats and oils, which included butter, lard, beef fat, and vegetable MUFA and PUFA oils. A second review by Schwingshackl et al. (2023) [41] reported that CNO had no significant effects on LDL-C and TG but had favorable effects on HDL-C compared with other oils and fats.

Unhapipatpong et al. (2021) [42] combined the results of nine meta-analyses, including some studies involving CNO, and reported that the “replacement of PUFAs with CNO significantly increased HDL-C and total cholesterol by 2.27 (0.93–3.6) mg/dL and 5.88 (0.21–11.55) mg/dL, respectively—but not LDL-C”. They concluded that the “replacement of unsaturated plant-derived fats with plant-derived saturated fats slightly increases LDL-C but also increases HDL-C, which in turn may exert a neutral effect on cardiovascular health”.

Neelakantan et al. (2020) [43] conducted a review and meta-analysis that included 17 studies reported in 16 articles that compared CNO with other dietary oils. This review focused mainly on the effects of the oils on the lipid profile. However, this review included three studies that did not use CNO as the test oil. In particular, the review included a study that used “fractionated” CNO, a C8:0/C10:0 blend of MCT oil, which was not CNO [44], as well as two studies that used a purported RBD CNO with unusually low C12:0 and high C18:1 content, suggesting that the test oil used may have been contaminated [38,39]. In addition, Figure 1 and Figure 2 in the Neelakantan review graphed the results for LDL-C and HDL-C as differences in mg/dL between the values for CNO and the average values of up to three comparator oils. The values were labeled in Figure 1 as “Coconut oil increases LDL-C” and “Coconut oil decreases LDL-C”; however, the data reported are relative differences in the performance of the oils, rather than the absolute differences from the baseline values for the CNO group. For example, a weight loss study in which women consumed 6 g per day of CNO versus other oils was reported in Neelakantan’s Figure 1 as showing an increase in LDL-C of +9.67 mg/dL, although the change in LDL-C reported by the reference was −14.89 mg/dL [45]. Likewise, Figure 2 of the Neelakantan study showed that “CNO decreased HDL-C” in the study by Vijayakumar et al. (2016) [36]. However, the Vijayakumar study actually reported that HDL-C increased at 2 years for the CNO group by +2.42 mg/dL, while, for the comparator sunflower oil (SunO) group, the increase was +3.62 mg/dL [36]. Thus, the authors reported the comparison as a decrease in HDL-C for CNO when the actual result was an increase in HDL-C. Neelakantan concluded that CNO is “hypercholesterolemic” because “it significantly increased LDL-C as compared with nontropical vegetable oils”, with a recommendation of “limiting coconut oil consumption because of its high saturated fat content” [43].

It is important to note that many studies included in the Neelakantan review showed large standard deviations (SDs) between the baseline and final values of CNO of ±20 to 47 mg/dL for TChol [36,38,39,46,47,48,49,50] and ±15 to 45 mg/dL for LDL-C [36,38,39,46,47,48,49,50,51]. These large SDs suggest that there were considerable inter-individual differences within the groups. In addition, the average baseline and final results for TChol in 10 CNO studies were below 200 mg/dL [36,38,39,45,47,48,51,52,53,54], which is considered to indicate low cardiac risk.

### 2.9. Studies of Populations That Consume Coconut Oil as a Staple

Many studies on dietary oils are conducted on subjects who do not normally consume the oils that are being studied and are asked to take the test oil for a limited time period. Studies of people who have consumed specific dietary oils as staples are often overlooked, yet they offer far more reliable evidence than short-term studies for the impact of dietary fat on lipid levels and, more crucially, on overall health outcomes. The following are two examples of recent studies of people consuming CNO versus sunflower oil (SunO), a high-MUFA oil (see Table 1), as a staple.

Sabitha et al. (2009) [55] studied 140 people from Kerala, India, where coconuts are plentiful and CNO is commonly used in the diet. Half of the subjects had been consuming CNO and the other half SunO as their main cooking medium for at least 6 years and obtained 13 to 20% of their total calories from the oil studied. The subjects were further divided into two groups of 35 each: one group of healthy people without evidence of heart disease (average age 45) and another group with type 2 diabetes but without heart disease and not taking lipid-lowering drugs (average age 55). All healthy people in the CNO group had TChol less than 200 mg/dL (see Table 2). The average LDL-C levels were higher and the HDL-C values were lower for the SunO healthy and diabetes groups compared with the corresponding CNO groups. The study concluded that the habitual dietary consumption of CNO “has no specific role in the causation of coronary heart disease in the Kerala population”.

Due to concerns about the high incidences of CAD, diabetes, hypercholesterolemia, and hypertension in Kerala, India, Palazhy et al. (2018) [56] performed a study to determine whether CNO, which is commonly used in Kerala, might be a contributing factor to these conditions. To compare the long-term effects of the dietary intake of CNO and SunO, a study was conducted on men aged 35 to 70 and taking cholesterol-lowering drugs who either had a documented myocardial infarction or angiographically proven CAD. The men were divided into two groups: those who had either been consuming CNO (n = 73) or SunO (n = 80) as their main dietary oil for at least 2 years. Lipid profiles were obtained, along with markers of lipid peroxidation—in particular, oxidized LDL-C and malondialdehyde (MDA), the levels of endogenous antioxidants (glutathione, glutathione peroxidase, and superoxide dismutase), and the antioxidant vitamin C. The ages and the percentages of people with diabetes, hypertension, and smoking were not different between the groups, and all men were consuming a typical high-carbohydrate Keralan diet with 13 to 20% of the total calories from the oil of interest (see Table 3). At the end of the study, there were no significant differences in TChol, LDL-C, oxidized LDL-C, ApoB, or any of the antioxidant levels between the two groups. However, the rate of lipid peroxidation as indicated by the MDA levels was found to be higher in the men consuming SunO. HDL-C was higher in the CNO group, but the difference was not significant. This study concluded that the men consuming CNO had comparable lipid profiles and did not show hypercholesterolemia compared to the men consuming SunO.

The results of the Sabitha (2009) [55] and Palazhy (2018) [56] population studies are consistent with a two-year prospective study by Vijayakumar (2016) [36] of 190 men with CAD and taking statins, which found no significant differences in lipid profile values at any time point (3 months, 1 year, and 2 years) between men consuming CNO and SunO. (See the Results section for a detailed presentation of Vijayakumar 2016).

In 2012, Palazhy et al. published a study [57] in which endarterectomy and plasma samples were taken from 71 men who were undergoing coronary artery bypass surgery and were consuming either CNO or SunO as their staple cooking medium. All the men in the CNO group had consumed CNO since early childhood, whereas the men in the SunO group had switched to SunO for at least one year and, on average, for 46 months. All participants were receiving lipid-lowering therapy, and some men were also receiving medications for blood glucose and/or blood pressure control. The dietary concentrations of C16:0 and C18:0 were essentially the same in both groups, and the percentage of total SFAs in the plasma was not significantly different between the CNO group (71%) and the SunO group (66%). The plasma concentration of linoleic acid (C18:2) was higher in the SunO group (14.3% vs. 8.7%), and myristic acid (C14:0) was higher in the CNO group (11.2% vs. 5.1%). MCFAs were found in low concentrations in the plasma and plaques in both groups, and there were no significant differences in the plasma MCFA concentrations between the groups, despite the high C12:0 intake of the men in the CNO group. LCFAs dominated the lipid content of the plaques in both groups, and the fatty acid composition of the plaques did not differ significantly between the CNO and SunO groups [57]. These studies show that the consumption of CNO as a staple for years does not give a less favorable lipid profile than a high-MUFA oil, such as SunO, and, as a traditional part of the tropical diet for millennia, it provides a healthy lipid pattern that does not increase the cardiac risk. 

### 2.10. Studies on High-Omega-6 Diets

The most consistent recommendation of the DGA is to replace saturated fat with unsaturated fat. Contrary to expectation, two large long-term studies that tested the effects of high linoleic acid (C18:2) consumption showed that C18:2 significantly increased the rates of all-cause and heart disease-related mortality [58,59]. However, these studies, which were both completed in 1973, were not reported contemporaneously before the first edition of the DGA was published in 1980.

The Sydney Diet Heart Study (SDHS) was conducted in Sydney, Australia (1967–1973), in 458 men aged 30 to 49, using high C18:2 safflower oil (SaffO) (see Table 1) as a test oil. The control group (n = 237) maintained their usual diets, including the margarines that they were already consuming. The SaffO group (n = 221) was instructed to use only oils, margarines, and shortening produced from SaffO in place of all foods containing “saturated fat” to increase their PUFA intake to about 15%, to reduce the saturated fat to less than 10% of the total calories, and to limit their dietary cholesterol intake to less than 300 mg/day. Despite a larger decrease in TChol in the high-PUFA SaffO group (−37.4 mg/dL) compared to the control (−15.5 mg/dL), the men consuming the SaffO diet had significantly higher rates of all-cause death (17.6% vs. 11.8%, HR1.62), cardiovascular death (17.2% vs. 11.8%, HR 1.70), and fatal CHD (16.2% vs. 10.1%, HR1.74). However, the results of the SDHS were not published until 2013, 40 years later, from recovered raw data [58]. An updated meta-analysis in the same article showed no evidence of cardiovascular benefit from the replacement of saturated fats with polyunsaturated fats.

The Minnesota Coronary Experiment (MCE) (USA, 1968 to 1973) included 9423 men and women aged 20 to 97 living in nursing homes or mental hospitals, of whom half were assigned to the corn oil test group and the other half to the control group [59]. The control group consumed the usual hospital diet with about 18.5% saturated fat, including hydrogenated and partially hydrogenated fats and oils. The test group received corn oil margarine in place of butter and corn oil for food preparation, thereby reducing their saturated fat intake by half to 9.2%. Corn oil contains about 54% C18:2, and the omega-6 intake of the subjects increased almost three-fold to 13.2% of the total calories. As anticipated, the average TChol level decreased from the baseline much more in the corn oil group (−31.2 mg/dL) than in the control group (−5.0 mg/dL), but there were more cardiac events and deaths in the corn oil group. People with the largest reduction in TChol had the highest incidence of death. Unfortunately, the results of the MCE study were not published after its completion in 1973. Instead, partial results were published in 1989 by Ivan Frantz, Jr. as the principal author, without his co-principal investigator, Ancel Keys, as a co-author [60]. Frantz reported that there were 27.2 incidents of MI and sudden death per 1000 person-years for the corn oil group and 25.7 incidents for the control group, and the total deaths amounted to 55.8 per 1000-person years for the corn oil group and 52.6 for the control group. A more complete analysis of the MCE study was conducted by Ramsden and co-workers from recovered raw data [59]. They found that, for the 2355 subjects who consumed the corn oil diet for more than one year, there was a 22% higher risk of death for each 30 mg/dL reduction in TChol. Detailed autopsy reports on 149 subjects showed that 41% of those in the corn oil group had evidence of at least one myocardial infarction, compared with only 22% in the control group. In addition, the corn oil group did not have less atherosclerosis in the coronary arteries and aorta than the control group, and “there was no association between serum cholesterol and myocardial infarcts, coronary atherosclerosis, or aortic atherosclerosis in covariate adjusted models”. The findings from the recovered MCE results are consistent with other autopsy studies that failed to show that replacing saturated fat with polyunsaturated fat reduces atherosclerosis or atheroma formation [61,62]. An accompanying systematic review of five RCTs on the replacement of saturated fat with high-C18:2 vegetable oils in 10,808 participants also found no benefit for CHD mortality or all-cause mortality and no benefit for the prevention of non-fatal myocardial infarctions or for fatal and non-fatal myocardial infarctions combined. Ramsden et al. concluded that the “available evidence from randomized controlled trials shows that replacement of saturated fat in the diet with linoleic acid effectively lowers serum cholesterol but does not support the hypothesis that this translates to a lower risk of death from CHD or all causes” [59].

No subsequent large long-term studies have been conducted to determine whether consuming natural saturated fat, including CNO, results in a greater risk of CAD or CVD than high-C18:2 oils. On the contrary, these long-term studies show that high-C18:2 diets raise the risk of death from heart disease, rather than high-saturated-fat diets.

## 3. Analysis of 26 Coconut Oil Studies

Most interventional studies and reviews on CNO have compared its effects on the lipid profile with those of other dietary oils and fats. This is the first review that provides a comprehensive analysis of a large number of studies of people consuming CNO itself, rather than in comparison with other oils and fats, to assess changes in their lipid profiles from the baseline to final values, further categorized by study duration.

### 3.1. Objective

The objective of this review is to assess four decades of dietary intervention research on CNO consumption, specifically examining changes in lipid parameters such as TChol, LDL-C, HDL-C, TG, and their ratios to HDL-C. This reassessment also aims to determine whether the durations of the studies influence the lipid responses.

### 3.2. Search for Studies

**Figure 3:**
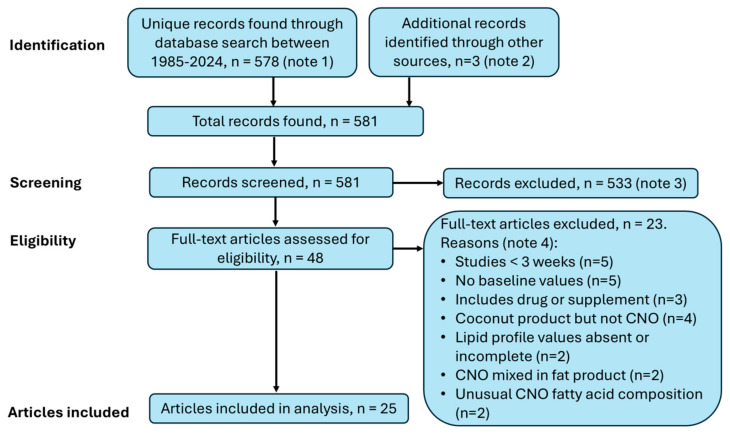
Flow diagram of literature search. Note: Twenty-six studies are reported in 25 articles

For this analysis, a PubMed.gov search was undertaken for studies of CNO reported between 1985 and 2024 using the terms “coconut oil” and “cholesterol”. This search produced 578 results (note 1); three additional results for consideration were found from other sources (Cochrane Library and previous reviews) for a total of 581 results (note 2). From this list, the following were excluded: animal and in vitro studies; studies of people under 18 years; systematic reviews of multiple studies; commentaries, opinions, letters, and case reports; reports that mentioned CNO but did not study the oil; studies that did not include the consumption of CNO, such as through topical application or an oral rinse (note 3); single-dose studies; studies with a duration of less than 3 weeks; studies of specific fatty acids rather than whole coconut oil; studies of medium-chain triglyceride (MCT) oil, which is mainly C8:0 and C10:0; studies with large deviations from the standard CNO fatty acid profile; studies in which coconut oil was part of a mixed oil product; studies that combined CNO with the initiation of a drug or supplement as an intervention; studies of coconut oil as an intervention for serious acute infection; studies of populations taking CNO for years that did not have baseline values for comparison; and studies of CNO that did not report the measurement of all four lipid profile values for a CNO test period: TChol, LDL-C, HDL-C, and TG (note 4). A search of the Cochrane Library using the terms “coconut oil” and “cholesterol” did not find any additional studies that met the criteria and were completed and reported in full-text articles. See Figure 3 for a flow diagram of the literature search.

Altogether, 26 studies reported in 25 articles were included in this analysis, which were conducted from 3 weeks to 2 years. The 26 studies included 29 sets of complete results, with a total of 984 complete data sets with baseline and final lipid profile values for a test period in 792 distinct individuals. A study by Cox et al. (1995) [63] reported separate results for 13 males and 15 females, yielding two groups of lipid profile data sets. The study by Vijayakumar et al. (2016) [36] provided three separate groups of data sets for 96 participants at 3 months, 1 year, and 2 years, and the baseline lipid values at the beginning of the entire study were used for all calculations from this study. In addition, the study by Swarnamali et al. (2024) [64] reported both calculated and direct measurements of LDL-C, and the direct measurement was used for this analysis since this method is considered to be more accurate. The list of the 26 CNO studies is given in Table 4. File S1 provides a narrative description of each study.

It should be noted that, before 2000, most studies used refined, bleached, and deodorized coconut oil (RBD CNO), which is obtained from copra, the dried meat of the coconut. Virgin coconut oil (VCO), which is obtained from the fresh meat without chemical treatment [77], became popular after 2000 and was used in most of the recent studies. The effect of processing on the quality of the coconut oil is significant. Further, some studies provided the CNO with meals, while other studies provided it as a cooking oil.

Six of the 26 studies in our analysis were not randomized, including two that were crossover studies [65,69], two that were sequential feeding studies [64,72], and two studies with parallel groups [54,66]. Of the 20 randomized studies, eight were RCTs using a parallel design [36,45,47,68,70,74,75,76], and the remaining 12 were crossover studies. Altogether, 16 of the studies were crossover or sequential diets in which individuals received more than one intervention in different time periods. By design, individuals serve as their own controls in crossover studies, which helps to reduce intra-individual variability, as well as the number of participants required to achieve significance.

### 3.3. Calculations

Average results were calculated for TChol, LDL-C, HDL-C, and TG in all studies combined, as well as for short-duration studies that lasted 3 to 7 weeks, medium-duration studies that lasted 8 to 24 weeks, and a long-duration study with lipid profile values available at 1 and 2 years. The long-duration study also provided lipid profile results at 3 months, which were included with the medium-duration studies. For uniformity, the data for all values were converted as needed from mmol/L to mg/dL using an online calculator (https://www.mdapp.co/cholesterol-conversion-calculator-600/, accessed on 19 September 2024), which multiplies the values in mmol/L by 38.66976 for TChol, LDL-C, and HDL-C and multiplies the value in mmol/L for TG by 88.57396 to arrive at the values in mg/dL.

The method used to calculate the average values ensured that the values for individual participants would have equal weight when the results of the studies were combined. To arrive at the combined average differences in lipid values from baseline to the end of each test period, the average baseline values were entered into an Excel spreadsheet for each group and then multiplied by the number of participants in that group. Likewise, the average end-of-test-period values were entered for each group and then multiplied by the number of participants in that group. The totals for the baseline and final values for each group were summed separately and divided by the total number of participants in the combined groups to arrive at the absolute difference and the percent difference between the average baseline and average final values for all studies combined. The same method was used to determine the results separately for long-, medium-, and short-duration studies. A double check was performed by finding the difference between the baseline and final values for each group and then multiplying it by the number of participants in each group. The results for each group were summed and divided by the total number of participants to arrive at the average difference and percent difference for all studies combined, as well as separately for long-, medium-, and short-duration studies. This value was compared to the value for the previous alternate calculation outlined above to show agreement. Cox et al. (1998) [72] reported separate baseline results for lipid values in males and females, but the final values were reported for both males and females combined; the average baseline lipid values were combined for men and women using the same calculation to ensure equal weighting, and this was used to determine the differences between the average baseline and final values.

Standard deviations (SDs) for all baseline and final values for each group as reported in the articles for each study were entered and were then added and subtracted from the baseline and final values to show the lowest and highest values within the SD range. Maki et al. (2018) [73] used medians and interquartile ranges (IQRs) to report data, rather than averages and SDs, and these were noted in the spreadsheet. A few studies did not report SDs. The median was used in place of the average for the Maki (2018) calculations since the raw data were not available to calculate the average. Khaw et al. (2018) [49] also used the median and IQR values for the baseline TG value but not for the final value. Averages and SDs were used for all other values in this study.

## 4. Results of the Analysis of the 26 Coconut Oil Studies

The objective of this analysis was to study the lipid profile responses of people adding CNO to their diet under a variety of conditions. This was not intended to be a formal meta-analysis, which requires more rigorous criteria. 

### 4.1. Heterogeneity of the Studies

The 26 studies included in this analysis were quite heterogeneous and were conducted over a nearly 40-year span in Asian, Asia-Pacific, European, Middle Eastern, North American, and South American populations, where the habitual diets would have differed greatly and may have changed substantially during this timeframe. Some studies included only men and others only women. The inclusion criteria of the studies varied: some studies accepted only young healthy people with a normal BMI or only older people with diagnosed dyslipidemia or obesity, with all subjects taking statins or none taking statins. Some studies included free-living people, preparing their own meals with the oil provided but with no other dietary instruction or restrictions, whereas others studied participants who were confined or closely monitored and were required to consume the same prepared meals. CNO represented only part of the fat consumed, either a fixed amount or based on the participant’s weight or a percentage of the total fat or total calories consumed, and the other fats consumed varied from study to study or were not usually reported. Some studies were multi-interventional studies that included dietary instruction, a reduction in energy intake to encourage weight loss, or exercise, and two studies combined the oil intervention with statin treatment. At least five studies reported in four articles supplied the test oil in muffins, rolls, or cookies, which added carbohydrates and calories to the diets [46,52,53,73]. For example, in two studies (McKenney et al. (1995) [46]), the muffins and cookies added ≥600 kcal to the baseline energy intake, resulting in substantial weight gain averaging about 2.25 kg in the CNO and canola oil groups.

The amount of CNO consumed varied from 6 g per day to as much as 117 g per day. The processing methods of the oils were not reported for most studies, and fatty acid compositions were not provided in some studies. Many were crossover studies, some with and some without a washout period and with or without a new baseline at the beginning of each test oil period. Some crossover studies used only the baseline at entry for the calculations of each test oil period and did not separate the results based on the order of the test oil periods.

Rather than the direct measurement of LDL-C, most studies used the Friedewald formula, a calculation method [78], which is known to be of limited accuracy since the formula assumes a fixed ratio between TG and VLDL-C. However, this ratio can vary significantly among individuals, especially in cases of dyslipidemia and hypertriglyceridemia, leading to inaccurate LDL-C estimates [79]. Among the 26 studies, only five studies excluded people with dyslipidemia (Reiser 1985 [52], Chinwong 2017 [67], Korrapati 2019 [54], Jeyakumar 2023 [69], Vogel 2020 [74]), while six other studies reported in five articles specifically recruited people with dyslipidemia and used the Friedewald formula instead of direct measurement (McKenney 1995 [46], Cardoso 2015 [66], Maki 2018 [73], Nikooei 2021 [75], Setyawati 2023 [76]).

### 4.2. Coconut Study Data Available for Analysis

Altogether, there were 984 pairs of lipid profile values drawn at baseline and at the end of a test period available for 792 distinct participants consuming CNO as a dietary intervention in 26 studies with 29 distinct test period groups. The Vijayakumar study (2016) [36] provided three test period groups for 96 individuals at three months, one year, and two years, and the Cox study (1995) [63] provided two separately reported groups for males and females.

### 4.3. Results

#### 4.3.1. Overview of Results

Table 5 summarizes the differences in the TChol, LDL-C, HDL-C, and TG values before and after consuming CNO for all studies combined and for short-, medium-, and long-duration studies. (See File S1 for a detailed narrative description of each study and File S2, Table S1 with more detailed results for each study. File S3 Tables S2–S5 contains the complete data set of lipid profile values used in the analysis.

**Figure 4:**
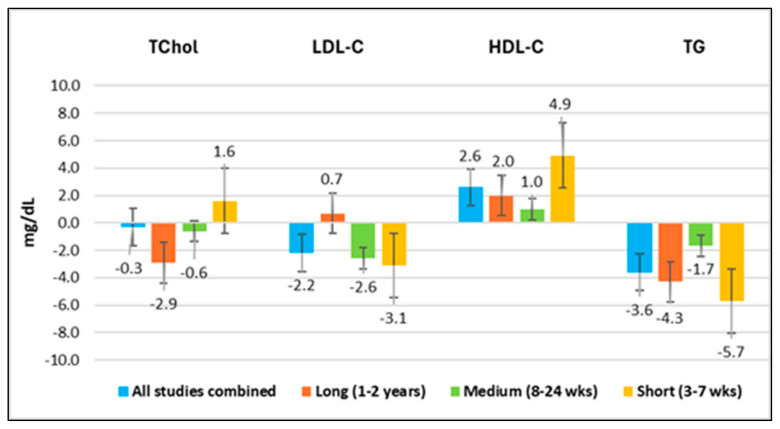
Differences in TChol, LDL-C, HDL-C, and TG in people consuming CNO in short-, medium-, and long-duration studies. Data are from Table 5.

Figure 4 plots the differences in the TChol, LDL-C, HDL-C, and TG values of the study duration groupings. The following observations can be made for persons consuming CNO:

1. HDL-C increased in all time periods;
2. TG decreased in all time periods;
3. TChol and LDL-C showed both increases and decreases in different time periods. The wide variability in these two lipid values makes TChol and LDL-C less reliable measures.

#### 4.3.2. All Studies Combined

When all 29 groups were combined, there were 984 pairs of baseline and final lipid values. TChol decreased on average by −0.3 mg/dL (−0.13%), LDL-C decreased by −2.2 mg/dL (−1.8%), HDL-C increased by +2.6 mg/dL (+5.8%), and TG decreased by −3.6 (−2.9%). Table 5 gives the absolute results, while Table 6 tabulates the changes in the lipid profiles. These results are plotted in Figure 4. For TChol, only 7 of 29 groups showed an increase of ≥5%. For LDL-C, just 7 of 29 groups showed a ≥5% increase, of which five groups had a ≥10% increase. For HDL-C, 22 of 29 groups had an increase, with 17 groups showing an average increase of ≥5%, of which eight groups had an increase of ≥10%. For TG, only 6 of 29 groups showed an increase of ≥5%, of which just two groups had an increase of ≥10%.

#### 4.3.3. Short-Duration Studies (3 to 7 Weeks)

There were 350 pairs of lipid profile values available for 16 groups of people before and after consuming CNO in test periods of 3 to 7 weeks (see Table 5). There was a trivial average increase of +0.8% in TChol and a more significant average increase of +10.0% in HDL-C. LDL-C and TG showed decreases of −2.3% and −4.3%, respectively.

For TChol, only 5 of 16 groups showed an average increase of ≥+5% in TChol, of which just two groups showed values ≥+10%. For LDL-C, 6 of 16 groups showed an average increase of ≥+5%, of which four groups showed values ≥+10%. Twelve of 16 groups showed an increase in HDL-C, with 11 groups ≥+5%, of which seven groups had an increase of ≥+10%. Ten of the 16 groups had an average decrease in TG, and just two groups had an increase of ≥+10% (see Table 6).

A subgroup of eight short-duration studies lasting 3 to 4 weeks reported highly disparate average differences in LDL-C, ranging from −2.57% to +24% (see studies indicated as § in Table 6). The amount of CNO consumed in these studies ranged from 16 to 60 mL daily, and there was no apparent association between the change in LDL-C levels and the amount of CNO consumed. For example, people taking CNO 30 mL in three different studies had differences in LDL-C of −2.6%, +10.9%, and +19.5%, whereas a group consuming 50 mL CNO had a decrease in LDL-C of −2.6%. This suggests that the effect of adding a new oil, such as CNO, to the diet may be highly variable from person to person.

#### 4.3.4. Medium-Duration Studies (8 to 24 Weeks)

There were 442 pairs of lipid profile values available for 11 groups of people before and after consuming CNO in test periods of 8 weeks to 24 weeks (see Table 5). There was an average decrease of −0.31% for TChol and −2.3% for LDL-C, an increase of +2.3% for HDL-C, and decrease of −1.4% for TG (see Table 5).

For TChol and for LDL-C, only 1 of 11 groups had an increase of ≥+5%. For HDL-C, 5 of 11 groups had increases of ≥ +5%, and only two groups showed decreases. For TG, 6 of 11 groups had average decreases, and only one group had an increase of ≥+5% (see Table 6).

#### 4.3.5. Long-Duration Study (1 to 2 Years)

One study of 96 people lasted two years and provided pairs of lipid values at baseline, three months, one year, and two years (Vijayakumar 2016 [36]). The original pre-study baseline values were used for all calculations (see Table 3). The pairs of lipid values for the 96 people before and after consuming CNO for three months were included in the medium-duration study results. There were two additional groups of data for the 96 people, totaling 192 pairs of lipid profile values before and after consuming CNO for one year and for two years. TChol decreased on average by −5.23 mg/dL (−3.5%) after one year and by −0.61 mg/dL (−0.4%) after two years. LDL-C increased on average by just +0.73 mg/dL (0.81%) after one year and by +0.75 mg/dL (0.83%) after two years. HDL-C increased on average by +1.61 mg/dL (3.95%) after one year and by +2.42 mg/dL (5.93%) after two years. TG decreased by −2.96 (−2.6%) after one year and by −5.64 (−4.9%) after two years (see Table 5 and Table 6).

### 4.4. Wide Range of Average Lipid Profile Values and Standard Deviations Between Studies

There was a wide range of baseline and final lipid values, differences, and SDs between the 29 groups combined (see Table 7), even when separated into short-, medium-, and long-duration studies (see Table 8). The range of differences in TChol for short-duration studies of −21 to +33 mg/dL resulted in a spread of 54 mg/dL, compared to a range of differences for medium-duration studies of −19 to +14 mg/dL, with a smaller spread of 33 mg/dL, and there was a minimal difference at 1 and 2 years in the long-duration study. Likewise, for LDL-C, the range of differences for short-duration studies of −38 to +25 mg/dL resulted in a spread of 63 mg/dL, compared to a range for medium-duration studies of −13 to +15 mg/dL, with a smaller spread of 28 mg/dL, and essentially no difference at 1 and 2 years in the long-duration study. In File S2, Table S1 shows, for each of the 29 groups, the baseline lipid profile values, the differences between the baseline and end-of-test-period values while consuming CNO, and the SDs reported by the investigators for the differences in the lipid profile values.

The baseline and final lipid values, differences, and SDs in people consuming CNO varied widely among the studies, suggesting that there was great variability in the responses of individuals to the addition of CNO to their diet. The range of differences in TChol and LDL-C was essentially halved between the short- and medium-duration studies, indicating that many people experience large initial increases or decreases in their lipid values that might trend back toward their baseline values over time. In these same studies, people consuming other dietary oils and fats to look at the effects on lipid parameters also experienced a wide range of responses, as indicated by the large SDs reported by the authors. This strongly suggests that the lipid profile values may not stabilize within a few weeks or even a few months when a new oil, such as CNO, is added to the diet. Therefore, it is not possible in a clinical situation to predict how any given individual will respond to adding CNO to the diet. Monitoring the lipid response periodically would be reasonable for individuals who are considered at risk.

### 4.5. Similar Small Studies Can Have Different Results

The studies of Khaw et al. (2018) [49] and Harris et al. (2017) [50] illustrate the variability in the lipid response when adding CNO to the diet in a short-duration study. Both were small studies lasting just four weeks, in middle-aged people consuming their usual diets supplemented with various oils to study the effects on the lipid profile (see Table 6). In the Harris (2017) study [50], there were just 12 participants, who received 30 mL per day of VCO during one phase of a crossover study with a washout period, and they experienced significant increases in TChol, LDL-C, and HDL-C and an insignificant decrease in TG. The Khaw (2018) study [49], which had 28 participants consuming 50 mL per day of VCO, lasting four weeks, reported a smaller average increase from baseline in TChol, a decrease in LDL-C, and a larger increase in HDL-C than in the Harris study. The combined values for the 40 people showed an average increase in TChol of +11.4 mg/dL (5.0%), an increase in LDL-C of +1.62 mg/dL (1.2%), and an average increase in HDL-C of +9.56 mg/dL (13.0%) (see Table 9). This shows that the increase in HDL-C contributed much more than the small increase in LDL-C to the higher average TChol levels. The SDs for TChol and LDL-C were large in both studies. One might conclude, from a comparison of these two studies, that consuming a larger amount of VCO results in a more desirable lipid profile; however, the large SDs in both studies suggest that the results were highly variable between individuals and that these studies alone or even in combination are not adequately powered to come to such a conclusion with a reasonable degree of certainty.

### 4.6. Lipid Ratios

Historically, only TChol and later LDL-C were used to assess the risk of heart disease because they are easy and relatively inexpensive to measure [80]. However, lipids interact in complex ways, and focusing only on these two parameters does not provide an accurate assessment of the cardiovascular risk. Examining lipid parameters more comprehensively gives a more complete picture of the impact of a person’s lipid metabolism on heart health [81]. Lipid ratios are more informative than individual lipid values because they contextualize the individual lipid parameters in relation to each other. Lipid ratios using HDL-C as the common denominator are particularly useful [82] since HDL-C is the common value to which the other lipid parameters are compared. A greater increase in HDL-C relative to other lipid parameters gives a negative value for the difference in the ratios between the baseline and final conditions.

An analysis of the records of 177,860 patients aged 50 to 89 who were followed for up to 10 years (average 6 years) found a U-shaped distribution in the LDL-C values versus mortality rates. The study reported hazard ratios (HR) of 0.87 to 0.91 for three LDL-C groups between 100 and 189 mg/dL compared to the LDL-C reference group of 80–99 mg/dL (HR 1.0), an HR of 1.23 for LDL-C levels less than 80 mg/dL, and an HR of 1.19 for LDL-C levels greater than 189 mg/dL. These HRs translated to a cumulative 10-year mortality rate of 19.8% for people with baseline LDL-C levels of 30 to 79 mg/dL and 14.7% for levels from 80 to 99 mg/dL, compared to the cumulative mortality of 11.7% for levels from 100–129 mg/dL, 10.7% for levels from 130 to 159 mg/dL, and 10.1% for LDL-C levels from 160 to 189 mg/dL. The cumulative mortality then increased to 14% for LDL-C levels over 189 mg/dL. The distribution of the LDL-C values versus the cumulative 10-year rates of atherosclerotic CVD was similarly U-shaped and was the lowest for LDL-C levels between 100 and 189 mg/dL. These results question the current recommendations for statin treatment based on LDL-C levels. In contrast, both the TChol/HDL-C and TG/HDL-C ratios gave better associations with long-term mortality. A TChol/HDL-C ratio greater than 6.0 had a significantly higher risk of mortality than a ratio of 3.0 or lower. Likewise, the TG/HDL-C ratio showed a progressively lower mortality risk with each quintile of lower ratios, with the greatest risk at 3.44 or above and the lowest risk at 1.06 or below [83]. Recently, other authors have shown that calculating the ratios of TChol/HDL-C [83,84,85], TG/HDL-C [83,86,87], and LDL-C/HDL-C [88] may provide useful assessments of the risk of heart disease. A common observation in these studies is that an increase in HDL-C relative to the other lipid parameters is associated with a lower risk of heart disease. Table 10 shows these ratios calculated using the data from Table 3. An analysis of the ratios for the studies in this review as shown in Table 10 suggests that CNO lowers the risk of heart disease.

### 4.7. Total Cholesterol, LDL-Cholesterol, and Triglyceride Values Did Not Change Significantly over Time for Coconut Oil or Sunflower Oil in a Two-Year Study of People with Cardiovascular Disease

To reduce the purported risk of heart disease, the AHA and the DGA have long cautioned to avoid consuming foods such as full-fat dairy, eggs, fatty meats, and tropical oils and have encouraged replacing saturated fat with polyunsaturated oils based on the assumption that saturated fat increases LDL-C and polyunsaturated oils decrease LDL-C. However, the following study raises doubts about whether such changes are sustained in the long term. The Vijayakumar (2016) [36] prospective study provided three groups of results in a two-year study of 190 people with diagnosed cardiovascular disease who were all taking statins and were assigned to use either CNO or SunO (sunflower oil), a MUFA oil, for food preparation at home. The oils were provided to the participants and their families to use as their cooking oil, and the amount of oil to be consumed by the participants was calculated at 15% of the total daily calories, which would equate to 33 to 40 gm/day on a 2000–2400 kcal diet. Table 11 compares the differences in lipid values for people consuming CNO or SunO at 3 months, 1 year, and 2 years. The authors did not report *p*-values for the results within each test oil study group but found no statistically significant differences in the lipid values at any time point between the CNO and SunO groups. There were also minimal changes in VLDL of less than ±1 mg/dL at all time points for both groups.

The SDs reported by Vijayakumar [36] were large for each lipid value for both the CNO and SunO groups at baseline and at all other time points, ranging from ±28 to ±44 mg/dL for TChol, ±20 to ±29 mg/dL for LDL-C, ±9 to ±16 mg/dL for HDL-C, and ±37 to ±64 mg/dL for TG. For example, the SDs indicate that the TChol values in the CNO group ranged from 120 to 180 mg/dL at baseline, 114 to 181 mg/dL at three months, and 120 to 178 mg/dL at two years. Such wide ranges in the lipid values illustrate the marked inter-individual differences that can occur within populations consuming the same cooking oil as a staple. It is noteworthy that the baseline and final values were well below the “at risk” cutoff of 200 mg/dL for TChol for these high-risk individuals throughout the entire two-year period for the CNO and SunO groups. The lack of significant differences between the CNO and SunO groups at all time points also illustrates that factors other than fat in the diet likely play a much more important role in an individual’s lipid values and cardiac health.

Vijayakumar (2016) [36] also studied other biomarkers of cardiac risk and found no differences between the CNO and SunO groups in the body mass index, percentage of body fat, waist/hip ratio, antioxidant levels, flow-mediated vasodilation, lipoprotein a, apolipoprotein B, apolipoprotein A, ultrasensitive C-reactive protein, glycosylated hemoglobin, or five metabolically important antioxidants. Two people died from vehicular accidents, but there were no other deaths in either group. Two people in each group required revascularization procedures (stents) during the two-year period. This study is particularly important since it looked at the long-term effects of consuming CNO, a highly saturated fat, or SunO, a MUFA oil, in the diet in older people, who are at the greatest risk for serious cardiovascular events. This study illustrates the likelihood that changes in TChol and LDL-C values may be transient, since neither group had a difference from baseline of >±5% at 3 months or 1 year, and only the differences in HDL-C at 2 years were >5% higher for both groups.

In summary, this analysis shows that adding CNO to the diet increases HDL-C in most individuals, along with improvements in the lipid ratios. However, for a given individual, adding CNO to the diet could result in an increase, decrease, or no change in lipid values, and any change in TChol and LDL-C may be temporary. When adding CNO to the diet, it would be reasonable to periodically monitor individuals for whom hypercholesterolemia or other cardiac risk factors are of concern, while recognizing that any changes in the lipid profile values in the early weeks may be transient.

## 5. Discussion

### 5.1. Coconut Oil Raises HDL-Cholesterol and Improves Lipid Ratios in Short-, Medium-, and Long-Duration Studies

This analysis includes 29 studies with 984 data sets of baseline and end-of-test-period lipid values for 792 distinct individuals while consuming CNO. The main observations are summarized as follows.

- For all studies combined, the average differences for TChol, LDL-C, and TG were below the baseline. For long-duration studies, the average differences for TChol and TG were below the baseline, and LDL-C was less than 1% above the baseline. For medium-duration studies, the average differences for TChol, LDL-C, and TG were below the baseline. For short-duration studies, the average difference for TChol was less than 1% above the baseline, and those for LDL-C and TG were below the baseline. Therefore, the average difference for TChol was slightly above the baseline only for studies lasting 3 to 7 weeks, which was likely due to higher HDL-C, since the average difference for LDL-C was below the baseline.
- The HDL-C values were above the baseline values for the combined studies, as well as for all study durations.
- The lipid ratios TChol/HDL-C, LDL-C/HDL-C, and TG/HDL-C were improved in all groups combined, as well as for all study durations.
- The large SDs for the differences in all lipid values reported by the authors in most studies suggest that there is marked individual variability in the response to introducing CNO into the diet, highlighting the complexity of cholesterol metabolism.

When the results of each group consuming CNO or another test oil are considered separately, it is apparent that the average values for differences in TChol, LDL-C, HDL-C, and TG sometimes increased, decreased, or were essentially unchanged. However, according to the saturated fat paradigm, adding saturated fat to the diet should consistently increase TChol and LDL-C, and replacing saturated fat with PUFAs should lower TChol and LDL-C. However, in these same studies, the average differences in the TChol and LDL-C values for groups taking a comparator PUFA or MUFA oil also varied, sometimes increasing, decreasing, or showing no significant difference, with equally large SDs. Fats that are high in saturated LCFAs, such as beef tallow [52] and palm/palm olein oil [53,64,70], decreased TChol and LDL-C or increased TChol and LDL-C by <5% [48], contrary to the predicted effects based on their FA compositions. In addition, CNO sometimes performed better in its effects on lipid values than a comparator PUFA oil. For example, in a study by Assunção (2009) [47], two groups of 20 women each were assigned to receive CNO or soybean oil (SBO) for 12 weeks, with otherwise identical dietary and physical activity instructions. The SBO group reported much higher, statistically significant increases in TChol of +19.8 mg/dL (+10.4%) and LDL-C of +25.5 mg/dL (+23.5%), while, for the CNO group, TChol increased by +5.6 mg/dL (+2.9%) and LDL-C increased by +3.9 mg/dL (+3.5%), which were not statistically significant. In the weight loss study by Oliveira-de-Lira (2018) [45], LDL-C decreased in all four oil test groups, with just a 0.48 mg/dL difference in the LDL-C levels between CNO, SBO, and safflower oil.

### 5.2. Strengths and Limitations

The primary strength of this analysis, compared to previous reviews, is the large number of lipid profile data sets from globally diverse populations, including Asian, Asia-Pacific, European, Middle Eastern, North American, and South American populations, where the habitual diets differ from country to country. The subjects consumed CNO in prospective studies with durations of 3 weeks to 2 years, which also enabled an assessment of whether the lipid response is sustained over time. The only long-duration study of 96 individuals provided lipid profile data sets at 3 months, 1 year, and 2 years [36]. The results of the long-duration study are corroborated by the Sabitha (2009) and Palazhy (2018) population studies of long-term CNO and SunO consumers, which were discussed earlier [55,56].

The most serious limitations of this analysis are the relatively short duration of most studies and the heterogeneity of the studies included in this review. These limitations also apply to the other reviews discussed in the Introduction that compared CNO to other dietary fats and oils. Except for the Vijayakumar (2016) two-year study [36], the studies cannot provide associations between changes in lipid profiles and cardiovascular or any other important long-term health outcomes, mainly because the short study durations, precluding this type of analysis. The studies in this review were conducted over a nearly 40-year span, and the diets in the different regions may have changed substantially during this timeframe. Another limitation is that the raw data for the individual lipid profile values and results were not available for any of the studies, but the large SDs found in most studies suggest that there was marked variability between the individuals within the study groups. In addition, the average baseline values were much higher and the range wider in some groups than in other groups, which is to be expected because of the heterogeneity in the people recruited to participate in these dietary oil studies (see Table 4 and Table 5). The heterogeneity included the following: differences in sex—only men, only women, or mixed; differences in health conditions—young healthy subjects with a normal BMI, young obese people, older people with or without a health condition, such as obesity, dyslipidemia, metabolic syndrome, diabetes, diagnosed coronary artery disease, or Alzheimer’s; and participants either taking or not taking a statin. Some studies included one or more interventions in addition to consuming CNO, such as dietary instruction and exercise; dietary instruction and a statin; a weight loss diet; and a hypercaloric diet with additional calories from muffins, rolls, or cookies containing the test oil. Some studies provided all the food, strictly calculated and sometimes eaten under direct supervision, while other studies sent the oils to the participants’ homes to use in food preparation, with no other dietary guidelines or restrictions. The amounts of the fat or oil varied from 6 g per day in capsules to as much as 117 g per day based on a percentage of the total calories consumed by the participants. Many were crossover studies, some with and some without a washout period and with or without a new baseline at the beginning of each test oil period. Some crossover studies used only the baseline values at entry for the calculations of each test oil period and did not separate the results based on the order of the test oils.

#### 5.2.1. Similar Limitations Are a Problem for Other Reviews and Meta-Analyses

In a 2017 advisory on dietary fat and heart risk, the AHA recommended avoiding CNO based exclusively on its effect on LDL-C in comparison to other oils or when C12:0 is substituted for sugar [89]. This recommendation was based on only seven selected studies in which CNO increased LDL-C in comparison to other oils or fats and did not report the results of studies in which CNO decreased LDL-C, along with a meta-analysis in which most studies lasted 2 to 6 weeks and included many brief liquid diet studies. While liquid diet studies in people provide valuable information, these studies are especially relevant to individuals consuming liquid diets for therapeutic reasons, such as post-surgery or malabsorption syndromes, and for use in infant formulas or nutritional shakes. The results of liquid diet studies may be less predictive of the lipid profile responses of people consuming everyday, highly variable, complex diets, which include many other food components that could affect the lipid parameters and are likewise influenced by genetic and many other epigenetic factors that are unique to a given individual. C12:0 averaged just 1.2% of the total energy and was not included in some studies. The referenced meta-analysis by Mensink (2016) [90] found that substituting C12:0 for carbohydrates minimally increased LDL-C by +0.66 mg/dL per 1% of total energy consumed but also increased HDL-C by +0.73 mg/dL, decreased TG by −0.13 mg/dL, and lowered the ratios of TChol/HDL-C, LDL-C/HDL-C, and TG/HDL-C more than the other fatty acids studied. These results were statistically significant, but these favorable findings for C12:0 were ignored in the 2017 AHA presidential advisory.

The AHA advisory [89] and a recent letter to the editor by members of the AHA [91] argue that it is important to consider what replacement should be used for saturated fat. The letter cites a review by Clark et al. (1997) [92] of 395 metabolic ward studies averaging a 1-month duration that also included liquid formula diets and mentioned that some studies included *trans*-fats. Clarke (1997) noted that replacing saturated fat with MUFAs and PUFAs or complex carbohydrates had the effect of lowering TChol and LDL-C but did not suggest that this had an impact on adverse cardiac events. Clarke (1997) also acknowledged that other reviews had not been able to conclude that the replacement of saturated fat with PUFA oils had a significant impact on LDL-C or HDL-C. Our analysis suggests that brief studies cannot be conclusive, since such studies do not predict cardiovascular events. Likewise, studies of liquid formula diets are most relevant to people consuming liquid diets for therapeutic reasons and perhaps less relevant to people who consume complex diets composed of a wide variety of solid foods with different textures, which can affect the digestion, absorption, and metabolic fates of oils and fats. The studies reported in our analysis illustrate that CNO can be added to the diet in many different ways, and the amount consumed can vary greatly, as well as whether the oil is taken once daily or taken in portions. When adding a new oil to the diet, unless prescribed for therapeutic reasons, such as epilepsy or Alzheimer’s, CNO is more often used in preparing meals, rather than taken from a spoon or in a capsule. How CNO is used in the everyday diet and what other foods the oil is consumed with can also vary considerably for an individual from day to day.

Our analysis of 26 studies does not meet the rigid criteria for a meta-analysis. Earlier, Duarte et al. (2022) [93] conducted a systematic review and meta-analysis of CNO and the cardiometabolic profile, which included 17 studies overall and seven studies in the meta-analyses for LDL-C, HDL-C, and triglycerides. Their findings agree with our analysis, indicating that CNO did not increase LDL-C but did increase HDL-C on average. They also found that coconut oil did not change the triglyceride levels, whereas we found average decreases in TG levels for all studies combined and for each study duration. In a meta-analysis of six studies, Duarte found that the effect of coconut oil on body weight was not significantly different from that of other comparator oils and fats, which concurred with our findings.

#### 5.2.2. Limitations Due to Calculated Rather than Direct Measurement of LDL-Cholesterol

**Figure 5:**
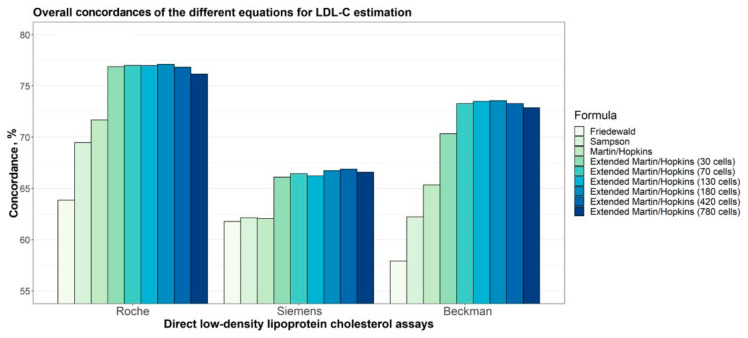
The Zararsiz (2022) study included nearly 89,000 adults and compared the LDL-C results of samples from the same person and measured using a direct colorimetric enzymatic reaction method (Roche, Siemans, or Beckman) versus each of the estimation formulas shown in the legend on the right and represented in the colored bars. None of the formulas showed concordance of more than 77% with a direct method. Of the formulas tested, the Friedewald formula had the lowest % concordance with all three methods of direct measurement, ranging from about 57 to 64%. Reprinted from ref. [94].

**Figure 6:**
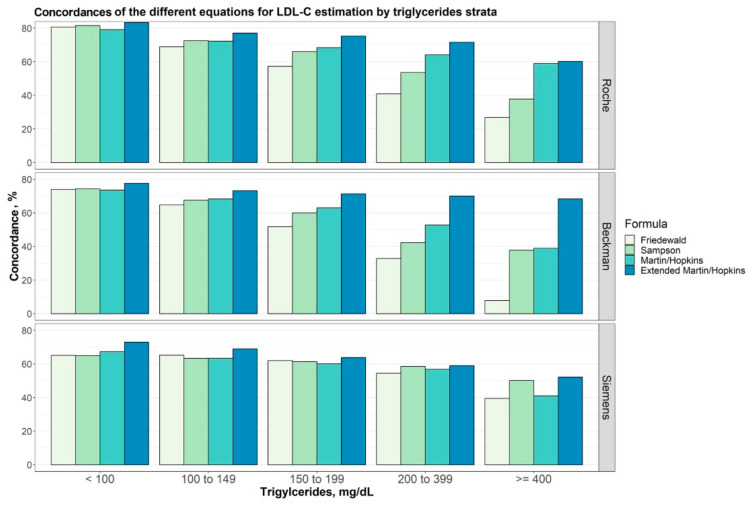
LDL-C results are shown by TG stratum for samples taken from the same person and measured using a direct colorimetric enzymatic reaction method (Roche, Siemans, or Beckman) versus each of the estimation formulas shown in the legend on the right and represented in the colored bars. None of the estimation formulas had more than 81% concordance with a direct method, and the concordance decreased with each higher TG stratum. The Friedewald formula had the lowest concordance with the Roche and Beckman direct methods for each TG stratum of 150 mg/dL or greater and had less than 10% concordance with the Beckman direct method at TG levels ≥ 400 mg/dL. The concordance of the estimation formulas with the Siemans direct method was no greater than 74% at any TG level. Reprinted from ref. [94].

There were differences among the studies in our analysis in how LDL-C was measured. Rather than the direct measurement of LDL-C, most studies, including those that intentionally recruited people with dyslipidemia, estimated LDL-C by using the Friedewald formula, which is known to be less accurate since it assumes an average TG level and the accurate measurement of HDL-C [79]. A study by Zararsiz and co-workers (2018), with 88,943 male and female participants above 18 years old, compared the LDL-C results for samples taken from the same person using one of three direct homogenous colorimetric enzymatic reaction measurements (Roche, Siemens, and Beckman) versus calculation methods using estimation formulas (Friedewald, Sampson, Martin/Hopkins, and extended versions of Martin/Hopkins). The measurements using the estimation formulas could be discordant by virtue of being higher or lower than the direct measurement. Each of the calculation methods showed poor concordance with all three methods of direct measurement (about 57 to 77% depending on the direct method). In addition, there were marked differences between the calculation methods for samples taken from the same person, with the Friedewald formula presenting the greatest disparity (about 57 to 64% concordance) (see Figure 5). When the results were differentiated according to the triglyceride levels, there was significantly reduced concordance (none greater than 81%) between the direct and calculated methods of LDL-C measurement, even at TG levels of less than 100 mg/dL. Likewise, calculation methods became less concordant with direct methods with increase in the TG levels and was most prominent when TG was greater than 400 mg/dL, with the Friedewald formula showing the poorest concordance with the Beckman direct method at less than 10%. For TG levels between 200 and 399 mg/dL, the Friedewald formula showed only about 35% agreement with the direct measurement of LDL-C using the Beckman direct measurement (see Figure 6) [94]. This is important information, since medical providers often rely upon calculated values of LDL-C to initiate treatment with LDL-C-lowering medications.

### 5.3. How an Oil Is Processed Might Explain the Inconsistent Results Between Similar Studies

The methods used to process the oils may explain some of the inconsistent results between studies with similar designs. The processing methods and fatty acid compositions of the test oils were not reported for many studies. Adding some clarity to the issue of whether the processing method of an oil affects cholesterol metabolism, Arunima et al. (2014) [95] compared supplementation with equal amounts of various oils in rats. Supplementation with VCO showed decreased levels of serum and liver TChol, TG, and phospholipids compared with refined CNO, olive oil, and sunflower oil. Notably, the serum cholesterol level was higher by 29% and the liver cholesterol level was higher by 105% in rats in the RBD CNO group compared to the VCO group. The authors also found that VCO significantly reduced the de novo production of fatty acids and increased the rate of fat catabolism, which was explained by the increased activity of carnitine palmitoyl transferase I, acyl CoA oxidase, and the enzymes involved in mitochondrial beta-oxidation. The increase in fat catabolism was further accomplished by the upregulation of the mRNA expression of PPARα and its target genes involved in fatty acid oxidation. Arunima et al. concluded that the differences in cholesterol levels might be partly explained by the presence of polyphenols in unrefined VCO, which is produced from fresh coconut meat, compared to RBD CNO, which is produced from copra and is highly processed [95].

In another study, Liu et al. (2019) [96] conducted an in vitro study looking at the effects on human liver cells of RBD CNO at each stage of processing. They found that various contaminants appeared in the oil during each stage, including glycidyl esters and 3-monochloropropandiol (3-MCPD), which is known to induce nephrotoxicity [97]. When the contaminated oils were applied to the liver cell culture, cellular cholesterol and triacylglycerol increased, accompanied by an increase in HMG-CoA reductase, an enzyme involved in cholesterol biosynthesis, and a decrease in CYP7A1, a gene that controls the degradation of cholesterol. These effects may raise the level of serum TChol. These changes occurred at each step in the processing of the oil (refining/deacidification, bleaching, and deodorization). In addition, phytosterols and polyphenols decreased with each step, ending at about 50% of the original content. When the contaminants were then added to VCO, there were identical effects in the liver cells, suggesting that the contaminants were drivers of this phenomenon. VCO that was not subjected to this processing did not increase cellular cholesterol or triacylglycerol [96].

### 5.4. Large Standard Deviations Question the Applicability of the p-Value to the Real World

Meta-analyses and other reviews, including this one, have combined data from many different individuals to arrive at averages, and some have applied “weighting” that favors small, short studies, as well as other statistical techniques, to arrive at their conclusions. In addition, some meta-analyses and reviews did not report actual values from baseline to the end-of-test period for each oil but reported only differences in the effect on lipid values between two or more oils within a study. Combining the results of studies of dietary oils in many different populations of different age ranges, under many different conditions and often with confounding factors, does not allow for the prediction of lipid values for an individual who consumes a new oil. Our analysis gave equal weight to each lipid profile data set. The SDs for the differences in lipid values and ranges of baseline and final lipid values in many of the 29 groups were large, suggesting very substantial inter-individual variability (see Table 7 and Table 8). Even though this analysis focused on studies of CNO consumption, there was also considerable variation in the response and large SDs for the responses to the comparator oils. For example, two studies comparing CNO to SBO reported widely disparate responses in LDL-C; a study by Mendis and Kumarasunderam (1990) [65] of 25 people consuming 55 mL SBO daily for 8 weeks, with an average baseline LDL-C of 114 mg/dL, reported an average decrease in LDL-C of −26.3 ± 13.92 mg/dL, whereas the study by Assunção et al. (2009) [47] of 20 people consuming 30 mL SBO daily for 12 weeks, with an average baseline LDL-C of 108 mg/dL, found an increase in LDL-C of +25.5 ± 28.7 mg/dL. In addition, other studies have reported that there is considerable variability within individuals in LDL-C values from day to day (6.1%), week to week (6.2%), and month to month (9.5%) [98,99]. Such large SDs within studies, disparities between similar studies, and intra-individual variability question the appropriateness of considering a 5 to 10% difference statistically significant in a collection of highly heterogenous studies to arrive at population-wide guidelines as recommended in the DGA, which has been adopted globally. Such results are even less reliable in clinical settings, where healthcare providers and dietitians aim to offer “evidence-based” guidance on dietary oil consumption to individuals, particularly those at risk for heart disease.

### 5.5. Cardiac Risk Factors Beyond the Lipid Profile

It is misleading to declare that “coconut oil increases total cholesterol and LDL-C” and that “PUFA oils lower total cholesterol and LDL-C” when there is so much variability in the data. Our analysis suggests that increases or decreases in lipid values may be transient and return to baseline as the individual’s metabolism adjusts to the change in diet. As mentioned previously, the recommendation to avoid CNO in the 2017 AHA presidential advisory on dietary fat was based entirely on its purported effect of increasing LDL-C while ignoring the increase in HDL-C and reductions in TG [89]. Cholesterol-lowering therapy is often initiated based solely on the LDL-C level, despite the unreliability of measurement, the lack of concordance of the calculated Friedewald formula values with direct measurement, and the tendency for the LDL-C levels in individuals to fluctuate significantly [100].

The role of LDL-C in cardiovascular disease is complex, and many peer-reviewed published articles have challenged the assertion that LDL-C predicts the CVD risk in the general population. Investigators have reported that more than half of people who have heart attacks have normal LDL-C levels. A study by Sachdeva et al. (2009) from the National Cholesterol Education Program (NCEP) looked at lipid values at the time of hospitalization for 136,905 people admitted to 541 hospitals with confirmed diagnoses of coronary artery disease, including acute coronary syndromes, CAD requiring a revascularization procedure, or other CAD diagnoses unrelated to heart failure. The mean admission LDL-C level was 104.9 mg/dL, with 75% below 130 mg/dL and 17.5% below 70 mg/dL, while 54.6% had abnormally low admission HDL-C levels of <40 mg/dL. In addition, 21.1% of the patients were taking lipid-lowering agents before admission [101].

LDL-C appears to be a less important risk for people with normal blood glucose levels but is an important risk factor for people who also have chronically elevated blood glucose levels and/or inflammation, which may result in the alteration of natural LDL-C to oxidized LDL-C, glycated LDL-C, and small dense LDL-C particles [102,103,104]. Also, an assessment of altered LDL-C and particle size patterns may be more predictive of the cardiovascular risk than the total LDL-C. The total LDL-C and its components may be most meaningful in people who have prior heart conditions or other strong risk factors for heart disease, such as obesity, diabetes, or a family history of early cardiac death. In addition, nearly all the studies in our analysis used a calculated LDL-C, which does not correlate well with the direct measurement of LDL-C, as explained in the discussion of the Zararsiz 2021 article [94]. The equations used in these calculations include the direct measurement of total cholesterol, HDL-C, and triglycerides, and LDL-C is not measured directly but rather is estimated. Therefore, a calculated estimate of LDL-C is highly unreliable and, while not unimportant, the total LDL-C alone should not be used as the sole criterion to determine a treatment plan related to cardiovascular risk. The lipid profile is just one tool available for the evaluation of cardiovascular health. The American College of Cardiology recommends the consideration of additional factors beyond LDL-C to evaluate an individual’s cardiac risk and determine a plan of treatment. The evaluation first identifies people at higher risk using the Atherosclerotic Cardiovascular Disease (ASCVD) 10-Year Risk Estimator for further evaluation, including the consideration of other “risk-enhancing factors” before initiating a statin or other therapy. Risk-enhancing factors include a family history of premature ASCVD (<age 55 for men, and <65 for women), metabolic syndrome/diabetes, certain complications of diabetes, chronic kidney disease, chronic inflammatory conditions, a history of premature menopause (<age 40), a high-risk race or ethnicity, persistently elevated primary hypertriglyceridemia (≥175 mg/dL non-fasting), LDL-C 160–189 mg/dL, elevated high-sensitivity C-reactive protein (≥2.0 mg/L), elevated lipoprotein (a) (>50 mg/dL), elevated apolipoprotein B (ApoB; >130 mg/dL), especially with TG ≥200 mg/dL, and Ankle–Brachial Index (ABI) (<0.9), a ratio of the highest systolic blood pressures in the ankle to the upper arm, or the selective use of a coronary artery calcium measurement [105]. Thus, there are many factors beyond the TChol and LDL-C levels that determine overall cardiac health.

In this analysis, 9 of 26 studies reported changes in apolipoprotein A (ApoA) and ApoB levels, which are considered useful indicators of cardiac health risks. However, six of these studies looked for statistical significance only between the test oil groups and not from the baseline to the final values in the CNO group, and they found no significant differences in the ApoA or ApoB values between the CNO groups and comparator oils [48,53,54,69,70,71]. Two studies found significantly higher (improved) ApoA values for the CNO group compared to the other oils [63,72], and only one study looked at whether the change from the baseline to final values for the CNO group was significant and found a significant increase in ApoA and a slight increase in ApoB from baseline for the CNO group. The increase in ApoA was also statistically significant for the CNO group compared to the diet-only group [66]. One study did not report ApoA or ApoB results but reported that the ratio of ApoB to ApoA was not significantly different between the CNO and sunflower test oil groups [36].

While the status of certain endogenous antioxidants, such as glutathione, glutathione peroxidase, and superoxide dismutase, and vitamin C is also considered important to cardiac health, only two studies measured the effects of CNO and other oils on antioxidant levels. One study found substantial increases in alpha- and beta-carotene levels from baseline for the CNO group but much larger increases for red palm oil [70]. Another study measured the levels of antioxidants and did not find statistically significant differences between the CNO and SunO groups at any time point, although there was a steady upward non-significant trend for lipid peroxidase, glutathione reductase, and glutathione S transferase in the CNO group at 3 months, 1 year, and 2 years [36].

Just one group studied the effect of CNO on a measure of endothelial function, specifically flow-mediated vasodilation, and did not find a significant difference at 3 months, 1 year, or 2 years between the CNO and SunO groups [36].

The study by Vijayakumar et al. [36] was particularly comprehensive in its approach to studying the effects of CNO and SunO on parameters related to overall heart health. It would be helpful for future investigations to evaluate the impact of CNO, other edible oils and fats, and specific fatty acids on general, metabolic, and cardiovascular health in a more holistic manner by exploring the effects on lipid parameters beyond the standard lipid profile. Such parameters might include the evaluation of ApoA, ApoB, Lp(a), oxidized and glycated LDL-C, LDL-C particle sizes and patterns, and the effects on endothelial function, antioxidant systems, and other metabolic pathways.

Obesity and abdominal adiposity are known risk factors for heart disease. Only 2 of the 26 studies did not report the effects of the dietary interventions on body weight or other anthropometric measurements [48,68]. Of the remaining 24 studies, 19 studies reported no significant difference in body weight and/or BMI for any group in the study. Three studies reported significant improvements in several anthropometric measurements: Oliveira-de-Lira et al. (2018) [45] performed a weight loss study in women, which included groups taking coconut, chia, safflower, or soybean oils, and reported that coconut oil had the greatest effects on anthropometric measurements, which were significant—in particular, a reduction in body weight, BMI, waist circumference, waist-to-height ratio, the conicity index (an assessment of central adiposity), and % body fat and an increase in % lean body mass. Cardoso et al. (2015) [66] conducted a study of people with coronary artery disease, in which all participants were placed on a special diet for three months, followed by the replacement of fats in the diet with coconut oil in one group versus the continuation of the special diet in the control group. They reported that both groups had significant decreases in body weight and BMI and a decrease in weight circumference favoring the coconut oil group, with no significant differences between the groups. A third study by Assuncao et al. (2009) [47] reported that both the coconut oil and soybean oil groups had significant decreases in body weight and BMI, but only the coconut oil group had a significant decrease in waist circumference. In addition, four studies reported improvements in a single anthropometric measurement. Korrapati et al. (2019) [54] found no difference in body weight for either group but reported that the coconut oil group experienced a significant increase in fat-free mass and improved insulin sensitivity compared to the group taking peanut oil. Setyawati et al. (2023) [76] and Vogel et al. (2020) [74] each reported non-significant decreases in BMI in the coconut oil groups. Harris et al. (2017) [50] reported a significant increase in % lean mass. Setyawati (2023) [76] also reported decreased caloric intake for the coconut oil group. Only two studies that were both described in the same article by McKenney et al. (1995) [46] reported weight gain in both the coconut oil and canola groups. However, the oils were incorporated into muffins containing other ingredients that added more than 600 kcal to the baseline diets, and the participants in all groups gained an average of five pounds (about 2.25 kg). In summary, these studies show that incorporating coconut oil into a given diet does not lead to excess calorie intake and weight gain.

### 5.6. Impact of Individual MCFAs on Lipid Parameters in Humans

Because MCFAs are also consumed or tested in different formulations—for example, as MCT oil—their effects are assumed to be applicable to CNO, which should not be the case. This section focuses on the impact of particular MCFAs on lipid parameters in clinical studies and not on CNO itself, which is discussed above.

Studies on the impact of MCFAs (C6:0, C8:0, C10:0, and C12:0) taken individually on the lipid parameters in humans are limited and mostly short-term. A 3-week clinical study on an MCT mixture of C8:0 (65%) and C10:0 (35%), involving 18 men, reported an 11% increase in plasma TChol, a 12% increase in LDL-C, and a 22% increase in TG, but the HDL-C level was unchanged [106]. A meta-analysis of seven articles on the effects of a mixture of C6:0, C8:0, and C10:0 fatty acids on blood lipids in clinical trials lasting at least 2 weeks concluded that these MCFAs had insignificant effects on TChol, LDL-C, and HDL-C, but caused a small increase in TGs [107]. 

Mensink et al. (2003) [108] sought to evaluate the effects of individual fatty acids on the TChol level and the TChol/HDL-C ratio by conducting a meta-analysis of 60 controlled short-term clinical trials, many of which used liquid diets. He reported that C12:0 increased TChol but that much of this effect was from the increase in HDL-C and that this effect decreased the TChol/HDL-C ratio. Mensink later updated this to 84 clinical studies that were conducted between 2009 and 2013. The following changes in the lipid ratios were reported: TChol/HDL-C (−0.035), LDL-C/HDL-C (−0.024), TG/HDL-C (−0.024). This was reported as a WHO publication [90].

Numerous beneficial properties of CNO have been reported. Besides the effects on lipid parameters, CNO, which is predominantly composed of MCFAs, is ketogenic regardless of what else is consumed at the same time and can be used directly as fuel in certain tissues, including the brain, which may be beneficial for the treatment of Alzheimer’s disease (AD) [9,109]. In a 21-day clinical trial that assessed cognitive improvement in 44 AD patients using the Mini-Mental Sate Examination (MMSE), CNO appears to have improved cognitive abilities, which varied depending on the cognitive area [110]. In a 24-week randomized, placebo-controlled clinical study involving 120 mild-to-moderate AD patients > 65 years old, VCO was shown to improve the MMSE scores in people who were apolipoprotein E4-positive, while also reducing the TChol and LDL-C levels [68]. A recent systematic review and meta-analysis by Bafail et al. (2024) that included seven studies of the cognitive effects of adding CNO to the diets of people with Alzheimer’s disease reported that “all studies showed consistent results regarding the effects of CNO on cognitive scores, with little variability in the true effects of CNO on cognitive scores across the studies included in the meta-analysis”. The authors further concluded that “CNO improved cognitive scores in patients with AD compared with those in the control group (*p* < 0.05). The results of this study add to the increasing amount of evidence indicating that MCTs found in CO might be a way to improve abilities and potentially slow the advancement of AD” [111].

MCFAs are found in human milk, predominantly as C12:0 [112], which is antimicrobial [113]. The focus on cardiac risk has long overshadowed the significant nutritional and therapeutic benefits of MCFAs and CNO.

## 6. Summary and Conclusions

In this paper, we reviewed the literature on CNO and performed an analysis of 26 studies on the effects of CNO consumption conducted over the past forty years, including an analysis of 984 lipid profile data sets. This represents the largest analysis of CNO to date and the first to focus on the absolute lipid responses of subjects to CNO, instead of comparative responses to other oils and fats. The main findings of this work are as follows.

1. Coconut oil has the highest amount of MCFAs (C6:0 to C12:0) among all the common dietary oils and fats, whereas the other commonly used oils and fats are mainly LCFAs, both saturated and unsaturated (>C14). CNO is unique in that it is predominantly lauric acid (C12:0). Coconut oil is sui generis.
2. MCFAs, particularly C12:0, have different metabolic properties compared to saturated LCFAs, such as C16:0. The high intake of C16:0 may cause hepatic inflammation when consumed in large amounts; this is not observed with C12:0. Studies using lard, palm oil, and C16:0 as comparators are not applicable to CNO and C12:0.
3. Measurements of lipid parameters with CNO intake appear to depend on the duration of the study. Analyses of studies on CNO show that there is no consistency in the levels of TChol and LDL, while the TG levels tend to decrease and HDL-C levels tend to increase regardless of the duration.
4. The large SDs and ranges of the results reported in most studies indicate that there is marked variability in the lipid profile responses of individuals to adding a new oil to the diet. In addition, there would be considerable variability in what food item or macronutrient CNO would replace from person to person and meal to meal. Therefore, even though the average results suggest that CNO increases HDL-C and does not increase LDL-C, for example, these results cannot predict the effect of CNO on the HDL-C or LDL-C levels in any given individual, which could vary widely, especially during the first few weeks to months. When there is concern, monitoring the lipid profile periodically would be reasonable.
5. There is no evidence from interventional studies that consuming CNO increases the incidence of adverse fatal or nonfatal cardiovascular events. Likewise, studies of populations consuming CNO and the coconut diet as a staple have found no evidence of an increase in the incidence of CVD.
6. Unlike many other studies that draw conclusions based on comparisons between CNO and PUFA oils based on TChol and LDL-C only, this study shows that CNO has a strong tendency to raise HDL-C regardless of the study duration, and that the calculation of the lipid ratios—TChol/HDL-C, LDL-C/HDL-C, and TG/HDL-C—gives healthy ratios and indicates that CNO is a heart-healthy oil. Most importantly, there are no studies that show that CNO increases the incidence of cardiovascular disease.
7. The conclusions of studies on the health effects of “saturated fat”, including animal studies and clinical trials, should specify which fat or oil or which specific saturated fatty acids were studied. The results of studies that use lard to represent saturated fat should be reassessed.

This study concludes that the dietary recommendation to avoid consuming CNO due to its effects on lipid parameters is not justified.

## Figures and Tables

**Table 1:** Fatty acid and cholesterol content of coconut oil and other selected plant oils and animal fats, which are discussed in this paper. The data are presented as wt./100 gm. The predominant fatty acid in each oil or fat is highlighted in yellow, and the characteristic group is highlighted in green. Note that lard, which has been classified as a saturated fat for a century, is higher in MUFAs than SFAs. (Data are from the USDA FoodData Central [11], except those for safflower oil, for which we also use data from CODEX STAN 210-1999 [12]). CNO = coconut oil; SaffO = safflower oil; SBO = soybean oil; SunO = sunflower oil; FA = fatty acid; satd = saturated.

Constituent	Plant Oil							Animal Fat		
	CNO	Corn	Olive	Palm	SaffO *	SBO	SunO	Lard	Tallow	Butter
Fatty Acid	gm/100 gm									
C6:0 Caproic acid	0.5	-	0.0	0.0	ND	-	0.0	0.0	0.0	2.0
C8:0 Caprylic acid	6.8	-	0.0	0.0	ND	-	0.0	0.0	0.0	1.2
C10:0 Capric acid	5.4	-	0.0	0.0	ND	-	0.0	0.1	0.0	2.5
C12:0 Lauric acid	41.8	-	0.0	0.1	ND	-	0.0	0.2	0.9	2.6
C14:0 Myristic acid	16.7	0.0	0.0	1.0	0.09	0.1	0.0	1.3	3.7	7.4
C16:0 Palmitic acid	8.6	11.1	11.3	43.5	6.1	10.3	4.5	23.8	24.9	21.7
C18:0 Stearic acid	2.5	1.6	2.0	4.3	2.3	3.7	3.0	13.5	18.9	10.0
C18:1 Oleic acid	6.3	27.2	71.3	36.6	13.6	21.4	62.9	41.2	36.0	20.0
C18:2 Linoleic acid	1.7	51.9	9.8	9.1	69.1	50.9	20.6	10.2	3.1	2.7
C18:3 Linolenic acid	0.02	1.0	0.8	0.2	0.5	6.6	0.2	1.0	0.6	0.3
MCFA	54.5	0.0	0.0	0.1	0.0	0.0	0.0	0.3	0.9	8.3
LCFA (satd)	27.8	12.7	13.3	48.8	8.5	14.1	7.5	38.6	47.5	39.1
MUFA	6.3	27.2	71.3	36.6	13.6	21.4	62.9	41.2	36.0	20.0
PUFA	1.7	52.9	10.5	9.3	69.6	57.5	20.8	11.2	3.7	3.0
Total FA	90.3	92.9	95.1	94.8	91.5	93.0	91.2	91.3	88.1	70.4
	mg/100 gm									
Cholesterol	0	0	0	0	0	0	0	95	109	215

* SaffO (safflower oil) is estimated as gm/100 gm. The fatty acid values assume that the total fatty acids in SaffO are 91.5 gm/100 gm; the % composition is from CODEX STAN 210-1999.

**Table 2:** Comparison of lipid profiles in healthy people and people with diabetes in Kerala, India, consuming either CNO or SunO for at least six years [55]; mg/dL = milligrams per deciliter; *n* = number of subjects.

Group	*n*=	TChol mg/dL	LDL-C mg/dL	HDL-C mg/dL	TG mg/dL
CNO, healthy	35	161.3 ± 30.7	78.3 ± 24.2	47.8 ± 10.0	136.5 ± 44.0
SunO, healthy	35	157.1 ± 28.0	82.6 ± 26.9	44.3 ± 8.5	125.2 ± 38.3
CNO, diabetes	35	172.4 ± 35.9	108.2 ± 35.4	43.8 ± 10.3	162.1 ± 47.9
SunO, diabetes	35	179.1 ± 32.2	121.7 ± 34.9	39.9 ± 9.8	151.2 ± 37.4

**Table 3:** Comparison of lipid profiles in men in Kerala, India, with coronary artery disease, taking cholesterol-lowering drugs, and consuming either CNO or SunO for at least two years [56]; mg/dL = milligrams per deciliter; *n* = number of subjects.

Test Oil	*n* =	TChol mg/dL	LDL-C mg/dL	HDL-C mg/dL	TG mg/dL
CNO	73	148.2 ± 36.5	96.7 ± 30.6	41.3 ± 18.9	119.2 ± 49.5
SunO	80	147.2 ± 26.7	93.1 ± 22.1	37.1 ± 9.8	107.8 ± 44.1

**Table 4:** List of the 29 groups of lipid profile data sets in 26 coconut oil studies grouped according to duration of the study. CNO = coconut oil; VCO = virgin coconut oil; gm = grams; mL = milliliters; # = number of.

Duration	# Data Groups	# Data Sets	Study	Comments
Long 1–2 years	2	192	1. Vijayakumar 2016 [36], 1 year 2. Vijayakumar 2016 [36], 2 years	India, 2 years, results at 3 months, 1 year, 2 years, n = 96, CNO 15% energy (33–40 gm)
Medium 8–24 weeks	11	442	1. Mendis 1990 [65]	Sri Lanka, 8 weeks, n = 25, CNO 21% of energy (55 gm)
			2. Assunção 2009 [47]	Brazil, 12 weeks, n = 20, CNO 30 mL daily
			3. Cardoso 2015 [66]	Brazil, 3 months, n = 92, VCO 13 mL daily
			4. Vijayakumar 2016 [36], 3 months	India, 2 years, results at 3 months, 1 year, 2 years, n = 96, CNO 15% energy (33–40 gm)
			5. Chinwong 2017 [67]	Thailand, 8 weeks, n = 32, VCO 30 mL daily
			6. Oliveira-de-Lira 2018 [45]	Brazil, 8 weeks, n = 18, weight loss diet, CNO 6 gm daily
			7. Korrapati 2019 [54]	India, 8 weeks, n = 9, CNO 35 gm daily
			8. Fernando 2023 [68]	Sri Lanka, 24 weeks, n = 43, VCO 30 mL daily
			9. Jeyakumar 2023 [69]	India, 8 weeks, n= 22, VCO 35 gm daily
			10. Swarnamali 2024 [64]	Sri Lanka, 8 weeks, n = 37, CNO 10% of energy (17 gm)
			11. Teng 2024 [70]	Malaysia, 12 weeks, n = 48, VCO 53 gm daily
Short 3–7 weeks	16	350	1. Reiser 1985 [52]	USA, 5 weeks, n = 16, CNO 21% of energy (80–117 gm)
			2. Heber 1992 [53]	USA, 3 weeks, n = 9, CNO, CNO 17.5% of energy (41 gm)
			3. Cox 1995 [63], males 4. Cox 1995 [63], females	New Zealand, 6 weeks, CNO 39 gm, results for males (n = 13) and females (n = 15) reported separately
			5. Schwab 1995 [71]	Finland, 4 weeks, n = 15, lauric acid 4% of total energy, CNO varied from 16 to 26 gm daily
			6. McKenney 1995 [46] (Study 1)	USA, 6 weeks, n= 11, CNO 42 gm daily
			7. McKenney 1995 [46] (Study 2)	USA, 6 weeks, n = 17, lovastatin 6 weeks, then CNO 42 gm for 6 more weeks
			8. Lu 1997 [51]	USA, 3 weeks, n = 15, CNO 10% of energy (30 gm)
			9. Cox 1998 [72]	New Zealand, 4 weeks, n = 37, CNO 39 gm daily, male and female results combined
			10. Voon 2011 [48]	Malaysia, 5 weeks, n = 45, CNO 44 gm daily
			11. Khaw 2018 [49]	UK, 4 weeks, n = 28, VCO 50 mL daily
			12. Harris 2017 [50]	USA, 4 weeks, n = 12, VCO 30 mL daily
			13. Maki 2018 [73]	USA, 4 weeks, n = 12, CNO 54 gm daily
			14. Vogel 2020 [74]	Brazil, 45 days, n= 15, VCO 12 mL daily
			15. Nikooei 2021 [75]	Iran, 4 weeks, n = 22, VCO 30 mL daily
			16. Setyawati 2023 [76]	Indonesia, 30 days, n = 68, VCO 1.2 mL/kg/day

**Table 5:** Changes in lipid profiles in people consuming CNO for 3 weeks to 2 years (* Vijayakumar (2016) [36] contributed three groups of data sets for each of the 96 participants); mg/dL = milligrams per deciliter.

Duration	# Data Groups	# Data Sets	Average	TChol	LDL-C	HDL-C	TG
All groups combined, 3 weeks to 2 years	29	984 *	Baseline (mg/dL)	186.2	117.7	45.4	125.3
			Final (mg/dL)	185.9	115.5	48.1	121.7
			Difference (mg/dL)	−0.3	−2.2	2.6	−3.6
			Percent (%)	−0.13	−1.8	5.8	−2.9
Long, 1 to 2 years	2	192	Baseline (mg/dL)	149.8	90.3	40.8	115.0
			Final (mg/dL)	146.9	91.0	42.8	110.7
			Difference (mg/dL)	−2.9	0.7	2.0	−4.3
			Percent (%)	−2.0	0.8	4.9	−3.7
Medium, 8 to 24 weeks	11	442	Baseline (mg/dL)	182.8	115.5	44.3	122.8
			Final (mg/dL)	182.2	112.9	45.3	121.2
			Difference (mg/dL)	−0.6	−2.6	1.0	−1.7
			Percent (%)	−0.31	−2.3	2.3	−1.4
Short, 3 to 7 weeks	16	350	Baseline (mg/dL)	210.4	135.5	49.5	134.1
			Final (mg/dL)	212.0	132.3	54.4	128.4
			Difference (mg/dL)	1.6	−3.1	4.9	−5.7
			Percent (%)	0.8	−2.3	10.0	−4.3

**Table 6:** The overall average change in each lipid parameter for the different time periods is tabulated. For TChol, LDL-C, and TG, results that show a decrease or no change are highlighted in green, and results that show an increase are highlighted in yellow. For HDL-C, results that show an increase are highlighted in blue, and results that show a decrease are highlighted in gray. Overall, proportionately more short-duration than medium-duration studies reported increases in TChol and LDL-C, shown in yellow. However, HDL-C generally increased regardless of the duration of the study, as shown in blue, and TG generally decreased, as shown in green. A subgroup of very short studies of 3–4 weeks is indicated by §. Diff = difference; gm = grams; mL = milliliters; *n* = number of subjects; #1 = first study; #2 = second study.

Study Group	Duration	Amount of CNO/Day	*n* =	TChol % Diff	LDL-C % Diff	HDL-C % Diff	TG % Diff
Long duration (1 to 2 years)							
Vijayakumar 2016 [36]	1 year	33–40 gm	96	−3.5	0.8	3.9	−2.6
Vijayakumar 2016 [36]	2 years	33–40 gm	96	−0.4	0.8	5.9	−4.9
Medium duration (8 to 24 weeks)							
Mendis 1990 [65]	8 weeks	55 gm	25	−0.6	−3.7	3.6	2.1
Oliveira-de-Lira 2018 [45]	8 weeks	6 gm	18	−8.1	−10.4	5.0	−24.9
Korrapati 2019 [54]	8 weeks	35 gm	9	0.0	−2.4	10.4	−6.6
Chinwong 2017 [67]	8 weeks	30 mL	32	−1.4	−5.2	6.5	−4.6
Jeyakumar 2023 [69]	8 weeks	35 gm	22	8.1	11.5	−0.3	6.0
Swarnamali 2024 [64]	8 weeks	17 gm	37	4.0	−1.5	1.2	0.9
Assuncao 2009 [47]	12 weeks	30 mL	20	2.9	3.5	7.0	4.0
Teng 2024 [70]	12 weeks	53 gm	48	−9.3	−11.0	−9.1	−0.8
Cardoso 2015 [66]	3 months	13 mL	92	3.3	3.7	8.3	−1.3
Vijayakumar 2016 [36]	3 months	33–40 gm	96	0.9	−1.1	0.0	−3.2
Fernando 2023 [68]	24 weeks	30 mL	43	−3.3	−7.4	4.9	4.1
Short duration (3 to 7 weeks)							
Heber 1992 [53] §	3 weeks	41 gm	9	18.2	24.0	5.0	18.3
Lu 1997 [51] §	3 weeks	30 gm	15	−5.7	−2.6	−6.6	−14.3
Schwab 1995 [71] §	4 weeks	16–26 gm	15	0.4	−1.7	−3.9	−4.4
Cox 1998 [72] §	4 weeks	39 gm	37	−0.6	6.5	10.0	−9.0
Harris 2017 [50] §	4 weeks	30 mL	12	8.3	10.9	10.3	−8.3
Khaw 2018 [49] §	4 weeks	50 mL	28	3.7	−2.6	14.0	7.9
Maki 2018 [73] §	4 weeks	54 gm	12	7.1	4.6	6.5	5.9
Nikooei 2021 [75] §	4 weeks	30 mL	22	15.8	19.5	18.8	−20.5
Setyawati 2023 [76]	30 days	1.2 mL/kg	68	−8.5	−21.2	23.5	−4.8
Reiser 1985 [52]	5 weeks	80–117 gm	16	6.3	14.6	2.2	−2.5
Voon 2011 [48]	5 weeks	44 gm	45	5.1	7.8	11.4	−6.2
Cox 1995 [63], Males	6 weeks	39 gm	13	1.5	2.8	−3.0	13.4
Cox 1995 [63], Females	6 weeks	39 gm	15	1.4	1.3	−2.3	−7.3
McKenney 1995 [46] #1	6 weeks	42 gm	11	4.9	4.3	8.2	2.5
McKenney 1995 [46] #2	6 weeks	42 gm	17	−2.7	−9.2	10.2	7.8
Vogel 2020 [74]	45 days	12 mL	15	−4.8	−10.2	9.3	−1.6

**Table 7:** Summary, for all durations combined, of averages and ranges of baseline values, final values, and differences from baseline to final values (as mg/dL and %), as well as ranges of standard deviations (SDs) for final values among all 29 groups. Large values and SDs are rounded to the nearest whole number; mg/dL = milligrams per deciliter.

	TChol mg/dL	LDL-C mg/dL	HDL-C mg/dL	TG mg/dL
Average baseline value	186	118	45	125
Range of average baseline values	150 to 251	90 to 180	35 to 77	68 to 217
Average final value	185.9	115.5	48.1	122
Range of average final values	145 to 255	88 to 171	35 to 88.2	65 to 231
Average difference	−0.3 (−0.13%)	−2.2 (−1.8%)	2.7 (5.8%)	−3.6 (−2.9%)
Range of differences	−21 to +33 −32% to +16%	−38 to +25 −28% to +21%	−5 to +11 −9.1% to +14%	−44 to +27 −21% to +13%
Range of SDs in final values	±3 to ±54	±4 to ±42	±1 to ±19	±4 to ±115

**Table 8:** This table shows the same data as in Table 7 according to the duration of study, including the ranges of the absolute differences (mg/dL) from baseline for lipid profile values (top), the ranges of percentage differences from baseline (middle), and the ranges of SD values (mg/dL) (bottom) as reported by the authors in 29 groups consuming CNO. Larger values and SDs are rounded to the nearest whole number.

Duration	Unit	TChol	LDL-C	HDL-C	TG
Short 3 to 7 weeks	mg/dL	−21 to +33	−38 to +25	−3. to +11	−44 to +27
	%	−32 to +16	−21 to +21	−6.6 to +14	−21 to +13
	SD	±3 to ±54	±4 to ±37	±1 to ±18	±4 to ±115
Medium 8 to 24 weeks	mg/dL	−19 to +14	−15 to +13	−5.0 to +4.8	−33 to +6.9
	%	−9 to +8.1	−10 to +12	−9.1 to +10.4	−25 to +4
	SD	±6 to ±45	±2 to ±42	±2 to ±12	±12 to ±94
Long 1 to 2 years	mg/dL % SD	−5.3 to −0.6	+0.7 to +0.8	+1.6 to +2.4	−5.6 to −3.0
		−3.5 to −0.4	+0.8 to +0.8%	+3.9 to +5.9	−4.9 to −2.6
		±29 to ±31	±21 to ±22	±10 to ±11	±47 to ±50

**Table 9:** Lipid profile results in two similar 4-week studies totaling 40 people consuming VCO: 50 mL daily in Khaw 2018 [49] and 30 mL daily in Harris 2017 [50]. Baseline values and SDs are rounded to the nearest whole number. Raw data were not available to provide SDs for combined results; mg/dL = milligrams per deciliter; n = number of subjects.

Study, Duration, # Subjects	TChol mg/dL			LDL-C mg/dL			HDL-C mg/dL			TG mg/dL		
	Base	Diff	SD (±)	Base	Diff	SD (±)	Base	Diff	SD (±)	Base	Diff	SD (±)
Harris 2017 [50] 4 weeks n = 12	220	+18.2 +8.3%	24	124	+13.5 +10.9%	27	64	+6.6 +10.3%	18	117	−9.7 −8.3%	81
Khaw 2018 [49] 4 weeks n = 28	228	+8.5 +3.7%	21	135	−3.5 −2.6%	19	77	+10.8 +14.0%	11	79	+6.2 +7.9%	51
Combined n = 40	226	+11.4 +5.0%	-	132	+1.6 +1.2%	-	73	+9.6 +13.0%	-	90	+1.43 +1.6%	-

**Table 10:** Lipid ratios—TChol/HDL-C, LDL-C/HDL-C, and TG/HDL-C—in people consuming CNO in short-, medium-, and long-duration studies, calculated from Table 3. # = number of.

Duration	# Data Groups	# Data Sets		TChol/ HDL-C	LDL-C/ HDL-C	TG/ HDL-C
All groups 3 weeks to 2 years	29	984	Baseline ratio	4.1	2.6	2.8
			Final ratio	3.9	2.4	2.5
			Difference (Baseline − Final)	−0.2	−0.2	−0.3
Long 1 to 2 years	2	192	Baseline ratio	3.7	2.2	2.8
			Final ratio	3.4	2.1	2.5
			Difference (Baseline − Final)	−0.3	−0.1	−0.3
Medium 8 to 24 weeks	11	442	Baseline ratio	4.1	2.6	2.8
			Final ratio	4.0	2.5	2.7
			Difference (Baseline − Final)	−0.1	−0.1	−0.1
Short 3 to 7 weeks	16	350	Baseline ratio	4.3	2.7	2.7
			Final ratio	3.9	2.4	2.4
			Difference (Baseline − Final)	−0.4	−0.3	−0.3

**Table 11:** Comparison of lipid profile results in a two-year study of 190 people consuming CNO (n = 96) or SunO (n = 94). Baseline values and differences are rounded to the nearest whole number (data from Vijayakumar 2016 [36]). Base = baseline; Diff = difference; mg/dL = milligrams per deciliter.

Oil, Time Point	TChol mg/dL			LDL-C mg/dL			HDL-C mg/dL			TG mg/dL (%)		
	Base	Diff	%	Base	Diff	%	Base	Diff	%	Base	Diff	%
Difference between baseline and 3-month values												
CNO	150	1.4	0.9	90	−1.0	−1.1	41	0.02	0.0	115	−3.7	−3.2
SunO	147	−3.4	−2.3	86	−2.0	−2.3	41	−1.2	−2.9	111	−2.3	−2.0
Difference between baseline and 1-year values												
CNO	150	−5.2	−3.5	90	0.7	0.8	41	1.6	3.9	115	−3.0	−2.6
SunO	147	−7.0	−4.8	86	1.5	1.7	41	−0.6	−1.6	111	3.4	3.0
Difference between baseline and 2-year values												
CNO	150	−0.6	−0.4	90	0.8	0.8	41	2.4	5.9	115	−5.6	−4.9
SunO	147	4.8	3.3	86	3.5	4.1	41	3.6	8.9	111	1.0	0.9

## Data Availability

Data set available in File S3 Tables S2–S5.

## Supplementary Materials

The following supporting information can be downloaded at: https://www.mdpi.com/article/10.3390/nu17030514/s1, File S1: Narrative descriptions of twenty-six studies in order of year published; File S2: Lipid profile results for twenty-six studies; Table S1: Differences in lipid profiles with standard deviations as reported in 26 studies of people consuming coconut oil. File S3: Data sets for the analysis of the impact of coconut oil on lipid parameters. Table S2: Total cholesterol data set for analysis of 26 studies of people consuming coconut oil; Table S3: LDL cholesterol data set for analysis of 26 studies of people consuming coconut oil; Table S4: HDL cholesterol data set for analysis of 26 studies of people consuming coconut oil; Table S5: Triglycerides data set for analysis of 26 studies of people consuming coconut oil.
