# Supplementary material for: Analysis of 26 Studies of the Impact of Coconut Oil on Lipid Parameters: Beyond Total and LDL Cholesterol
Source: Nutrients. 2025 Jan 30;17(3):514. doi: 10.3390/nu17030514 (PMC11819987; doi:10.3390/nu17030514)
Supplement: Supplementary file 1 [file nutrients-17-00514-s001.zip › File S3_Tables S2 to S5_Data Sets for Analysis of Impact of Coconut Oil.pdf]

File S3 Tables S2–S5. Data sets for the analysis of the impact of coconut oil on lipid parameters

File S3. Table S2. Total cholesterol data set for analysis of 26 studies of people consuming coconut oil, including baseline and final values, standard deviations (as reported by the investigators), ranges, ranges for baseline and final values, average differences and percent differences. Beneath the table are shown the calculations for average baseline and final total cholesterol values, average differences and percent differences for all studies combined, and for long-, medium-, and short-duration studies. *n* = number of subjects completing study; TChol = total cholesterol; SD = standard deviations; diff = difference; # = number of**;** NR = not reported; IQR = interquartile range; wks = weeks; mos = months; yr = year.

Study/Year	Duration	*n* =	Ave baseline TChol mg/dL	Baseline TChol SD ±	TChol SD lower value	TChol SD upper value	Ave baseline TChol x *n*	Final ave TChol mg/dL	Final TChol SD ±	TChol SD lower value	TChol SD upper value	Ave Final TChol x *n*	Double check for final ave diffs: Ave baseline minus ave final values mg/dL	Ave diff * *n*	% diff
**Long Duration - 1 and 2 years**															
Vijayakumar 2016	2 years	96	149.8	29.9	119.9	179.7	14382	149.2	28.6	120.6	177.8	14323	-0.6	-58.6	-0.4
Vijayakumar 2016	1 year	96	149.8	29.9	119.9	179.7	14382	144.6	30.9	113.7	175.5	13880	-5.2	-502.1	-3.5
**Medium Duration - 8 to 24 weeks**															
Vijayakumar 2016	3 months	96	149.8	29.9	119.9	179.7	14382	151.2	30.2	121.0	181.3	14514	1.4	132.5	0.9
Assuncao 2009	12 weeks	20	192.5	41.2	151.3	233.7	3850	198.1	39.0	159.1	237.1	3962	5.6	112.0	2.9
Oliveira-de-Lira 2018	8 weeks	18	215.6	17.9	197.7	233.4	3880	198.0	17.6	180.4	215.6	3564	-17.6	-316.1	-8.1
Mendis 1990	8 weeks	25	179.4	14.3	165.1	193.7	4486	178.3	15.1	163.2	193.4	4457	-1.2	-29.0	-0.6
Korrapati 2019	8 weeks	9	193.0	14.4	178.6	207.4	1737	193.0	16.1	176.9	209.1	1737	0.0	0.0	0.0
Cardosa 2015	3 months	92	177.4	51.8	125.6	229.2	16321	183.3	35.4	147.9	218.7	16864	5.9	542.8	3.3
Chinwong 2017	8 weeks	32	190.4	NR			6093	187.7	NR			6006	-2.7	-86.4	-1.4
Fernando 2023	24 weeks	43	206.3	51.0	155.3	257.3	8871	199.6	44.7	154.9	244.3	8583	-6.7	-288.1	-3.2
Jeyakumar 2023	8 weeks	22	172.0	5.6	166.4	177.6	3784	186.0	5.9	180.1	191.9	4092	14.0	308.0	8.1
Swarnamali 2024	8 weeks	37	201.7	28.0	173.7	229.7	7463	209.8	36.8	173.0	246.6	7763	8.1	299.7	4.0
Teng 2024	12 weeks	48	206.9	NR			9930	187.6	NR			9002	-19.3	-927.8	-9.3

							**Short Duration**	**3 to 7 weeks**							
Khaw 2018	4 weeks	28	228.2	38.4	189.8	266.5	6388	236.7	21.3	215.4	257.9	6626	8.5	238.3	3.7
Reiser 1985	5 weeks	16	158.0	5.3	152.7	163.3	2528	168.0	3.0	165.0	171.0	2688	10.0	160.0	6.3
Voon 2011	5 weeks	45	182.1	25.5	156.6	207.7	8196	191.4	26.7	164.7	218.1	8614	9.3	418.1	5.1
Lu 1997	3 weeks	15	162.4	17.0	145.4	179.4	2436	153.1	14.7	138.4	167.8	2297	-9.3	-139.5	-5.7
Harris 2017	28 days	12	219.6	32.6	187.0	252.2	2635	237.8	24.1	213.7	261.9	2854	18.2	218.4	8.3
Heber 1992	3 weeks	9	165.0	7.0	158.0	172.0	1485	195.0	7.0	188.0	202.0	1755	30.0	270.0	18.2
McKenney 1995 Study 1	6 weeks	11	222.3	25.3	197.0	247.6	2445	233.3	19.0	214.3	252.3	2566	11.0	121.0	4.9
McKenney 1995 Study 2	6 weeks	17	214.0	20.4	193.6	234.4	3638	208.3	20.0	188.3	228.3	3541	-5.7	-96.9	-2.7
Maki 2018	4 weeks	12	188.0	median	178 IQR	215 IQR	2256	201.3	median	IQR 185.9	IQR 212.63	2416	13.3	160.1	7.1
Cox 1995 (Men)	6 weeks	13	251.4	23.2	228.2	274.6	3268	255.0	34.0	221.0	289.0	3315	3.7	47.5	1.5
Cox 1995 (Women)	6 weeks	15	239.8	30.9	208.8	270.7	3596	243.0	24.0	219.0	267.0	3645	3.3	48.8	1.4
Cox 1998	4 weeks	37	212.7	37.1	175.6	249.8	7869	211.5	35.2	176.3	246.7	7826	-1.2	-42.9	-0.5
Schwab 1995	4 weeks	15	186.8	5.8	181.0	192.6	2802	187.6	6.2	181.4	193.7	2813	0.8	11.7	0.4
Vogel 2020	45 days	15	180.1	39.4	140.7	219.4	2701	171.5	49.4	122.0	220.9	2572	-8.6	-129.0	-4.8
Nikooei 2021	4 weeks	22	206.3	41.1	165.3	247.4	4539	239.0	53.6	185.3	292.6	5257	32.6	717.9	15.8
Setyawati 2023	30 days	68	247.7	9.0	238.7	256.6	16843	226.6	8.1	218.6	234.7	15409	-21.1	-1433.4	-8.5
													**Sum of average differences:**	-243.3	

TChol Results

Calculation of total cholesterol average baseline, average final values, average differences, and percent differences:

	n **=**	***n*** **x ave baseline values**	***n*** **x ave final values**	**TChol ave baseline value**	**TChol ave final value**	**TChol ave diff mg/dL**	**% diff**	**Double check: Sum of ave diffs/*n***
**All Studies Combined**	984	183185	182942	186.2	185.9	-0.25	-0.13	-0.25
**Long Duration Studies**	192	28764	28203	149.8	146.9	-2.9	-1.9	
**Medium Duration Studies**	442	80796	80544	182.8	182.2	-0.57	-0.31	
**Short Duration Studies**	350	73625	74195	210.4	212.0	1.63	0.77	

LDL Cholesterol Data Set, Calculations, and Results for 26 Studies of People Consuming Coconut Oil

File S3. Table S3. LDL cholesterol data set for analysis of 26 studies of people consuming coconut oil, including baseline and final values, standard deviations (as reported by the investigators), ranges, ranges for baseline and final values, average differences and percent differences. Beneath the table are shown the calculations for average baseline and final total cholesterol values, average differences and percent differences for all studies combined, and for long-, medium-, and short-duration studies. n = number of subjects completing study; LDL-C = low density lipoprotein cholesterol; SD = standard deviations; diff = difference; # = number of; NR = not reported; IQR = interquartile range; wks = wks; mos = months; yr = year.

Study/Year	Duration	*n* =	Ave baseline LDL-C mg/dL	Baseline LDL-C SD ±	LDL-C SD lower value	LDL-C SD upper value	Ave baseline LDL-C x *n*	Final ave LDL-C mg/dL	Final LDL-C SD ±	LDL-C SD lower value	LDL-C SD upper value	Ave Final LDL-C x *n*	Double check for final ave diffs		
													Ave baseline minus ave final values mg/dL	Ave diff * *n*	% diff
**Long Duration - 1 and 2 years**															
Vijayakumar 2016	2 yrs	96	90.3	24.4	65.9	114.7	8667.8	91.0	21.8	69.2	112.9	8740	0.8	72.0	0.8
Vijayakumar 2016	1 yr	96	90.3	24.4	65.9	114.7	8667.8	91.0	20.7	70.4	111.7	8738	0.7	70.1	0.8
**Medium Duration - 8 to 24 wks**															
Vijayakumar 2016	3 mos	96	90.3	24.4	65.9	114.7	8667.8	89.3	24.6	64.7	113.9	8574	-1.0	-94.1	-1.1
Assuncao 2009	12 wks	20	112.6	37.8	74.8	150.4	2252.0	116.5	36.8	79.7	153.3	2330	3.9	78.0	3.5
Oliveira-de-Lira 2018	8 wks	18	143.2	18.8	124.4	162.0	2578.0	128.3	17.7	110.6	146.1	2310	-14.9	-268.0	-10.4
Mendis 1990	8 wks	25	114.1	16.6	97.5	130.7	2852.0	109.8	14.3	95.5	124.1	2746	-4.3	-106.5	-3.7
Korrapati 2019	8 wks	9	125.0	12.4	112.6	137.4	1125.0	122.0	13.9	108.1	135.9	1098	-3.0	-27.0	-2.4
Cardosa 2015	3 mos	92	108.3	45.2	63.1	153.5	9963.6	112.3	31.2	81.1	143.5	10332	4.0	368.0	3.7
Chinwong 2017	8 wks	32	116.6	NR			3731.2	110.5	NR			3536	-6.1	-195.2	-5.2
Fernando 2023	24 wks	43	141.2	48.4	92.8	189.6	6071.6	130.8	41.5	89.3	172.3	5624	-10.4	-447.2	-7.4
Jeyakumar 2023	8 wks	22	113.0	4.3	108.7	117.3	2486.0	126.0	2.2	123.8	128.2	2772	13.0	286.0	11.5
Swarnamali 2024	8 wks	37	137.2	24.9	112.3	162.1	5077.1	135.2	24.9	110.3	160.1	5003	-2.0	-74.0	-1.5
Teng 2024	12 wks	48	130.3	NR			6255.4	116.0	NR			5568	-14.3	-686.9	-11.0
**Short Duration - 3 to 7 wks**															
Khaw 2018	4 wks	28	135.3	34.8	100.5	170.1	3789.5	131.9	19.0	112.9	150.8	3692	-3.5	-97.4	-2.6
Reiser 1985	5 wks	16	96.0	5.8	90.2	101.8	1536.0	110.0	4.1	105.9	114.1	1760	14.0	224.0	14.6
Voon 2011	5 wks	45	118.3	22.4	95.9	140.8	5324.9	127.6	29.0	98.6	156.6	5742	9.3	417.6	7.8

Lu 1997	3 wks	15	90.1	15.1	75.0	105.2	1351.5	87.8	15.9	71.9	103.6	1317	-2.3	-34.8	-2.6
Harris 2017	28 days	12	124.0	24.7	99.3	148.7	1488.0	137.5	27.2	110.3	164.7	1650	13.5	162.0	10.9
Heber 1992	3 wks	9	104.0	8.0	96.0	112.0	936.0	129.0	8.0	121.0	137.0	1161	25.0	225.0	24.0
McKenney 1995 Study 1	6 wks	11	149.0	20.3	128.7	169.3	1639.0	155.4	19.5	135.9	174.9	1709	6.4	70.4	4.3
McKenney 1995 Study 2	6 wks	17	138.6	17.4	121.2	156.0	2356.2	125.8	12.8	113.0	138.6	2139	-12.8	-217.6	-9.2
Maki 2018	4 wks	12	123.0	median	IQR 105	IQR 142	1476.0	128.7	median	IQR 119.9	IQR 144.5	1544	5.7	67.9	4.6
Cox 1995 (Men)	6 wks	13	166.3	23.2	143.1	189.5	2161.6	171.0	33.0	138.0	204.0	2223	4.7	61.4	2.8
Cox 1995 (Women)	6 wks	15	154.1	34.8	119.3	188.9	2311.2	156.0	25.0	131.0	181.0	2340	1.9	28.8	1.2
Cox 1998	4 wks	37	137.7	34.4	103.2	172.1	5093.4	146.6	29.0	117.6	175.6	5423	8.9	329.3	6.5
Schwab 1995	4 wks	15	112.1	4.6	107.5	116.8	1682.1	110.2	4.6	105.6	114.9	1653	-1.9	-29.0	-1.7
Vogel 2020	45 days	15	112.5	32.2	80.3	144.7	1687.1	101.0	37.2	63.8	138.2	1515	-11.5	-172.1	-10.2
Nikooei 2021	4 wks	22	107.6	21.2	86.3	128.8	2366.1	128.5	26.3	102.2	154.8	2827	21.0	460.9	19.5
Setyawati 2023	30 days	68	179.6	7.8	171.9	187.4	12214.2	141.5	7.2	134.3	148.6	9619	-38.2	-2594.9	-21.2
													**Sum of average differences:**	-2123.2	

LDL-C Results

Calculation of LDL cholesterol average baseline, average final values, average differences, and percent differences:

	***n* =**	***n* x ave baseline values**	***n* x ave final values**	**LDL-C ave baseline value**	**LDL-C ave final value**	**LDL-C ave diff mg/dL**	**% diff**	**Double check: Sum of ave diffs/*n***
**All Studies Combined**	984	115808	113685	117.7	115.5	-2.2	-1.8	-2.2
**Long Duration Studies**	192	17336	17478	90.29	91.03	0.74	0.8	
**Medium Duration Studies**	442	51060	49893	115.5	112.9	-2.6	-2.3	
**Short Duration Studies**	350	47413	46314	135.46	132.33	-3.1	-2.3	

HDL Cholesterol Data Set, Calculations, and Results for 26 Studies of People Consuming Coconut Oil

File S3. Table S4. HDL cholesterol data set for analysis of 26 studies of people consuming coconut oil, including baseline and final values, standard deviations (as reported by the investigators), ranges, ranges for baseline and final values, average differences and percent differences. Beneath the table are shown the calculations for average baseline and final total cholesterol values, average differences and percent differences for all studies combined, and for long-, medium-, and short-duration studies. *n* = number of subjects completing study; HDL-C = high density lipoprotein cholesterol; SD = standard deviations; diff = difference; # = number of**;** NR = not reported; IQR = interquartile range; wks = weeks; mos = months; yr = year.

Study/Year	Duration	*n* =	Ave baseline HDL-C mg/dL	Baseline HDL-C SD ±	Baseline HDL-C SD lower value	Baseline HDL-C SD upper value	Ave baseline HDL-C x *n*	Final ave HDL-C mg/dL	Final HDL-C SD ±	Final HDL-C SD lower value	Final HDL-C SD upper value	Ave Final HDL-C x *n*	Double check for final ave diffs		
													Ave baseline minus ave final values mg/dL	Ave diff * *n*	% diff
**Long Duration - 1 and 2 years**															
Vijayakumar 2016	2 yrs	96	40.8	9.2	31.6	50.0	3916.8	43.2	10.8	32.5	54.0	4149	2.4	232.3	5.9
Vijayakumar 2016	1 yr	96	40.8	9.2	31.6	50.0	3916.8	42.4	9.5	32.9	51.9	4071	1.6	154.6	3.9
**Medium Duration - 8 to 24 weeks**															
Vijayakumar 2016	3 mos	96	40.8	9.2	31.6	50.0	3916.8	40.8	10.9	29.9	51.7	3919	0.0	1.9	0.0
Assuncao 2009	12 wks	20	45.5	7.1	38.4	52.6	910.0	48.7	2.4	46.3	51.1	974	3.2	64.0	7.0
Oliveira-de-Lira 2018	8 wks	18	52.9	7.9	45.0	60.9	952.9	55.6	6.4	49.3	62.0	1001	2.7	48.1	5.0
Mendis 1990	8 wks	25	42.5	9.7	32.9	52.2	1063.5	44.1	10.4	33.6	54.5	1102	1.5	38.5	3.6
Korrapati 2019	8 wks	9	46.1	3.3	42.8	49.4	414.9	50.9	3.4	47.5	54.3	458	4.8	43.2	10.4
Cardosa 2015	3 mos	92	37.5	9.2	28.3	46.7	3450.0	40.6	7.4	33.2	48.0	3735	3.1	285.2	8.3
Chinwong 2017	8 wks	32	60.3	NR			1929.6	64.2	NR			2054	3.9	124.8	6.5
Fernando 2023	24 wks	43	44.7	7.4	37.3	52.1	1922.1	46.9	5.9	41.0	52.8	2017	2.2	94.6	4.9
Jeyakumar 2023	8 wks	22	35.0	1.3	33.7	36.3	770.0	34.9	1.8	33.1	36.7	768	-0.1	-2.2	-0.3
Swarnamali 2024	8 wks	37	42.6	10.1	32.5	52.7	1576.2	43.1	11.5	31.6	54.6	1595	0.5	18.5	1.2
Teng 2024	12 wks	48	55.3	NR			2654.4	50.3	NR			2413	-5.0	-241.4	-9.1
**Short Duration - 3 to 7 weeks**															
Khaw 2018	4 wks	28	77.3	19.3	58.0	96.7	2165.5	88.2	11.2	77.0	99.4	2469	10.8	303.2	14.0
Reiser 1985	5 wks	16	45.0	1.1	43.9	46.1	720.0	46.0	1.1	44.9	47.1	736	1.0	16.0	2.2
Voon 2011	5 wks	45	47.6	10.8	36.7	58.4	2140.2	53.0	11.6	41.4	64.6	2384	5.4	243.9	11.4

Lu 1997	3 wks	15	53.0	10.8	42.2	63.8	794.7	49.5	8.1	41.4	57.6	743	-3.5	-52.2	-6.6
Harris 2017	28 days	12	63.9	16.2	47.7	80.1	766.8	70.5	18.8	51.7	89.3	846	6.6	79.2	10.3
Heber 1992	3 wks	9	40.0	3.0	37.0	43.0	360.0	42.0	4.0	38.0	46.0	378	2.0	18.0	5.0
McKenney 1995 Study 1	6 wks	11	49.8	18.3	31.5	68.1	547.8	53.9	15.9	38.0	69.8	593	4.1	45.1	8.2
McKenney 1995 Study 2	6 wks	17	50.9	13.1	37.8	64.0	865.3	56.1	12.4	43.7	68.5	954	5.2	88.4	10.2
Maki 2018	4 wks	12	46.0	median	IQR 38.5	IQR 55.5	552.0	49.0	median	IQR 47.2	IQR 54.19	588	3.0	35.9	6.5
Cox 1995 (Men)	6 wks	13	46.4	7.7	38.7	54.1	603.2	45.0	6.0	39	51.0	585	-1.4	-18.2	-3.0
Cox 1995 (Women)	6 wks	15	69.6	11.6	58.0	81.2	1044.2	68.0	11.0	57	79.0	1020	-1.6	-24.2	-2.3
Cox 1998	4 wks	37	42.5	8.5	34.0	51.1	1574.0	46.8	10.4	36	57.2	1731	4.3	157.3	10.0
Schwab 1995	4 wks	15	59.9	2.3	57.6	62.3	899.1	57.6	2.7	55	60.3	864	-2.3	-34.8	-3.9
Vogel 2020	45 days	15	39.4	11.6	27.8	51.0	591.0	43.1	14.9	28	57.9	646	3.7	55.1	9.3
Nikooei 2021	4 wks	22	44.2	7.8	36.4	52.0	972.0	52.5	7.8	45	60.3	1155	8.3	183.0	18.8
Setyawati 2023	30 days	68	39.9	1.4	38.5	41.4	2714.6	49.3	2.3	47	51.6	3354	9.4	639.2	23.5
													**Sum of average differences:**	2596.9	

HDL-C Results

Calculation of HDL cholesterol average baseline, average final values, average differences, and percent differences:

	***n* =**	***n* x ave baseline values**	***n* x ave final values**	**HDL-C ave baseline value**	**HDL-C ave final value**	**HDL-C ave diff mg/dL**	**% diff**	**Double check: Sum of ave diffs/*n***
**All Studies Combined**	984	44704	47301	45.4	48.1	2.6	5.8	2.64
**Long Duration Studies**	192	7834	8220	40.8	42.8	2.0	4.9	
**Medium Duration Studies**	442	19560	20036	44.3	45.3	1.1	2.4	
**Short Duration Studies**	350	17310	19045.2	49.5	54.4	5.0	10.0	

Triglycerides Data Set, Calculations, and Results for 26 Studies of People Consuming Coconut Oil

File S3. Table S5. Triglycerides data set for analysis of 26 studies of people consuming coconut oil, including baseline and final values, standard deviations (as reported by the investigators), ranges for baseline and final values, average differences and percent differences. Beneath the table are shown the calculations for average baseline and final total cholesterol values, average differences and percent differences for all studies combined, and for long-, medium-, and short-duration studies. n = number of subjects completing study; TG = triglycerides; SD = standard deviations; diff = difference; # = number of; NR = not reported; IQR = interquartile range; wks = wks; mos = mos; yr = year.

**Study/Year**	**Duration**	***n* =**	**Ave baseline TG mg/dL**	**Baseline TG SD ±**	**Baseline TG SD lower value**	**Baseline TG SD upper value**	**Ave baseline TG x *n***	**Ave final TG mg/dL**	**Final TG SD ±**	**Final TG SD lower value**	**Final TG SD upper value**	**Ave Final TG x *n***	**Double check for final ave diffs**		
													**Ave baseline minus ave final values mg/dL**	**Ave diff * *n***	**% diff**
**Long Duration - 1 and 2 years**															
Vijayakumar 2016	2 yrs	96	115.0	54.2	60.7	169.2	11036	109.3	47.1	62.3	156.4	10495	-5.6	-541.44	-4.9
Vijayakumar 2016	1 yr	96	115.0	54.2	60.7	169.2	11036	112.0	50.2	61.8	162.2	10752	-3.0	-284.16	-2.6
**Medium Duration - 8 to 24 wks**															
Vijayakumar 2016	3 mos	96	115.0	54.2	60.7	169.2	11036	111.2	24.8	86.5	136.0	10678	-3.7	-358	-3.2
Assuncao 2009	12 wks	20	172.8	88.1	84.7	260.9	3456	179.7	93.7	86.0	273.4	3594	6.9	138	4.0
Oliveira-de-Lira 2018	8 wks	18	130.9	38.2	92.7	169.1	2356	98.3	29.1	69.2	127.4	1770	-32.6	-586	-24.9
Mendis 1990	8 wks	25	125.8	40.0	85.8	165.8	3144.5	128.4	36.3	92.1	164.8	3211	2.7	66	2.1
Korrapati 2019	8 wks	9	106.0	21.7	84.3	127.7	954	99.0	24.0	75.0	123.0	891	-7.0	-63	-6.6
Cardosa 2015	3 mos	92	153.8	71.2	82.6	225.0	14150	151.8	70.5	81.3	222.3	13966	-2.0	-184	-1.3
Chinwong 2017	8 wks	32	67.8	NR			2169.6	64.7	NR			2070	-3.1	-99	-4.6
Fernando 2023	24 wks	43	104.9	43.0	61.9	147.9	4510.7	109.2	42.8	66.4	152.0	4696	4.3	185	4.1
Jeyakumar 2023	8 wks	22	117.0	14.8	102.2	131.8	2574	124.0	12.3	111.7	136.3	2728	7.0	154	6.0
Swarnamali 2024	8 wks	37	132.5	58.8	73.7	191.3	4902.5	133.7	56.8	76.9	190.5	4947	1.2	44	0.9
Teng 2024	12 wks	48	105.4	NR			5059.2	104.5	NR			5017	-0.9	-42	-0.8
**Short Duration - 3 to 7 wks**															
Khaw 2018	4 wks	28	78.8	median	IQR 65.5	IQR 97.4	2207.2	85.0	51.4	33.7	136.4	2381	6.2	173.6	7.9
Reiser 1985	5 wks	16	80.0	7.5	72.5	87.5	1280.0	78.0	3.6	74.4	81.6	1248	-2.0	-32.0	-2.5
Voon 2011	5 wks	45	85.0	39.0	46.1	124.0	3826.4	79.7	34.5	45.2	114.3	3587	-5.3	-239.0	-6.2
Lu 1997	3 wks	15	93.0	39.0	54.0	132.0	1395.0	79.7	26.6	53.1	106.3	1196	-13.3	-199.4	-14.3
Harris 2017	28 days	12	117.2	97.7	19.5	214.9	1406.4	107.5	80.6	26.9	188.1	1290	-9.7	-116.4	-8.3
Heber 1992	3 wks	9	93.0	13.0	80.0	106.0	837.0	110.0	23.0	87.0	133.0	990	17.0	153.0	18.3

McKenney 1995 Study 1	6 wks	11	117.1	49.2	67.9	166.3	1288.1	120.0	47.7	72.3	167.7	1320	2.9	31.9	2.5
McKenney 1995 Study 2	6 wks	17	122.3	58.2	64.1	180.5	2079.1	131.8	64.8	67.0	196.6	2241	9.5	161.5	7.8
Maki 2018	4 wks	12	92.5	median	IQR 76.5	IQR 136	1110.0	98.0	median	IQR 89.7	IQR 104.7	1176	5.5	66.0	5.9
Cox 1995 (Men)	6 wks	13	203.7	88.6	115.2	292.3	2648.4	231.0	97.0	134.0	328.0	3003	27.3	354.6	13.4
Cox 1995 (Women)	6 wks	15	124.0	35.4	88.6	159.4	1860.0	115.0	27.0	88.0	142.0	1725	-9.0	-135.0	-7.3
Cox 1998	4 wks	37	156.8	100.1	56.7	256.9	5800.9	142.6	82.4	60.2	225.0	5276	-14.2	-524.7	-9.0
Schwab 1995	4 wks	15	80.6	9.7	70.9	90.3	1209.0	77.1	8.0	69.1	85.0	1156	-3.5	-53.1	-4.4
Vogel 2020	45 days	15	141.1	71.4	69.7	212.4	2116.1	138.9	78.3	60.6	217.2	2083	-2.2	-33.0	-1.6
Nikooei 2021	4 wks	22	216.5	153.0	63.5	369.5	4763.0	172.1	114.6	57.6	286.7	3787	-44.4	-975.9	-20.5
Setyawati 2023	30 days	68	192.8	3.3	189.5	196.0	13108.4	183.5	5.0	178.6	188.5	12481	-9.2	-627.6	-4.8
													**Sum of average differences:**	-3566.0	

TG Results

Calculation of triglycerides average baseline, average final values, average differences, and percent differences:

	n **=**	***n*** **x ave baseline values**	***n*** **x ave final values**	**TG ave baseline value**	**TG ave final value**	**TG ave diff mg/dL**	**% diff**	**Double check: Sum of ave diffs/*n***
**All Studies Combined**	984	123319	119753	125.3	121.7	-3.6	-2.9	-3.6
**Long Duration Studies**	192	22072.3	21247	115.0	110.7	-4.3	-3.7	
**Medium Duration Studies**	442	54312.3	53567	122.9	121.2	-1.7	-1.4	
**Short Duration Studies**	350	46934.8	44939	134.1	128.4	-5.7	-4.3	

References:

Vijayakumar, M., Vasudevan, D.M., Sundaram, K.R., Krishnan, S., Vaidyanathan, K., Nandakumar, S., Chandrasekhar, R., Mathew, N. A randomized study of coconut oil versus sunflower oil on cardiovascular risk factors in patients with stable coronary heart disease. *Indian Heart J*, 2016, 68, 498–506.

Mendis, S., Kumarasunderam, R. (1990). The effect of daily consumption of coconut fat and soya-bean fat on plasma lipids and lipoproteins of young normolipidaemic men. *Br J Nutr*, 63, 541-552

Oliveira-de-Lira, L., Santos, E.M.C., de Souza, R.F., Matos, R.J.B., Silva, M.C.D., Oliveira, L.D.S., Nascimento, T.G.D., Schemly, P., Souza, S.L. Supplementation-dependent effects of vegetable oils with varying fatty acid compositions on anthropometric and biochemical parameters in obese women. *Nutrients*, 2018, 20, E932.

Korrapati, D., Jeyakumar, S.M., Putcha, U.K., Mendu, V.R., Ponday, L.R., Acharya, V., Koppala, S.R., Vajreswari, A. Coconut oil consumption improves fat-free mass, plasma HDL-cholesterol and insulin sensitivity in healthy men with normal BMI compared to peanut oil. *Clin Nutr*, 2019, 38, 2889–2899.

Chinwong, S., Chinwong, D., & Mangklabruks, A. Daily consumption of virgin coconut oil increases high-density lipoprotein cholesterol levels in healthy volunteers: A randomized crossover trial. *Evid Based Complement Altern Med*, 2017, 7251562. Epub 2017 Dec 14.

Jeyakumar, S. M., Damayanti, K., Rajkumar Ponday, L., Acharya, V., Koppala, S. R., Putcha, U. K., Nagalla, B., & Vajreswari, A. Assessment of virgin coconut oil in a balanced diet on indicators of cardiovascular health in non-obese volunteers: A human metabolic study. *Diabetes Metab Syndr*, 2023, 17(9), 102844.

Swarnamali, H., Ranasinghe, P., Jayawardena, R. Changes in serum lipids following consumption of coconut oil and palm olein oil: A sequential feeding crossover clinical trial. *Diabetes Metab Syndr*, 2024, 18(6), 103070.

Assunção, M.L., Ferreira, H.S., dos Santos, A.F., Cabral, C.R. Jr, & Florêncio, T.M. Effects of dietary coconut oil on the biochemical and anthropometric profiles of women presenting abdominal obesity. *Lipids*, 2009, 44, 593–601.

Teng, K.T., Loganathan, R., Chew, B.H., Khang, T.F. Diverse impacts of red palm olein, extra virgin coconut oil and extra virgin olive oil on cardiometabolic risk markers in individuals with central obesity: a randomised trial. *Eur J Nutr*, 2024, 63(4), 1225-1239.

Cardoso, D.A., Moreira, A.S., de Oliveira, G.M., Raggio Luiz, R., & Rosa, G. A coconut extra virgin oil-rich diet increases HDL cholesterol and decreases waist circumference and body mass in coronary artery disease patients. *Nutricion Hospitalaria*, 2015, 32(5), 2144-52.

Fernando, M. G., Silva, R., Fernando, W. M. A. D. B., de Silva, H. A., Wickremasinghe, A. R., Dissanayake, A. S., Sohrabi, H. R., Martins, R. N., & Williams, S. S. Effect of Virgin Coconut Oil Supplementation on Cognition of Individuals with Mild-to-Moderate Alzheimer's Disease in Sri Lanka (VCO-AD Study): A Randomized Placebo-Controlled Trial. *J Alzheimers Dis*, 2023, 96(3), 1195–1206.

Heber, D., Ashley, J. M., Solares, M. E., Wang, H. J., & Alfin-Slater, R. B. The effects of a palm-oil enriched diet on plasma lipids and lipoproteins in healthy young men. *Nutr Res*, 1992, 12, S53-59.

Lu, Z., Hendrich, S., Shen, N., White, P.J., Cook, L.R. Low linolenate and commercial soybean oils diminish serum HDL cholesterol in young free-living adult females. *J Am Coll Nutr*, 1997,16, 562–569.

Schwab, U.S., Niskanen, L.K., Maliranta, H.M., Savolainen, M.J., Kesäniemi, Y.A., Uusitupa, M.I. Lauric and palmitic acid-enriched diets have minimal impact on serum lipid and lipoprotein concentrations and glucose metabolism in healthy young women. *J Nutr*, 1995,125(3), 466-73.

Cox, C., Sutherland, W., Mann, J., de Jong, S., Chisholm, A., & Skeaff, M. Effects of dietary coconut oil, butter, and safflower oil on plasma lipids, lipoproteins, and lathosterol levels. *Eur J Clin Nutr*, 1998, 52(9), 650-654.

Harris, M., Hutchins, A., & Fryda, L. The impact of virgin coconut oil and high oleic safflower oil on body composition, lipids, and inflammatory markers in postmenopausal women. *J Med Food* 2017, 20, 345–351.

Khaw, K.T., Sharp, S.J., Finikarides, L., Afzal, I., Lentjes, M., Luben, R., Forouhi, N.G. Randomised trial of coconut oil, olive oil or butter on blood lipids and other cardiovascular risk factors in healthy men and women. *BMJ Open*, 2018, 8, e020167.

Maki, K.C., Hasse, W., Dicklin, M.R., Bell, M., Buggia, M.A., Cassens, M.E., Eren, F. Corn oil lowers plasma cholesterol compared with coconut oil in adults with above-desirable levels of cholesterol in a randomized crossover trial. *J Nutr*, 2018, 148, 1556–1563.

Nikooei, P., Hosseinzadeh-Attar, M.J., Asghari, S., Norouzy, A., Yaseri,M., Vasheghani-Farahani, A. Effects of virgin coconut oil consumption on metabolic syndrome components and asymmetric dimethylarginine: A randomized controlled clinical trial. *Nutr Metab Cardiovasc Dis*, 2021, 31(3), 939-949.

Setyawati, A., Sangkala, M.S., Malasari, S., Jafar, N., Sjattar, E.L., Syahrul, S., Rasyid, H. Virgin coconut oil: a dietary intervention for dyslipidaemia in patients with diabetes mellitus. *Nutrients*, 2023, 15(3), 564.

Reiser, R., Probstfield, J.L., Silvers, A., Scott, L.W., Shorney, M.L., Wood, R.D., O'Brien, B.C., Gotto, A.M., Jr., Insull, W., Jr. Plasma lipid and lipoprotein response of humans to beef fat, coconut oil and safflower oil. *Am J Clin Nutr*, 1985, 42, 190–197.

Voon, P.T., Ng, T.K., Lee, V.K., Nesaretnam, K. Diets high in palmitic acid (16:0), lauric and myristic acids (12:0 + 14:0), or oleic acid (18:1) do not alter postprandial or fasting plasma homocysteine and inflammatory markers in healthy Malaysian adults. *Am J Clin Nutr*, 2011, 94, 1451-1457.

Cox, C., Mann, J., Sutherland, W., Chisholm, A., & Skeaff, M. Effects of coconut oil, butter, and safflower oil on lipids and lipoproteins in persons with moderately elevated cholesterol levels. *J Lipid Res* 1995, 36(8), 1787-1795.

McKenney, J.M., Proctor, J.D., Wright, J.T., Jr., Kolinski, R.J., Elswick, R.K., Jr., Coaker, J.S. The effect of supplemental dietary fat on plasma cholesterol levels in lovastatin-treated hypercholesterolemic patients. *Pharmacotherapy*, 1995, 15, 565–572.

Vogel, C.É., Crovesy, L., Rosado, E.L., Soares-Mota, M. Effect of coconut oil on weight loss and metabolic parameters in men with obesity: a randomized controlled clinical trial. *Food Funct*, 2020, 11(7), 6588-6594.

Disclaimer/Publisher's Note: The statements, opinions and data contained in all publications are solely those of the individual author(s) and contributor(s) and not of MDPI and/or the editor(s). MDPI and/or the editor(s) disclaim responsibility for any injury to people or property resulting from any ideas, methods, instructions or products referred to in the content.
