# Supplementary material for: Analysis of 26 Studies of the Impact of Coconut Oil on Lipid Parameters: Beyond Total and LDL Cholesterol
Source: Nutrients. 2025 Jan 30;17(3):514. doi: 10.3390/nu17030514 (PMC11819987; doi:10.3390/nu17030514)
Supplement: Supplementary file 1 [file nutrients-17-00514-s001.zip › File S1_Narrative descriptions of twenty-six studies.pdf]

Analysis of 26 Studies of the Impact of Coconut Oil on Lipid Parameters: Beyond Total and LDL Cholesterol

By Mary T. Newport, M.D. and Fabian M. Dayrit, Ph.D.

File S1: Narrative descriptions of twenty-six studies in order of year published

Reiser et al. (1985) [1], which was conducted in Texas, USA, was a crossover study of 19 healthy male medical students aged 22 to 29 years with normal lipid profiles, each of whom completed at least two of three test diet periods. The aim of the study was to compare the effects on lipid profiles of beef fat (with about 53% unsaturated fat) versus coconut oil to represent saturated fat and safflower oil to represent polyunsaturated fat. There were seven diet periods each lasting five weeks; during the odd-numbered periods, the participants consumed their habitual diet, and during the even-numbered periods, they were randomly assigned to a sequence of test diets containing coconut oil (CNO), safflower oil, or beef fat. The oils were not otherwise described and were provided by a vegetable oil and a food company. The participants were instructed to eat a low-fat high-carbohydrate breakfast and were provided with lunch and dinner meals that contained 50% carbohydrate, 15% protein, and 35% fat of which 65% was the test oil or fat. The test oil/fat represented about 21% of total calories and egg yolk was added as needed to provide 450 grams of cholesterol daily in the two provided meals. The fat was incorporated into "common" foods such as meat dishes, bread spreads, ice cream and cookies. Snacks, desserts, and moderate alcohol consumption were allowed, and total caloric intake varied from 1700 to 5000 kcal daily (average 3400 kcal daily). The test fat would add up to 80 to 117 gm/day based on total calories. Fasting bloodwork was drawn at baseline, the end of the study, and between each diet test period. **See File S2 Table S1 for lipid profile results.** At the end of the coconut oil test period, compared to baseline, total cholesterol (TChol) and low-density lipoprotein cholesterol (LDL-C) were higher, and high-density lipoprotein cholesterol (HDL-C) and triglycerides (TG) were lower, whereas for the beef fat and safflower oil test periods, TChol, LDL-C, and HDL-C were lower, and TG was higher for beef fat and lower for safflower oil. The authors noted that the customary consideration of beef fat as "saturated fat" was unwarranted since the saturated fat content is much lower than CNO, and the cholesterol values decreased in this study.

Mendis et al. (1990) [2], which was conducted in Sri Lanka, was a crossover study of 25 young healthy male prisoners aged 20 to 26 with normal weight and lipid profiles. The typical Sri Lankan diet at that time was noted to be high in coconut fat, though the fat content of the prison diet was not provided. The aim of the study was to look at the effect of replacing CNO with soybean oil on fasting lipids. The type of fat and the sources were not further described beyond "coconut fat" or "soya-bean fat". The study began with an eight-week test period of soybean oil, followed by a three-week washout period of the usual prison diet, and "reintroduction" of an equivalent amount of CNO for eight weeks. Breakfast consisted of a low-fat high-carbohydrate meal, and the test oils were provided in 14 different lunch and dinner meals that were rotated throughout the study periods. Cheese, butter, and alcohol were not permitted. Overall, the soybean and CNO diets each provided about 2400 kcal daily with about 30% of total calories as fat, of which 70% was the test oil (about 55 gram/day) and the other 30% from meat, fish, poultry, grains, and vegetables. The fasting lipid profile was measured at the beginning and end of each test period. **See File S2 Table S1 for lipid profile results.** Both soybean oil and CNO resulted in lower TChol, LDL-C, and TG levels (soybean more so than CNO) compared to baseline values; however, CNO increased HDL-C, whereas soybean oil decreased HDL-C. The authors noted that the "short-chain fats" (in reference to CNO) appeared to have a neutral effect on TChol, and that the drop in HDL-C from substituting a polyunsaturated fatty acid (PUFA) oil for coconut fat could be undesirable in the Sri Lankan population.

Heber et al. (1992) [3] was conducted at University of California Los Angeles to compare differences in fasting lipid profile values for men consuming palm oil, CNO, and hydrogenated soybean oil. The authors noted that hydrogenated soybean oil had been substituted for palm oil by many baked goods manufacturers in the US at that time to promote "good health" based on recommendations of the American Heart Association (AHA) to increase the ratio of PUFA to saturated fatty acid (SFA) fats in the diet to reduce plasma cholesterol levels. The authors noted further that "other chemical properties of fats such as chain length of fatty acids of triglycerides, the number of double bonds, and the position of double bonds also determine the effects of the dietary intake of a particular fat on plasma lipids". The specific oils were not otherwise described, and the sources were not provided. Just nine healthy men aged 22 to 43, not otherwise described, completed the entire study. All had TChol less than 180 mg/dl and LDL-C less than 130 mg/dl at entry. The subjects were assigned to complete three test periods of three weeks each consuming either palm, coconut, or

hydrogenated soybean oil in random order with a two-week washout habitual diet period between the test oil periods. The oils were provided in muffins and cookies each with 13.7 gm of the test oil, 231 kcal, 3.5 gm protein, and 23.7 gm carbohydrate, and the men were instructed to otherwise consume a low-fat diet. The diet overall contained 17.5% of energy as the test fat, approximately 41 gm daily, the amount in three muffins and/or cookies, and the men averaged 2140 kcal daily on average during the soybean oil diet period and 2380 kcal daily during the coconut and palm oil diet periods. Fasting lipid profiles were drawn at the beginning and end of each three-week test period. **See File S2 Table S1 for lipid profile results.** All lipid values increased at the end of the CNO test period; there were no significant differences in any lipid value for soybean oil. For palm oil there was no significant difference for TChol, LDL-C, or TGs, although there was a significant difference for lowering the LDL-C to HDL-C ratio. There was no change in body weight for any of the three diets.

The authors recognized that they tested a small number of subjects (n=9), and that "there is a wide variation in the individual response of plasma lipids to changes from dietary saturated to unsaturated fatty acids." They also noted that previous studies of hypercholesterolemic men used extreme percentages of oleic and linoleic acid in liquid formulas compared to amounts present in ordinary foods to conclude that PUFAs reduce cholesterol levels. In this study, hydrogenated soybean oil did not lower cholesterol, and palm oil did not raise cholesterol levels despite providing 17.5% of total calories in the test diet, much higher than the 2% of total dietary fat as palm oil consumed on average in the typical US diet at that time.

Cox et al. (1995) [4] was a crossover study that took place at the University of Otago in New Zealand to explore the physiological effects of coconut oil, butter, and safflower oil on lipids and lipoproteins in people who were moderately hypercholesterolemic. There were 28 participants (13 men, 15 women) aged 29-67 with TChol levels ranging from 212.7 to 305.5 mg/dL and TG levels < 265.7 mg/dL. There was an initial 6-week run-in period during which participants completed a five-day food record while consuming their usual diet followed by three test oil periods lasting 6 weeks each for which subjects were randomized to one of three dietary sequences in which they would consume safflower oil, to represent PUFA, and CNO and butter, to represent saturated fat. Overall, the diets contained 17% protein, 47% carbohydrate, and 36% fat. The dietary fats were provided and were "rigidly" prescribed, so that the CNO and butter diets contained about 39 gm of the test fat based on 20% of total calories as saturated fat, and safflower oil diet provided 24 gm of the oil based on 10% of total calories as PUFA and 10% as SFA with the remained of fat for all diets coming from foods that included lean meat, fish, chicken, and dairy products. During the butter diet, some butter could be exchanged with cream, cheddar cheese, and cakes or muesli bars made with butter. The source and processing method for the CNO used was not reported. Body measurements and blood samples for lipids and lipoproteins were taken before randomization and then at weeks 4 and 6 of each test period. LDL-C was determined using the Friedewald calculation. ApoA-I and ApoB, and cholesteryl ester transfer protein activity (CETA) levels were also done before randomization and at week 4 of each test period. **See File S2 Table S1 for lipid profile results.** Results were reported separately for men and women, thus providing two groups of lipid profile data sets. TChol and LDL-C increased slightly, and HDL-C decreased slightly from baseline values for both men and women when taking CNO, and TG increased for the men and decreased for the women. During the safflower oil test period, TChol and LDL-C decreased slightly for men and women, HDL-C and TG did not change for the men and HDL-C increased slightly and TG decreased slightly for the women. TChol and LDL-C increased more during the butter test period than in the CNO test period for men and women; HDL-C did not change for the men and increased minimally from baseline for the women while taking butter, and TG increased slightly for men and women. The results for TChol and LDL-C were highest for butter and lowest for safflower oil with CNO midway between and the differences were considered significant. Levels of HDL-C for women were found to be significantly higher for butter and CNO compared to safflower oil. ApoA-I levels tended to be higher for CNO and butter than for safflower oil but only reached significance in men. ApoB was lowest for the total group when consuming safflower oil, and the differences in CETA were insignificant. The authors concluded that their data "provide convincing evidence that coconut oil rich in lauric acid has a lesser effect than butter, which is high in palmitic acid, on total and LDL cholesterol in hypercholesterolemic men and women."

McKenney et al. (1995) [5] was conducted at the Medical College of Virginia, USA in two parts using two different groups of subjects with elevated cholesterol levels. **Part one** was a validation study comparing coconut and canola oil to determine whether the oils would increase or decrease cholesterol values, respectively, according to conventional beliefs about saturated and unsaturated fats. The subjects were 11 free-living adults (6 males and 5 females) aged 47 to 79 with TChol levels between 200 and 280 mg/dL (average 222 mg/dL) and average LDL-C of 149 mg/dL completed the study. The duration of the study was 18 weeks divided into three periods each lasting six weeks without a washout period between test periods. For the first six-week period, the subjects were instructed on implementation of the Step 1 diet of

the National Cholesterol Education Program (NCEP), a low-fat, low-cholesterol diet and were expected to maintain the diet throughout all three segments of the study. During the second 6-week test period, the subjects were randomly assigned to receive either canola oil or CNO, then crossed over to the other oil for the third six-week period. The oils were delivered in oatmeal raisin cookies, three cookies per day, made with white flour and white and brown sugar, and calculated to contain about 228 kcal each with 14 gm of calories as the test fat totaling 42 gm daily of the test fat. Average total daily intake was about 2200 kcal including 37 to 38% fat. The CNO brought saturated fat intake up to about 22% of total calories. The three cookies added 684 kcal to the diet and the average person gained 4.7 pounds during the twelve weeks of the canola and CNO segments of the study. The increase in calories, weight gain, and/or high carbohydrate content of the cookies might have affected the results. Fasting lipid profiles were drawn at the end of weeks 4 and 6 of the baseline diet and oil test periods and were averaged to establish the baseline values for the subsequent test period. **See File S2 Table S1 for lipid profile results.** On average, all lipids increased following the CNO test period, whereas TChol and LDL-C decreased, and HDL-C and TG increased following the canola oil test period. The authors noted that during the canola oil test period, TChol decreased in 7 and increased in 4 of the 11 people with a range of -14.1% to +10.6%. For canola oil, 9 of 11 people had a reduction in LDL-C. Likewise, TChol and LDL-C increased in 8 and decreased in 3 of 11 people, by 5% on average, at the end of the CNO test period, but the changes in individual values varied from -10.4% to +25.3%. The authors commented that the increase in TChol with CNO was one-third of the expected value based on "published formulas". This is one of the few studies that provided any information on individual results.

Part Two of the **McKenney et al. (1995)** [5] study included a different free-living group of 17 adults (12 males and 5 females) aged 39 to 63 who had "severely elevated" TChol (baseline average 291 mg/dL) and LDL-C levels >160 mg/dL (baseline average 212 mg/dL) at the end of a six-week period during which they consumed the NCEP Step 1 diet. The study continued with three additional six-week periods. During the second six-week study period, the subjects were placed on 20 or 40 mg daily of lovastatin depending on their individual LDL-C levels at that point in time. During the third six-week period, the subjects consumed three of the same oatmeal raisin cookies described for Part One made with either canola or CNO totaling 42 gm daily, then switched to the other oil for the fourth six-week period. Again, the cookies added 684 kcal to the total baseline daily intake of 2026-2029 kcal with 35 to 36% of calories as fat, and the group on average gained nearly five pounds. Fasting lipid profiles were drawn at the end of weeks 4 and 6 of the baseline diet and oil test periods which were averaged to establish the baseline values for the subsequent test period**. See File S2 Table S1 for lipid profile results.** TChol and LDL-C levels decreased on average in both the canola and CNO segments of the study. After consuming the coconut oil cookies for 6 weeks, TChol decreased in 12 of 17 people by an average of -5.7 mg/dl but with a very broad range between -39.5% to +37% from the baseline. Likewise, 13 of 17 people had a decrease in LDL-C with an average of -12.8 mg/dl, whereas 14 of 17 people saw a decrease in TChol and 15 of 17 a decrease in LDL-C following the canola oil test period. The authors speculated that the lovastatin may have "augmented" the effect of the PUFA canola oil, and "countered" the effect of the SFA coconut oil. As an alternative, they suggested that the added oils may have improved the bioavailability of lovastatin, a lipophilic compound. The authors noted that this group gained 5 pounds on average during the study. The additional calorie and high glycemic carbohydrate intake provided by the cookies, along with the weight gain, could have affected the results in both parts of the study.

Schwab et al. (1995) [6] was a randomized crossover study of effects of lauric acid and palm oil on lipid profiles and other lipoproteins conducted by investigators from the Universities of Kuopio and Oulu in Finland. The subjects were 15 healthy women aged 19 to 34 with normal body weight and BMI 19-25 kg/m^2, who were nonsmokers and not on lipid-lowering medication. The study began with a 2-week baseline period on their usual diet during which baseline fasting laboratory studies, including lipid profile, ApoA-I, ApoB, Lp(a), and cholesteryl ester transfer protein activity (CETA), glucose tolerance tests, fasting blood glucose and insulin, and fatty acid analysis, anthropometric, and blood pressure measurements were obtained. Two test oil period each of 4 weeks duration with a 2-week baseline period between the two test oil periods followed. The women were randomized to begin with either coconut oil (lauric acid diet) or palm oil (palmitic acid diet). The evaluation was repeated at the end of each test oil period as well as at the end of the second baseline period between the test oil periods. LDL-C levels were calculated. The overall diet was designed to maintain body weight and contained 15% protein, 49% carbohydrate, and 36% fat, of which 15% was SFA, 15% MUFA, and 5% PUFA. Of total energy intake, 4% was from either palmitic acid from added palm oil or lauric acid from added coconut oil. The participants received 22-33 gm daily of palm oil or 16-26 gm daily of CNO based on their daily energy intake. The palm oil was refined, bleached and deodorized, and the CNO was "refined". To balance out the fatty acids, a moderate amount of soybean oil was added to the palmitic acid diet, and a mixture of low-erucic -acid rapeseed oil, olive oil, and sunflower oil were added to the lauric acid diet. Medium-fat dairy was also consumed during the baseline and palmitic acid diets, and fat-free and low-fat products during the lauric acid diet. Egg yolk was added to supply an equal

amount of cholesterol to each diet. The fat mixtures were provided, and the diets otherwise contained common Finnish foods. The subjects were asked to weigh and measure their food. **See File S2 Table S1 for lipid profile results.** After consuming CNO, TChol increased slightly by 0.4%; however, LDL-C, HDL-C, and TGs all decreased slightly. With the palmitic acid diet, TChol, LDL-C, HDL-C, and TG all decreased. There were no significant differences in ApoA-I, ApoB, Lp(a), or CETA between the two test oil periods. The glucose tolerance tests revealed no differences between the diet in insulin sensitivity index or glucose effectiveness. As expected, lauric acid and palmitic acid levels were higher during their respective diet periods. The authors concluded that: "In healthy young women, a substitution of 4% energy as palmitic or lauric acid for monounsaturated fatty acids had only minor effects on serum lipids and lipoproteins and caused no changes in glucose metabolism."

Lu et al. (1997) [7] was conducted at Iowa State University to determine the effects on fasting lipid profile and fatty acid levels of a "mutant soybean line" (A16) developed at the University that was lower in C18:3 (2%) than commercial non-hydrogenated soybean oil (7.4%). The fatty acid composition of the CNO used in the study was not typical for non-hydrogenated coconut oil since it was reported to be 100% SFA with no monounsaturated fatty acids (MUFA) or PUFA, whereas CNO normally contains about 5 to 7% MUFA and 2% PUFA, strongly suggesting that this study used hydrogenated CNO. The subjects were 15 healthy female students aged 18 to 24 with normal lipid profiles and BMI. Each oil was tested for 3 weeks over a nine-week period, and the students were assigned to a random order of the test oils. There were no washout periods based on the assumption that 2 weeks was adequate time for the lipid values to stabilize, and there were only 9 consecutive weeks available during the semester to complete the study. The subjects were instructed to add the oils to their daily intake and to reduce (but not eliminate) other high fat foods like cheese, butter, French fries, and potato chips. On average, the subjects consumed 1744 to 1863 kcal daily during the three test periods with about 10% of total kcal as the test oil, averaging 2 tablespoons per day (about 30 ml) with 35 to 36% of total calories as fat. Fasting lipid profiles were drawn at baseline and at the end of the second and third weeks of each test oil period to reduce day-to-day variation, which were then averaged and reported as the final results for each test period. There was no significant difference in weight/BMI at the end of the study. **See File S2 Table S1 for lipid profile results.** TChol, LDL-C, HDL-C, and TGs decreased on average for each of the three test oil groups, and the only significant difference was a smaller decrease in HDL-C for CNO compared to the soybean oil test periods. The authors noted that the results did not conform with the PUFA/SFA ratio equation used to predict effects on blood lipid profile values and speculated that this result could be due to the low number of subjects studied or that the subjects had decreased their intake of other saturated fats during the study, as instructed. The authors concluded that A16 soybean oil could be substituted for partially hydrogenated soybean oil containing *trans*-fats in the diet without having a deleterious effect on the blood lipid profile.

Cox et al. (1998) [8] was conducted at the University of Otago, New Zealand, and aimed to compare the effects of CNO, butter, and safflower oil on plasma lipid, lipoprotein, and lathosterol levels. The authors explain that: the plasma lathosterol level and the ratio of plasma lathosterol to cholesterol are excellent indices of cholesterol synthesis; lathosterol tends to decrease when cholesterol synthesis is reduced during treatment with drugs that reduce HMG CoA reductase, the rate-limiting step in cholesterol biosynthesis; lathosterol increases with increased cholesterol synthesis. The subjects were 41 Pacific Islanders (24 men and 17 women) aged 19 to 72 who previously consumed coconut rich diets but consumed other fats after migrating to Dunedin, New Zealand. Their baseline TChol levels ranged from 162 to 290 mg/dL and their TG levels were < 265.7 mg/dL. The subjects did not use lipid-lowering drugs. The study began with a run-in period of 6 weeks, followed by three consecutive six-week test periods during which diets rich in butter, CNO, and safflower oil were consumed with no washout period between test diet periods. The participants all followed the same sequence since they often ate communally. Body weight and fasting blood samples were taken twice during the baseline period and at 4 and 6 weeks in each test diet period. Lipid profiles were determined at baseline, 4 and 6 weeks, and lathosterol, ApoA-1, ApoA-II, ApoB were measured once at baseline and at 4 weeks during each test diet period. The test diets contained 17% protein, 47% carbohydrate, and 36% fat. Each diet contained 84 gm fat and included 39 gm CNO to provide 17 gm lauric acid in the CNO diet, 39 gm butter plus other palmitic rich foods to provide 17 gm palmitic acid in the butter diet, and 24 gm safflower oil to provide 17 gm linoleic acid in the safflower diet. The processing methods of the oils were not reported. The oils and margarines containing the oils were provided during each test diet period. Egg yolk was added as needed to maintain the same amount of cholesterol in each diet. The subjects were also instructed on other foods they should consume as well as frequent dietary counseling. Body weight did not change during the three diet periods. The plasma lathosterol levels was lowest for safflower oil diet and highest for the butter diet, with CNO in between; however, the plasma lathosterol/ cholesterol ratio was lowest following the CNO diet, indicating lower cholesterol synthesis. **See File S2 Table S1 for lipid profile results.** During the CNO diet period, TChol decreased

slightly, LDL-C increased moderately, HDL-C increased by 10%, and TG decreased by 9%. All lipid profile values and lipoproteins increased while on the butter diet, whereas TChol, LDL-C, HDL-C, ApoA-I, and ApoB were all significantly lower during the safflower oil diet compared to the CNO and butter diets. The authors concluded that their data suggest "that cholesterol synthesis is reduced leading to lower plasma lathosterol levels when butter is replaced by coconut fat in the diet".

Assunção et al. (2009) [9] took place in Brazil to study the effects on anthropometric measurements and lipid profiles of adding soybean or CNO to the diets of women aged 20 to 40 with abdominal obesity, specifically, with a waist circumference of >88 cm (34.6 inches). The women were clinic outpatients of low socioeconomic status. There were two groups with 20 women each assigned to consume 30 ml daily of either soybean oil or CNO. In addition, both groups were counseled to consume more fruits and vegetables, reduce simple sugars and animal fats, reduce, or stop alcohol consumption and smoking, and to walk on a treadmill at the clinic for 50 minutes four times weekly under the supervision of a trainer. The trial period lasted 12 weeks, and measurements and fasting bloodwork were taken one week before the onset and one week after the end of the trial. Filtered CNO pressed from dehydrated coconut at 140°F (60°C) was used and the fatty acid composition was consistent with non-hydrogenated oil. The soybean oil was purchased from a local grocery store and was not otherwise specified. Both groups experienced an average weight loss of 1 kg (2.22 lb), and overall caloric intake decreased during the study, based on 3-day food recall diaries, from an average of 1893 to 1732 kcal daily. Fat intake increased slightly from 19 to 20.8% of total calories, protein and fiber intake increased, and carbohydrate intake decreased by about 50 grams/day. Waist circumference was unchanged for the soybean oil group but decreased significantly for the CNO group by 1.4 cm. **See File S2 Table S1 for lipid profile results.** In the soybean oil group, TChol, LDL-C, and LDL-C/HDL-C ratio all increased significantly, TG did not change, and HDL-C decreased significantly. Whereas, in the CNO group, HDL-C increased significantly, and TChol, LDL-C, and TG trended upward on average but were not significantly different. The authors attributed the decrease in waist circumference to the MCFAs in CNO and concluded that CNO does not cause dyslipidemia.

Voon et al. (2011) [10] was conducted in Malaysia primarily to study the effects of dietary oils on homocysteine levels and inflammatory markers, but the investigators also collected fasting lipid profile data. The subjects were 45 healthy adults (36 women and 9 men) aged 20 to 40 with BMI of 18 to 30 and normal lipid profiles. All meals were provided beginning with a three-week standardization period of Malaysian foods with 30% fat, 15% protein, and 55% carbohydrate. Three groups of 15 people each were randomly assigned to a five-week diet period with meals provided on a five-day rotation differing only in the test oils which were palm olein, CNO, or virgin olive oil. The oils were not otherwise specified as to processing and made up 20% of total calories on the provided 2000 kcal daily diet (about 44 grams) with 30% fat, 2/3 of which was the test oil, 20% protein, and 50% carbohydrate. Fasting bloodwork was drawn after week 2 of the standardization diet and at the end of week 5 of the test diet, including fasting and non-fasting levels for comparison. **See File S2 Table S1 for lipid profile results.** Using fasting values, the CNO and palm olein groups experienced average increases in TChol, LDL-C, and HDL-C, which were less for palm olein than for CNO, though not significantly different between the two oil groups, and lower TG levels which were greater for palm olein. The olive oil group experienced a tiny average decrease in TChol, lower TG, no change in LDL-C, and an increase in HDL-C. Only the average baseline values for all 45 subjects were reported, rather than separate averages for each 15-person group which might have affected the comparisons. Homocysteine levels were significantly higher than baseline values for all three oil groups, though not significantly different between test oil groups, and there were no statistically significant differences in the inflammatory biomarkers between the test oil groups. The authors noted that the changes in lipid profile were very similar for palm olein and virgin olive oil and that postprandial values of Lp(a) were lower for CNO than for the other oils.

Cardoso et al. (2015) [11] was a longitudinal study conducted at the clinical nutrition department of a specialized hospital in Brazil. The subjects were 114 men and women aged 45 to 85 years with diagnosed coronary artery disease who were all hypertensive, and 94.5% were taking medication for dyslipidemia. During the three-month run-in phase of the study, all participants were instructed to consume a diet designed for people with dyslipidemia that was based on the Dietary Reference Intakes (2005) [12] and the National Cholesterol Education Program - Adult Treatment Panel III (NCEP ATPIII) [13]. For the next three months, 92 participants were assigned to add 13 mL daily of virgin coconut oil (VCO) to the new diet which could be added to food but not subjected to heat. The other 22 people were assigned to serve as controls and remain on the new diet. The subjects were evaluated monthly including obtaining a 12-hour fasting blood sample, a 24-hour dietary recall, anthropometric assessment, and blood pressure (BP) measurement. Even though monthly values were obtained, only baseline and final lipid profile values were reported. LDL-C was determined using the Friedewald calculation. **See File S2 Table S1 for lipid profile results.** At the end of the 3 months, both groups

experienced small non-significant increases in TChol and LDL-C. The VCO group experienced a significant increase in HDL-C, whereas HDL-C decreased slightly in the control group. The VCO group had a small decrease in TG compared to an increase in TG in the control group; however, neither TG result was significant. The only significant differences between the two groups were in the positive increase in HDL-C and a greater decrease in waist circumference in the VCO group. The VCO group also had significant increases in Apoprotein A (ApoA), which is considered desirable but also in Apoprotein B (ApoB), which is considered undesirable. However, the control group experienced an average reduction in ApoA and a greater increase in ApoB than the VCO group, though the differences between the groups were not significant. The authors noted that: "Saturated fat is known to have a role in the improvement of HDL-C levels by increasing the activity of lecithin cholesterol acetyltransferase (LCAT)" [14] and stated that: "Dietary interventions that contribute to the increase of HDL concentrations are rare; therefore, our findings were highly significant and unprecedented in this group of patients with chronic coronary disease." The authors further concluded that "a diet rich in extra virgin coconut oil seems to favor the reduction of waist circumference and the increase of HDL-C concentrations, aiding with secondary prevention for CAD patients".

Vijayakumar et al. (2016) [15] was conducted in Kerala, India, where coconut palms are plentiful and 65% of the participants screened for the study were routinely using coconut oil at home. Kerala has a high incidence (about 12%) of coronary heart disease which is multifactorial but often blamed on the use CNO as a staple. This is the largest and longest of the 26 studies reviewed in this analysis and addressed the real-world concern of the effects of oils used for cooking on the lipid profile and other cardiac risk factors in people with known coronary artery disease (CAD). At the beginning of the study, there were 200 free-living non-obese participants (93% male) ages 50 to 65, and 190 completed the two-year study. All participants were previously diagnosed with CAD, of which about 80% had a history of revascularization or bypass surgery, and all were taking statins. Half were assigned to each of two test oil groups to use in cooking and received 15% of total calories based on 24-hour recall as either CNO or sunflower oil, each described only as "commercial". This would provide about 33-40 gm daily of the test oil on a 2000-2400 kcal diet. The rest of the household was provided with the same oil as the participant for the duration of the study. Two people in the CNO group died in traffic accidents, and two people in each group had a revascularization procedure during the two years but there were no other serious adverse events or deaths reported. Fasting bloodwork and other studies were performed at baseline, 3 months, 1 year, and 2 years. **See File S2 Table S1 for lipid profile results.** There were no significant differences from baseline values between the CNO and sunflower oils groups for TChol, LDL-C, HDL-C, VLDL, and TG at 3 months, 1 year, and 2 years. Lp(a) trended higher in the sunflower oil group at two years but was not statistically significant. Ultra-sensitive CRP was slightly lower in the CNO than sunflower oil group but was not statistically significant. There were no significant differences in ApoB or ApoA values or in the ApoB/ApoA-1 ratio between the groups at two years, nor in five antioxidant enzymes, or flow-mediated vasodilation testing. There were no significant changes in hemoglobin A1C (HbA1C) or in anthropometric measurements at 2 years. Subjects did not require an increase in statin medication dosage. The authors concluded that, compared to sunflower oil, CNO did not have any effect on lipid-related and other cardiovascular risk factors and events in people receiving standard treatment for CAD.

Chinwong et al. (2017) [16] was conducted as an open label, randomized controlled crossover trial at Chiang Mai University in Thailand to study the effects of VCO on lipoprotein levels and adverse outcomes. The 32 subjects who completed the study were healthy males and females aged 18 to 25 with normal biomedical parameters and received either VCO 15 ml twice daily (total 30 mL) or a 2% solution of carboxymethylcellulose (CMC) for 8 weeks followed by an 8-week washout period and then 8 weeks on the alternate regimen. Fasting biomedical and lipid profile levels were determined before and after each test period. **See File S2 Table S1 for lipid profile results.** The Friedewald calculation was used to determine LDL-C levels. The average TChol, LDL-C, and TG levels decreased from baseline but not significantly; however, HDL-C increased significantly only in the subjects while taking VCO. TChol, LDL-C, and HDL-C all decreased, and TG increased while taking the CMC solution. The differences in lipid parameters between the two regimens was significant only for HDL-C. No adverse clinical effects related to blood pressure, body weight, renal or hepatic function were noted while taking VCO. The authors concluded that: "The effect of VCO should be potentially beneficial for cardiovascular health but further studies are needed among patients with low HDL-C levels that need to increase their HDL-C level."

Harris et al. (2017) [17] took place at University of Colorado, USA and aimed to determine whether consuming organic virgin coconut oil (VCO) would alter lipid-related cardiac risk factors and inflammatory biomarkers compared to organic high-heat high-oleic (80%) safflower oil in postmenopausal women. The subjects were 12 women aged 55 to 63 with BMI indicating normal or overweight but non-obese with an average TChol at entry of 223 mg/dl $\pm$35. This was a crossover study in which the women were randomly assigned to take 30 ml of VCO or safflower oil for 4 weeks, followed

by a washout period on their usual diet for 4 weeks, then a four-week test period with 30 ml of the other oil. The women were advised to otherwise continue their usual activity level and diet, which averaged about 1700 kcal daily with 39% total calories as fat. There was no appreciable change in body weight for either oil test period; however, there was a small increase in lean body mass in during the VCO test period despite consuming an average of 318 more kilocalories daily compared to the safflower oil test period during which there was a slight decrease in lean body mass, neither change reaching significance. Fasting bloodwork was collected at the beginning and end of each test period. **See See File S2 Table S1 for lipid profile results.** Following the four-week VCO test period, there were significant increases from baseline in TChol, LDL-C, HDL-C, and a non-significant decrease in TG. For safflower oil, there were non-significant decreases in TChol, LDL-C, HDL-C, and increase in TG. TC/HDL ratio did not change for either oil. One individual dropped out due to a reaction (scratchy throat and not feeling well) when taking VCO. Individual inflammatory biomarkers were highly variable after taking both oils with some increasing and others decreasing, except that IL-1ß, a marker of neurodegeneration, decreased in all participants by the end of the VCO test period. The authors noted that, apart from IL-1ß, if an inflammatory biomarker increased or decreased following VCO consumption, the opposite happened with safflower oil in each individual.

Khaw et al. (2018) [18] was conducted in the UK in 91 free-living volunteers recruited for this "real world" study by the British Broadcasting Corporation to compare lipid-related and anthropometric effects of consuming "extra virgin" coconut oil [which does not differ from VCO], butter, or extra virgin olive oil. The subjects were adults (2/3 female) aged 50 to 70 who were generally healthy, non-obese, and not taking lipid-lowering agents. The subjects were randomly assigned to consume 50 ml daily of one of the three oils for four weeks without other dietary instruction except that they could choose to add the oil to the diet or substitute the oil for other foods. The study was completed by 28 people consuming VCO, 33 consuming butter, and 30 consuming olive oil. Compliance was estimated at 75%. Baseline data was reported separately for the 3 groups, including averages for TChol ranging from 228 to 230 mg/dl (SD $\pm$35 to 39) and LDL-C ranging from 135 to 145 mg/dl (SD $\pm$35 to 39). At baseline, the average energy intake was 1967 and 2270 kcal daily for the three groups with 36 to 37% of total calories as fat. Fasting bloodwork was collected at the beginning and end of the four-week test period. Diet analysis revealed that most subjects appeared to add the oil to their diet rather than substituting it for other foods; however, there were no significant changes in body weight, BMI, or waist circumference by the end of the study. **See File S2 Table S1 for lipid profile results.** In the group taking VCO, there were average increases in TChol, HDL-C, and TG, but lower LDL-C, and VCO had the greatest effect of the three fats on reducing the TChol/HDL ratio. For the group taking butter, TChol, LDL-C, HDL-C all increased but TG was unchanged. For the olive oil group, TChol and HDL-C increased, whereas TG decreased, and LDL-C decreased. There were no significant differences in lipid values between olive oil and VCO, which the authors said was unexpected based on general beliefs about CNO and saturated fat. The authors stated further that there is increasing evidence that "different individual fatty acids, such as the odd chain or even chain saturated fatty acids, or short-, medium- and long-chain saturated fatty acids, may have different metabolic pathways and subsequent potential health effects as well as the understanding that diet is more complex than individual nutrients or generic biochemical nutrient groups and that contextual factors such as foods and dietary patterns are important." They also pointed out that the various methods used to process oils could also affect the lipid profile, and that many earlier studies of CNO and olive oil did not specify how the test oils were processed to allow comparison with this study of "extra virgin" coconut and olive oils.

Oliveira-de-Lira et al. (2018) [19] took place in Brazil to study the effects of four different oils on lipid values and anthropometric measurements in a weight-loss study. The subjects were 75 obese women aged 28 to 40 (average 34) with BMI between 30.0 and 39.9 who were not taking lipid lowering or glycemic control medications. The women were divided into four test oil groups, CNO (n=18), safflower oil (n=19), chia oil (n=19), and soybean oil (n=19). The oils were not further specified as to source or type of processing. The women were instructed to follow a balanced diet according to the Brazilian Association for the Study of Obesity and Metabolic Syndrome with 500 fewer kcal daily as determined by the same dietitian for all subjects. The oils were encapsulated by a pharmacist, and the patients were blinded as to which oil they were taking. They were instructed to take two 1-gm capsules before each main meal, totaling just 6 gm daily of the test oil, for the duration of the eight-week study. The women were also advised to walk for 50 minutes at least 4 times weekly. Anthropometric measurements and blood specimens were taken 1 week before and 1 week after the end of the study. All four groups experienced weight loss. The CNO group experienced the largest and soybean oil the smallest improvements in all anthropometric measurements, including weight loss, reduction in BMI, waist circumference, waist-to-height ratio, the percentage of body fat, and percentage of lean body mass. In the CHO group, 17 of the 18 women experienced >5% weight loss compared to just 9 of 19 in the soybean oil group. Four of the women in the coconut oil group lost >10% of their starting weight, compared to just one in the safflower oil group and none in the chia or soybean

oil groups. All four groups saw reductions in HbA1C and mean estimated glycemia (MEG), with the CNO group showing the largest average reductions. **See File S2 Table S1 for lipid profile results.** All four groups experienced significant reductions in TChol, LDL-C, VLDL, TG, and TChol/HDL ratio and increases in HDL-C, with minimal differences between the coconut, safflower oil, and soybean oil groups, and chia oil showing the greatest improvement in these values which was attributed to the high alpha linolenic acid content compared to the other oils. The authors commented that that the study results supported the lack of evidence for negative effects of CNO on cardiovascular health and added to the evidence for positive effects of CNO on the glycemic profile, which were attributed to specific polyphenols found in CNO with anti-diabetic insulin-sensitizing effects. This was a multi-interventional study and the combination of the relatively small amounts of oils with a successful weight-loss diet and increased activity likely explained the positive results for all test oil groups.

Maki et al. (2018) [20] was conducted as a "preliminary investigation" at two sites in Chicago, IL and Boca Raton, FL USA to study effects of CNO versus corn oil on the lipid profile and other measurements. The subjects were adults ages 43 to 47, half male and half female, with BMI 18.5 to 34.9, and baseline LDL-C between 105 and 142 mg/dL with no known atherosclerotic heart disease and not taking lipid lowering or glycemic control agents for at least four weeks. One person dropped out due to marked weight gain during the corn oil test period, and 24 people completed the study. The randomized crossover design began with a four-week period consuming either corn or CNO, a three-week washout period, then a four-week period consuming the other oil. The subjects were instructed to eat four muffins or rolls each containing 1 tablespoon of the test oil totaling 54 gm daily of the test oil and were advised on how to substitute the muffins and rolls for other foods in their habitual diet to maintain their weight. The types and sources of oils used were not reported. The muffins or rolls each contained 225 to 260 kcal with 20 to 27 gm carbohydrate and 2.2 to 4.9 gm protein. It appears that the same baseline blood lipid profile at the beginning of the entire study was used to calculate results for both test periods rather than drawing a new baseline at the beginning of the second four-week test period, which might have affected the reported results. The subjects also completed intravenous glucose tolerance tests at the beginning of the first test period and the end of each of the two test periods which showed no significant differences in results. **See File S2 Table S1 for lipid profile results.** The corn oil test period resulted in small decreases in TChol, LDL-C, TG, and higher HDL-C, whereas the CNO test period resulted in small average increases in TChol, LDL-C, HDL-C, and TG. The only significant difference between the two oils was in non-HDL-C, favoring corn oil. The authors noted that 4 of the 23 subjects in the CNO test and 10 of 23 in the corn oil test period had >5% reduction in LDL-C. Changes in weight and high-sensitivity CRP were not significant during either test period among the people who completed the entire study. The authors noted that the cholesterol lowering effect of corn oil was less than expected.

Korrapati et al. (2019) [21] studied 9 men aged 35 to 38 who were employees of the National Institute of Nutrition in Hyderabad, India with BMI <24.9 and fasting TChol <200 mg/dl. The men were not taking lipid-lowering agents or dietary supplements and were willing to consume a lacto-vegan diet. In this crossover study, all meals and one snack were consumed on the Institute's metabolic unit. For the first 8 weeks, the men received 35 gm CNO daily in locally preferred foods prepared for them totaling about 2600 kcal daily with 60 gm total fat (20% of total calories). There was then a 6-week washout period consuming their habitual diet followed by a second test period of 8 weeks in which they received 35 gm peanut oil daily, again with all meals prepared and similar total calories and macronutrient ratios. The type of processing and oil sources were not reported. Anthropometric measurements and fasting blood sampling were performed at baseline and at the end of each test oil period. There were no significant changes in weight or BMI during either test oil period; however, at the end of the CNO test period, there was a higher percentage of fat free mass, decreased truncal fat, and increased mid-arm circumference, which did not occur during the peanut oil test period. **See File S2 Table S1 for lipid profile results.** At the end of the CNO test period, there was no change in TChol, lower LDL-C, higher HDL-C, higher TG, and lower TChol/HDL ratio, whereas at the end of the peanut oil test period, TChol and LDL-C were increased, and HDL-C, TG, and TC/HDL-C were lower. Lp(a), and ApoA-1 were decreased compared to the entry values at the end of each test oil period, and ApoB was decreased at the end of the CNO test period, but none of those results reached significance. At the end of the CNO test period, the fasting insulin level was decreased but without a change in fasting glucose, which the authors speculated was due to improved insulin sensitivity, along with a decrease in adipose tissue insulin resistance, but these changes did not occur with peanut oil. Extensive measurements of various inflammatory markers and free fatty acid levels were also reported and discussed. The authors concluded that CNO, but not peanut oil, improved the lipid profile and insulin sensitivity.

Vogel et al. (2020) [22] took place at the Federal University of Rio de Janeiro, Brazil and recruited 29 obese men aged 20 to 59 with BMI between 30 and 34.99 kg/m^2 who were otherwise healthy and not taking any medication. This was a parallel randomized controlled study of the effects of "extra" VCO [which is not different than VCO] on weight loss

and metabolic parameters. The men were randomized to take either 12 mL daily at dinner of VCO (n=15) or soybean oil (n=14), which were provided, for 45 days as part of an isocaloric balanced dietary plan based on the Dietary Reference Intakes (2002)[23]. A 3-day dietary record was taken at baseline along with body measurements and fasting blood samples including blood glucose, insulin, and lipid profile levels at baseline and at the end of the study. The Friedewald calculation was used to determine LDL-C. There were no differences in BMI or other body measurements between the groups after the test oil periods. The VCO group had larger decreases from baseline than the soybean oil group for blood glucose, insulin, insulin resistance (HOMA-IR), and a larger increase in insulin sensitivity (QUICKI) compared to the soybean oil group, but the differences were not significant between the groups. **See File S2 Table S1 for lipid profile results.** Both groups had lower TChol and TG levels which were not significantly different. HDL-C increased significantly in the VCO group and decreased in the soybean oil group, and the TChol/HDL-C ratio decreased for the VCO group compared to an increase in the soybean oil group. The decrease in LDL-C for VCO was double the decrease in LDL-C for the soybean oil group but was not statistically significant. The authors concluded that: "Coconut oil incorporated into an isocaloric balanced diet appears to have positives effects on HDL-cholesterol and TC/HDL-cholesterol ratio in men with obesity."

Nikooei et al. (2021) [24] was a randomized controlled clinical trial in 44 men (n=22) and women (n=22) aged 20 to 50 recruited from a screening program at the Tehran Heart Center in Tehran, Iran and were diagnosed with metabolic syndrome (but not diabetes) according to the National Cholesterol Education Program's Adult Treatment Panel III. The subjects were randomly assigned to either use 30 mL daily of VCO (n=24) in place of their usual dietary oils or to continue their usual diet (n=24) for four weeks. The people in the usual diet control group were mostly consuming corn and sunflower oils. The VCO was to be used for low-heat cooking and salad dressing or mixed into milk, coffee, tea, or other foods. The VCO was noted to be extracted from fresh, mature, organically grown coconuts by cold press. Anthropometric measurements, blood pressure, and fasting blood samples for blood glucose and the lipid profile were taken at baseline and at the end of the 4-week study. The lipid profile, including LDL-C, was measured using a commercial enzymatic colorimetric method kit from Pars Azmoon, Inc. There were no significant changes in body measurements or blood pressure between the groups at baseline or at the end of the study. Fasting blood glucose increased significantly in the control group but did not change in the VCO group and was significantly different between the groups at the end of the study. **See File S2 Table S1 for lipid profile results.** TChol, LDL-C, and HDL-C increased significantly for the VCO group compared to the control group and there was no difference in the LDL-C/HDL-C ratio between the two groups. TG levels decreased and were significantly lower in the VCO group compared to the control group. The authors concluded that: "The study findings indicated that consuming 30 ml VCO, as part of the daily fat intake, had some favorable effects on metabolic syndrome components by increasing the values of HDL-C while decreasing that of TG and fasting blood sugar."

Fernando et al. (2023) [25] was a double-blind randomized placebo-controlled trial conducted in 120 men (n=42) and women (n=78) aged ≥ 65 diagnosed with probable mild-to-moderate Alzheimer's who were recruited from the Psychiatry Clinics of North Colombo Teaching Hospital, Ragam and Lanka Alzheimer's Foundation in Sri Lanka. The subjects were randomly assigned to take either 15 mL twice daily in the morning and evening with or before a meal (total 30 mL daily) of VCO (n=60) or canola oil (n=60) for 24 weeks administered with the help of their caregivers. The Mini-Mental Status Exam (MMSE) 30-point test and a clock test were administered at baseline and at the end of the study. A 5-day diet diary and 24-hour recall were collected at baseline and fasting blood samples for the lipid profile were collected at baseline, mid-point, and post intervention, though only the baseline and final values were reported. Having at least one ApoE4 allele is an important genetic risk factor for Alzheimer's disease. The ApoE status of the subjects was determined, and at least one ApoE4 allele was present in 49% of the VCO group and 66% of the canola oil group. **See File S2 Table S1 for lipid profile results.** TChol and LDL-C decreased significantly in the VCO and canola oil group, but the decreases were larger for the VCO group and largest for the subgroup of people with the ApoE4 gene in the VCO group. The VCO group had a larger increase in HDL-C than the canola group, and the increase in HDL-C was the same for the ApoE4 positive and negative subgroups. The TChol/HDL-C ratio decreased in both groups but more so for the VCO group, and the reduction was the same for the ApoE4 positive and negative VCO subgroups. TG increased non-significantly in the VCO group and decreased in the canola oil group. The differences in all the lipid profile values were not statistically significant between the VCO and canola oil groups. Regarding the cognitive testing, The ApoE4 VCO subgroup had improved scores that were significantly higher for the ApoE4 positive subgroup by 2.2 of 30 points than for the ApoE4 negative subgroup. The canola oil group did not have a significant change in the MMSE score, and the clock scores were not significantly different for either group. The authors concluded that: "An improvement in the MMSE score was noted in the ApoE4 carriers but its interpretation is unclear. Supplementation with VCO and canola oil did not compromise lipid parameters and is thus safe for consumption."

Jeyakumar et al. (2023) [26] was a crossover observational study conducted at the ICMR-National Institute of Nutrition, Telangana, India to assess the effects of VCO on indicators of cardiovascular health in non-obese volunteers. The subjects were 22 healthy non-obese (BMI <24.9 kg/m^2), non-smoking men aged 28 to 50 who were employees of the Nutrition Institute with fasting TChol levels <200 mg/dL and were not taking lipid-lowering medications. The men were instructed to continue their usual activity and to avoid alcohol consumption during the study. The men all received 35 gm daily of VCO in provided meals at breakfast, lunch, and dinner for 8 weeks, followed by a 6-week washout period on their usual diet, and then received 35mL daily of peanut oil in the three provided meals for 8 weeks. Anthropometric measurements and fasting blood samples, including the lipid profile, glucose, insulin, fatty acids, plasma C-peptide and fibrinogen, as well as ApoA-I, ApoB, and Lp(a) were taken before and at the end of each 8-week test oil period. LDL-C was determined using the Friedewald calculation. Meals and additional provided snacks were supervised by a dietitian beginning 7 days before the first test oil period and throughout the study. The diet was lactovegetarian using traditional south Indian recipes and food items. The oils were added unheated to prepared foods or minimally heated for cooking. **See File S2 Table S1 for lipid profile results.** TChol and LDL-C increased from baseline values at the end of both test oil periods; however, it should be noted that the differences between the test oil periods were not significant, and TChol levels remained under 200 mg/dL and LDL-C under 130 mg/dL following both test oil periods. HDL-C did not change significantly during the VCO test period but decreased for the peanut oil test period showing a significant difference between the test oil periods. ApoB, Lp(a), and TG levels were not altered in either test oil period. However, the ratios of TChol/HDL-C and LDL-C/HDL-C increased and ApoA-I decreased for both groups but the differences between the groups were not significant. Anthropometric measurements did not change from baseline during either test oil period. There were no significant differences between the test oil periods for fasting glucose and insulin, C-peptide, and three of four indicators of insulin resistance. There were some differences between the test oil periods in adiponectin, adipocytokines, and other inflammatory markers, but C-reactive protein levels were not significantly different. The authors concluded that: "Overall, the consumption of VCO in a balanced diet displayed neutral effects on most of the parameters related to cardiovascular risk and metabolism. However, the rise in plasma total cholesterol and LDL-C as seen here must be tested in larger samples over longer periods than in the current study."

Setyawati et al. (2023) [27] was conducted at the teaching hospital of Universitas Hasanuddin, Makassar, Indonesia and aimed to study the effects of VCO on the lipid profile and the ankle-brachial index (ABI) of people with diabetes mellitus (DM). A total of 136 subjects (53.2% male) aged ≥20 (most 40 to 70 years) completed the study. All subjects had diagnosed DM, dyslipidemia (not defined, but baseline LDL-C was >155 mg/dL on average for both groups) but were not taking lipid-lowering drugs, with average BMI of 26-26.7 kg/m^2), and were not consuming CNO prior to the study. The subjects were asked to continue their lifestyle as usual and were divided into two groups, one receiving VCO (n=68) based on body weight in the amount of 1.2 mL/kg daily divided into three servings and incorporated into meals. This would equate to 72 mL daily for a 60 kg person. The VCO group received half the amount of oil for the first three days and the full amount thereafter, for a total of 30 days. The control group (n=68) was instructed to continue their usual diet and lifestyle. Anthropometric measurements, blood pressures to calculate ABI, and fasting blood samples for lipid profiles were taken at baseline on day 1 and at the end of the study. The Friedewald calculation was used to determine LDL-C values. The right and left ABIs are calculated for each side of the body by taking blood pressures using the same manual cuff in the arms (brachial) and ankles (dorsalis pedis and posterior tibial) in the supine position with limbs straight after resting for 10 minutes. A low ABI indicates that there is reduced blood flow to the ankles suggesting peripheral artery disease, arterial calcification or blockage, whereas a high ABI suggests stiff, non-compressible arteries which can occur in diabetes and chronic kidney disease. **See File S2 Table S1 for lipid profile results.** The VCO group experienced substantial but non-significant decreases in TChol and TG, a significant decrease in LDL-C, and a significant increase in HDL-C, and a slightly improved ABI that was not significant. The usual diet control group had non-significant increases in TChol and TG, a moderate but insignificant decrease in HDL-C, a small decrease in LDL-C, and slight non-significant worsening of ABI. The VCO group consumed less energy by 414 kcal daily than at baseline with a reduction in BMI from 26.0 to 24.2, whereas the control group increased energy intake by 26 kcal daily and had a small average increase in BMI. The authors suggested that: "VCO evidently had anti-hyperlipidaemia activities", and that the lipid profile and ABI trends "are sufficient reasons to claim that VCO exerts a protective effect on DM management in addition to preventing cardiovascular diseases as a DM complication."

Swarnamali et al. (2024) [28] conducted a sequential crossover study at the University of Colombo, Sri Lanka to study the effects on lipids and other metabolic parameters of glycemic control and hepatic function while consuming copra CNO and palm olein, which are two major oils consumed in Sri Lanka. Thirty-seven subjects completed the entire study, which consisted of 8 weeks consuming palm olein, a 16-week washout period on their usual diet (which was longer

than originally planned due to the COVID pandemic), and then 8 weeks consuming CNO. The diets were designed to contain the individual's usual daily calorie intake with 19% total fat and half of the fat as the test oil with the remaining fat from other dietary components. The subjects and their families received palm olein oil for the first test period and CNO for the last test period to incorporate into their food items and cooking for all meals and snacks, and the subjects were asked to record the volume of oil used, which averaged about 17 gm daily. Anthropometric measurements and blood samples were taken at the beginning and end of each of the two test oil periods. LDL-C levels were determined both by calculation and by direct measurement, which is known to be more accurate, and results from direct measurement were used for our analysis. **See File S2 Table S1 for lipid profile results.** For the palm olein test period, TChol, LDL-C, and TG decreased but was not significant for LDL-C, and HDL-C increased slightly. For the CNO test period, TChol and TG increased, LDL-C decreased slightly, and HDL-C increased slightly, all non-significantly. The TC/HDL-C ratio decreased significantly during the palm olein test period but increased non-significantly during the CNO test period. The values for hepatic function and plasma glucose changed slightly but not significantly during each test period. The authors concluded there were no significant differences between CNO and Palm olein for direct LDL-C, HDL-C, TG, VLDL-C, ALT, and AST, but that: "Palm olein demonstrated more favorable effects on certain cardiovascular disease risk-related parameters compared to coconut oil."

Teng et al. (2024) [29] was conducted at the dining hall of the Malaysian Palm Oil Board and at two sites at Universiti Putra Malaysia and aimed to study the relative effects of red palm olein oil (RPOO), "extra" virgin coconut oil [which is the same as VCO], and extra virgin olive oil as a control on cardiometabolic risk markers in people with central obesity. The 143 subjects completing the study were aged 25 to 45 with waist circumference $\geq$90 cm for men and $\geq$80 cm for women and were excluded if they had diagnosed diabetes or other chronic disease, a TChol of > 249 mg/dL, or TG > 399 mg/dL. The study used a parallel design, and the subjects were randomly assigned to consume either RPOO (n=45), VCO (n=48), or EVOO (n=50) for 12 weeks in meals that were provided five days per week but prepared using the oil at home on the weekends. The amount of oil to consume was calculated as 20% of total calories in diets that were 27-30% fat and consisting of Southeast Asian and Western cuisines. The omega-6 PUFA content was adjusted to provide 10% of total calories by adding safflower oil to the VCO diet and additional palm olein to the RPOO diet. The diets averaged 2400 kcal daily and therefore, the average test oil intake was about 53 gm daily. Anthropometric measurements, fasting blood, and urine samples were taken at baseline and at the end of the study, and body weight was monitored every two weeks along with records of test oil intake to assess compliance. The Friedewald calculation was used to determine LDL-C. **See File S2 Table S1 for lipid profile results.** For the VCO group, TChol, LDL-C, HDL-C, and TG all decreased. For the RPOO and EVOO groups, TChol, LDL-C, and HDL-C all decreased, and TG increased. The TChol/HDL-C ratio was essentially unchanged for all three test oil groups. Lipid particle studies, apolipoproteins, plasma fatty acids, vitamin. and mineral levels, and inflammatory markers were also studied. High sensitivity C-reactive protein (hsCRP) increased for the RPOO group and decreased for both the VCO and EVOO groups and the differences between the groups for hs-CRP and other inflammatory markers as well as for apolipoprotein levels, bone density, and fat mass were not significant. The authors concluded that: "The cardiometabolic biomarkers, lipid profile and bone mineral density of individuals with central obesity were statistically similar across the three oils."

References:

1. Reiser, R., Probstfield, J.L., Silvers, A., Scott, L.W., Shorney, M.L., Wood, R.D., O'Brien, B.C., Gotto, A.M., Jr., Insull, W., Jr. Plasma lipid and lipoprotein response of humans to beef fat, coconut oil and safflower oil. *Am J Clin Nutr*, 1985, 42, 190–197.
2. Mendis, S., Kumarasunderam, R. (1990). The effect of daily consumption of coconut fat and soya-bean fat on plasma lipids and lipoproteins of young normolipidaemic men. *Br J Nutr*, 63, 541-552.
3. Heber, D., Ashley, J. M., Solares, M. E., Wang, H. J., & Alfin-Slater, R. B. The effects of a palm-oil enriched diet on plasma lipids and lipoproteins in healthy young men. *Nutr Res*, 1992, 12, S53-59.
4. Cox, C., Mann, J., Sutherland, W., Chisholm, A., & Skeaff, M. Effects of coconut oil, butter, and safflower oil on lipids and lipoproteins in persons with moderately elevated cholesterol levels. *J Lipid Res* 1995, 36(8), 1787-1795.
5. McKenney, J.M., Proctor, J.D., Wright, J.T., Jr., Kolinski, R.J., Elswick, R.K., Jr., Coaker, J.S. The effect of supplemental dietary fat on plasma cholesterol levels in lovastatin-treated hypercholesterolemic patients. *Pharmacotherapy*, 1995, 15, 565–572.
6. Schwab, U.S., Niskanen, L.K., Maliranta, H.M., Savolainen, M.J., Kesäniemi, Y.A., Uusitupa, M.I. Lauric and palmitic acid-enriched diets have minimal impact on serum lipid and lipoprotein concentrations and glucose metabolism in healthy young women. *J Nutr*, 1995,125(3), 466-73.

7. Lu, Z., Hendrich, S., Shen, N., White, P.J., Cook, L.R. Low linolenate and commercial soybean oils diminish serum HDL cholesterol in young free-living adult females. *J Am Coll Nutr*, 1997,16, 562–569.
8. Cox, C., Sutherland, W., Mann, J., de Jong, S., Chisholm, A., & Skeaff, M. Effects of dietary coconut oil, butter, and safflower oil on plasma lipids, lipoproteins, and lathosterol levels. *Eur J Clin Nutr*, 1998, 52(9), 650-654.
9. Assunção, M.L., Ferreira, H.S., dos Santos, A.F., Cabral, C.R. Jr, & Florêncio, T.M. Effects of dietary coconut oil on the biochemical and anthropometric profiles of women presenting abdominal obesity. *Lipids*, 2009, 44, 593–601.
10. Voon, P.T., Ng, T.K., Lee, V.K., Nesaretnam, K. Diets high in palmitic acid (16:0), lauric and myristic acids (12:0 + 14:0), or oleic acid (18:1) do not alter postprandial or fasting plasma homocysteine and inflammatory markers in healthy Malaysian adults. *Am J Clin Nutr*, 2011, 94, 1451-1457.
11. Cardoso, D.A., Moreira, A.S., de Oliveira, G.M., Raggio Luiz, R., & Rosa, G. A coconut extra virgin oil-rich diet increases HDL cholesterol and decreases waist circumference and body mass in coronary artery disease patients. *Nutricion Hospitalaria*, 2015, 32(5), 2144-52.
12. *Dietary Reference Intakes for Energy, Carbohydrate, Fiber, Fat, Fatty Acids, Cholesterol, Protein, and Amino Acids (Macronutrients).* In: Council NR, editor.: The National Academies Press, Washington, DC, 2005.
13. NCEP. Executive summary of the third report of the National Cholesterol Education Program (NCEP) Expert panel on detection, evaluation, and treatment of high blood cholesterol in adults (Adult Treatment Panel III). *JAMA*, 2001, 285(19), 2486-97.
14. Mensink, R.P., Katan, M.B. Effect of dietary fatty acids on serum lipids and lipoproteins. A meta-analysis of 27 trials. *Arterioscler Thromb*, 1992, 12(8), 911-9.
15. Vijayakumar, M., Vasudevan, D.M., Sundaram, K.R., Krishnan, S., Vaidyanathan, K., Nandakumar, S., Chandrasekhar, R., Mathew, N. A randomized study of coconut oil versus sunflower oil on cardiovascular risk factors in patients with stable coronary heart disease. *Indian Heart J*, 2016, 68, 498–506.
16. Chinwong, S., Chinwong, D., & Mangklabruks, A. Daily consumption of virgin coconut oil increases high-density lipoprotein cholesterol levels in healthy volunteers: A randomized crossover trial. *Evid Based Complement Altern Med* 2017, 7251562. Epub 2017 Dec 14.
17. Harris, M., Hutchins, A., & Fryda, L. The impact of virgin coconut oil and high oleic safflower oil on body composition, lipids, and inflammatory markers in postmenopausal women. *J Med Food* 2017, 20, 345–351.
18. Khaw, K.T., Sharp, S.J., Finikarides, L., Afzal, I., Lentjes, M., Luben, R., Forouhi, N.G. Randomised trial of coconut oil, olive oil or butter on blood lipids and other cardiovascular risk factors in healthy men and women. *BMJ Open*, 2018, 8, e020167.
19. Oliveira-de-Lira, L., Santos, E.M.C., de Souza, R.F., Matos, R.J.B., Silva, M.C.D., Oliveira, L.D.S., Nascimento, T.G.D., Schemly, P., Souza, S.L. Supplementation-dependent effects of vegetable oils with varying fatty acid compositions on anthropometric and biochemical parameters in obese women. *Nutrients*, 2018, 20, E932.
20. Maki, K.C., Hasse, W., Dicklin, M.R., Bell, M., Buggia, M.A., Cassens, M.E., Eren, F. Corn oil lowers plasma cholesterol compared with coconut oil in adults with above-desirable levels of cholesterol in a randomized crossover trial. *J Nutr*, 2018, 148, 1556–1563.
21. Korrapati, D., Jeyakumar, S.M., Putcha, U.K., Mendu, V.R., Ponday, L.R., Acharya, V., Koppala, S.R., Vajreswari, A. Coconut oil consumption improves fat-free mass, plasma HDL-cholesterol and insulin sensitivity in healthy men with normal BMI compared to peanut oil. *Clin Nutr*, 2019, 38, 2889–2899.
22. Vogel, C.É., Crovesy, L., Rosado, E.L., Soares-Mota, M. Effect of coconut oil on weight loss and metabolic parameters in men with obesity: a randomized controlled clinical trial. *Food Funct*, 2020, 11(7), 6588-6594.
23. Trumbo, P., Schlicker S., Yates, A.A., Poos M., Food and Nutrition Board of the Institute of Medicine, The National Academies. Dietary reference intakes for energy, carbohydrate, fiber, fat, fatty acids, cholesterol, protein and amino acids. *J Am Diet Assoc*, 2002, 102(11), 1621-1630.
24. Nikooei, P., Hosseinzadeh-Attar, M.J., Asghari, S., Norouzy, A., Yaseri,M., Vasheghani-Farahani, A. Effects of virgin coconut oil consumption on metabolic syndrome components and asymmetric dimethylarginine: A randomized controlled clinical trial. *Nutr Metab Cardiovasc Dis*, 2021, 31(3), 939-949.
25. Fernando, M. G., Silva, R., Fernando, W. M. A. D. B., de Silva, H. A., Wickremasinghe, A. R., Dissanayake, A. S., Sohrabi, H. R., Martins, R. N., & Williams, S. S. Effect of Virgin Coconut Oil Supplementation on Cognition of Individuals with Mild-to-Moderate Alzheimer's Disease in Sri Lanka (VCO-AD Study): A Randomized Placebo-Controlled Trial. *J Alzheimers Dis*, 2023, 96(3), 1195–1206.
26. Jeyakumar, S. M., Damayanti, K., Rajkumar Ponday, L., Acharya, V., Koppala, S. R., Putcha, U. K., Nagalla, B., & Vajreswari, A. Assessment of virgin coconut oil in a balanced diet on indicators of cardiovascular health in non-obese volunteers: A human metabolic study. *Diabetes Metab Syndr*, 2023, 17(9), 102844.
27. Setyawati, A., Sangkala, M.S., Malasari, S., Jafar, N., Sjattar, E.L., Syahrul, S., Rasyid, H. Virgin coconut oil: a dietary intervention for dyslipidaemia in patients with diabetes mellitus. *Nutrients*, 2023, 15(3), 564.
28. Swarnamali, H., Ranasinghe, P., Jayawardena, R. Changes in serum lipids following consumption of coconut oil and palm olein oil: A sequential feeding crossover clinical trial. *Diabetes Metab Syndr*, 2024, 18(6), 103070.
29. Teng, K.T., Loganathan, R., Chew, B.H., Khang, T.F. Diverse impacts of red palm olein, extra virgin coconut oil and extra virgin olive oil on cardiometabolic risk markers in individuals with central obesity: a randomised trial. *Eur J Nutr*, 2024, 63(4), 1225-1239.

Disclaimer/Publisher's Note: The statements, opinions and data contained in all publications are solely those of the individual author(s) and contributor(s) and not of MDPI and/or the editor(s). MDPI and/or the editor(s) disclaim responsibility for any injury to people or property resulting from any ideas, methods, instructions or products referred to in the content.
