# Supplementary material for: Analysis of 26 Studies of the Impact of Coconut Oil on Lipid Parameters: Beyond Total and LDL Cholesterol
Source: Nutrients. 2025 Jan 30;17(3):514. doi: 10.3390/nu17030514 (PMC11819987; doi:10.3390/nu17030514)
Supplement: Supplementary file 1 [file nutrients-17-00514-s001.zip › File S2_Lipid profile results for 26 studies.pdf]

Analysis of 26 Studies of the Impact of Coconut Oil on Lipid Parameters:
Beyond Total and LDL Cholesterol by Mary T. Newport, M.D. and Fabian M. Dayrit, Ph.D.

File S2: Lipid profile results for twenty-six studies

Table S1. Differences in lipid profiles with standard deviations as reported in 26 studies of people consuming coconut oil by duration of study, including 29 groups totaling 984 lipid profile data sets for 792 distinct individuals. Values for TChol, LDL-C, and TG that did not change or decreased are highlighted in green; values for total cholesterol, LDL-C, and TG that increased are highlighted in yellow; values for HDL-C that increased are shown in blue; values for HDL-C that decreased are shown in gray. Base = baseline value Diff = difference; mos = months; NR=not reported; SD = standard deviations; wks = weeks; # = number

Study/Year	Summary: Lipid Profile Results for 29 Groups in 26 Studies of People Consuming Coconut Oil, by Duration of Study																	
Long Duration 1 to 2 years	Duration	# Subjects	Total Cholesterol (mg/dL)				LDL-Cholesterol (mg/dL)				HDL-Cholesterol (mg/dL)				Triglycerides (mg/dL)			
			Base	Diff	%	SD (±)	Base	Diff	%	SD (±)	Base	Diff	%	SD (±)	Base	Diff	%	SD (±)
Vijayakumar 2016 [1]	1 year	96	149.8	-5.23	-3.5	30.9	90.3	0.73	0.81	20.7	40.8	1.61	3.95	9.5	115.0	-2.96	-2.6	50.2
Vijayakumar 2016 [1]	2 years	96	149.8	-0.61	-0.4	28.6	90.3	0.75	0.83	21.8	40.8	2.42	5.93	10.8	115.0	-5.64	-4.9	47.1
Medium Duration 8 to 24 weeks	Duration	# Subjects	Total Cholesterol (mg/dL)				LDL-Cholesterol (mg/dL)				HDL-Cholesterol (mg/dL)				Triglycerides (mg/dL)			
			Base	Diff	%	SD (±)	Base	Diff	%	SD (±)	Base	Diff	%	SD (±)	Base	Diff	%	SD (±)
Mendis 1990 [2]	8 wks	25	179.4	-1.2	-0.6	15.1	114.1	-4.3	-3.7	14.3	42.5	1.5	3.6	10.4	125.8	2.7	2.1	36.3
Oliveira-de-Lira 2018 [3]	8 wks	18	215.6	-17.6	-8.1	17.6	143.2	-14.9	-10.4	17.7	52.9	2.7	5.0	6.4	130.9	-32.6	-24.9	29.1
Korrapati 2019 [4]	8 wks	9	193.0	0.0	0.0	16.1	125.0	-3.0	-2.4	13.9	46.1	4.8	10.4	3.4	106.0	-7.0	-6.6	24.0
Chinwong 2017 [5]	8 wks	32	190.4	-2.7	-1.4	18.0	116.6	-6.1	-5.2	17.7	60.3	3.9	6.5	NR	67.8	-3.1	-4.6	NR
Jeyakumar 2023 [6]	8 wks	22	172.0	14.0	8.1	5.9	113.0	13.0	11.5	2.2	35.0	-0.1	-0.3	17.9	117.0	7.0	6.0	12.3
Swarnamali 2024 [7]	8 wks	37	201.7	8.1	4.0	36.8	137.2	-2.0	-1.5	24.9	42.6	0.5	1.2	11.5	132.5	1.2	0.9	56.8
Assuncao 2009 [8]	12 wks	20	192.5	5.6	2.9	39.0	112.6	3.9	3.5	36.8	45.5	3.2	7.0	2.4	172.8	6.9	4.0	93.7
Teng 2024 [9]	12 wks	48	206.9	-19.3	-9.3	NR	130.3	-14.3	-11.0	NR	55.3	-5.0	-9.1	NR	105.4	-0.9	-0.8	NR
Cardoso 2015 [10]	3 mos	92	177.4	5.9	3.3	35.4	108.3	4.0	3.7	31.2	37.5	3.1	8.3	7.4	153.8	-2.0	-1.3	70.5
Vijayakumar 2016 [1]	3 mos	96	149.8	1.38	0.9	30.2	90.3	-0.98	-1.1	24.6	40.8	0.02	0.0	10.9	115.0	-3.7	-3.2	24.8
Fernando 2023 [11]	24 wks	43	206.3	-6.7	-3.3	44.7	141.2	-10.4	-7.4	41.5	44.7	2.2	4.9	5.9	104.9	4.3	4.1	42.8
Short Duration 3 to 7 weeks	Duration	# Subjects	Total Cholesterol (mg/dL)				LDL-Cholesterol (mg/dL)				HDL-Cholesterol (mg/dL)				Triglycerides (mg/dL)			
			Base	Diff	%	SD (±)	Base	Diff	%	SD (±)	Base	Diff	%	SD (±)	Base	Diff	%	SD (±)
Heber 1992 [12]	3 wks	9	165.0	30.0	18.2	7.0	104.0	25.0	24.0	8.0	40.0	2.0	5.0	4.0	93.0	17.0	18.3	23.0
Lu 1997 [13]	3 wks	15	162.4	-9.3	-5.7	14.7	90.1	-2.3	-2.6	15.9	53.0	-3.5	-6.6	8.1	93.0	-13.3	-14.3	26.6
Schwab 1995 [14]	4 wks	15	186.8	0.8	0.4	6.2	112.1	-1.9	-1.7	4.6	59.9	-2.3	-3.9	2.7	80.6	-3.5	-4.4	8.0
Cox 1998 [15]	4 wks	37	212.7	-1.2	-0.6	35.2	137.7	8.9	6.5	29	42.5	4.3	10	10.4	156.8	-14.2	-9.0	82.4
Harris 2017 [16]	4 wks	12	219.6	18.2	8.3	24.1	124.0	13.5	10.9	27.2	63.9	6.6	10.3	18.8	117.2	-9.7	-8.3	80.6
Khaw 2018 [17]	4 wks	28	228.2	8.5	3.7	21.3	135.3	-3.5	-2.6	19.0	77.3	10.8	14.0	11.2	78.8	6.2	7.9	51.4
Maki 2018 [18]	4 wks	12	188.0	13.3	7.1	NR	123.0	5.7	4.6	NR	46.0	3.0	6.5	NR	92.5	5.5	5.9	NR
Nikooei 2021 [19]	4 wks	22	206.3	32.6	15.8	53.6	107.6	21.0	19.5	26.3	44.2	8.3	18.8	2.3	216.5	-44.4	-20.5	114.6
Setyawati 2023 [20]	30 days	68	247.7	-21.1	-8.5	8.1	179.6	-38.2	-21.2	7.2	39.9	9.4	23.5	2.3	192.8	-9.2	-4.8	5.0
Reiser 1985 [21]	5 wks	16	158.0	10.0	6.3	3.0	96.0	14.0	14.6	4.1	45.0	1.0	2.2	1.1	80.0	-2.0	-2.5	3.6
Voon 2011 [22]	5 wks	45	182.1	9.3	5.1	26.7	118.3	9.3	7.8	29.0	47.6	5.4	11.4	11.6	85.0	-5.3	-6.2	34.5
Cox 1995, Men [23]	6 wks	13	251.4	3.7	1.5	34.0	166.3	4.7	2.8	33.0	46.4	-1.4	-3.0	6.0	203.7	27.3	13.4	97.0
Cox 1995, Women [23]	6 wks	15	239.8	3.3	1.4	24.0	154.1	1.9	1.3	25.0	69.6	-1.6	-2.3	11.0	124.0	-9.0	-7.3	27.0
McKenney 1995 #1 [24]	6 wks	11	222.3	11.0	4.9	19.0	149.0	6.4	4.3	19.5	49.8	4.1	8.2	15.9	117.1	2.9	2.5	47.7
McKenney 1995 #2 [24]	6 wks	17	214.0	-5.7	-2.7	20.0	138.6	-12.8	-9.2	12.8	50.9	5.2	10.2	12.4	122.3	9.5	7.8	64.8
Vogel 2020 [25]	45 days	15	180.1	-8.6	-4.8	49.4	112.5	-11.5	-10.2	37.2	39.4	3.7	9.3	14.9	141.0	-2.2	-1.6	78.3

References

1. Vijayakumar, M., Vasudevan, D.M., Sundaram, K.R., Krishnan, S., Vaidyanathan, K., Nandakumar, S., Chandrasekhar, R., Mathew, N. A randomized study of coconut oil versus sunflower oil on cardiovascular risk factors in patients with stable coronary heart disease. *Indian Heart J*, 2016, 68, 498–506.
2. Mendis, S., Kumarasunderam, R. (1990). The effect of daily consumption of coconut fat and soya-bean fat on plasma lipids and lipoproteins of young normolipidaemic men. *Br J Nutr*, 63, 541-552
3. Oliveira-de-Lira, L., Santos, E.M.C., de Souza, R.F., Matos, R.J.B., Silva, M.C.D., Oliveira, L.D.S., Nascimento, T.G.D., Schemly, P., Souza, S.L. Supplementation-dependent effects of vegetable oils with varying fatty acid compositions on anthropometric and biochemical parameters in obese women. *Nutrients*, 2018, 20, E932.
4. Korrapati, D., Jeyakumar, S.M., Putcha, U.K., Mendu, V.R., Ponday, L.R., Acharya, V., Koppala, S.R., Vajreswari, A. Coconut oil consumption improves fat-free mass, plasma HDL-cholesterol and insulin sensitivity in healthy men with normal BMI compared to peanut oil. *Clin Nutr*, 2019, 38, 2889–2899.
5. Chinwong, S., Chinwong, D., & Mangklabruks, A. Daily consumption of virgin coconut oil increases high-density lipoprotein cholesterol levels in healthy volunteers: A randomized crossover trial. *Evid Based Complement Altern Med*, 2017, 7251562. Epub 2017 Dec 14.
6. Jeyakumar, S. M., Damayanti, K., Rajkumar Ponday, L., Acharya, V., Koppala, S. R., Putcha, U. K., Nagalla, B., & Vajreswari, A. Assessment of virgin coconut oil in a balanced diet on indicators of cardiovascular health in non-obese volunteers: A human metabolic study. *Diabetes Metab Syndr*, 2023, 17(9), 102844.
7. Swarnamali, H., Ranasinghe, P., Jayawardena, R. Changes in serum lipids following consumption of coconut oil and palm olein oil: A sequential feeding crossover clinical trial. *Diabetes Metab Syndr*, 2024, 18(6), 103070.
8. Assunção, M.L., Ferreira, H.S., dos Santos, A.F., Cabral, C.R. Jr, & Florêncio, T.M. Effects of dietary coconut oil on the biochemical and anthropometric profiles of women presenting abdominal obesity. *Lipids*, 2009, 44, 593–601.
9. Teng, K.T., Loganathan, R., Chew, B.H., Khang, T.F. Diverse impacts of red palm olein, extra virgin coconut oil and extra virgin olive oil on cardiometabolic risk markers in individuals with central obesity: a randomised trial. *Eur J Nutr*, 2024, 63(4), 1225-1239.
10. Cardoso, D.A., Moreira, A.S., de Oliveira, G.M., Raggio Luiz, R., & Rosa, G. A coconut extra virgin oil-rich diet increases HDL cholesterol and decreases waist circumference and body mass in coronary artery disease patients. *Nutricion Hospitalaria*, 2015, 32(5), 2144-52.
11. Fernando, M. G., Silva, R., Fernando, W. M. A. D. B., de Silva, H. A., Wickremasinghe, A. R., Dissanayake, A. S., Sohrabi, H. R., Martins, R. N., & Williams, S. S. Effect of Virgin Coconut Oil Supplementation on Cognition of Individuals with Mild-to-Moderate Alzheimer's Disease in Sri Lanka (VCO-AD Study): A Randomized Placebo-Controlled Trial. *J Alzheimers Dis*, 2023, 96(3), 1195–1206.
12. Heber, D., Ashley, J. M., Solares, M. E., Wang, H. J., & Alfin-Slater, R. B. The effects of a palm-oil enriched diet on plasma lipids and lipoproteins in healthy young men. *Nutr Res*, 1992, 12, S53-59.
13. Lu, Z., Hendrich, S., Shen, N., White, P.J., Cook, L.R. Low linolenate and commercial soybean oils diminish serum HDL cholesterol in young free-living adult females. *J Am Coll Nutr*, 1997,16, 562–569.
14. Schwab, U.S., Niskanen, L.K., Maliranta, H.M., Savolainen, M.J., Kesäniemi, Y.A., Uusitupa, M.I. Lauric and palmitic acid-enriched diets have minimal impact on serum lipid and lipoprotein concentrations and glucose metabolism in healthy young women. *J Nutr*, 1995,125(3), 466-73.
15. Cox, C., Sutherland, W., Mann, J., de Jong, S., Chisholm, A., & Skeaff, M. Effects of dietary coconut oil, butter, and safflower oil on plasma lipids, lipoproteins, and lathosterol levels. *Eur J Clin Nutr*, 1998, 52(9), 650-654.
16. Harris, M., Hutchins, A., & Fryda, L. The impact of virgin coconut oil and high oleic safflower oil on body composition, lipids, and inflammatory markers in postmenopausal women. *J Med Food* 2017, 20, 345–351.

17. Khaw, K.T., Sharp, S.J., Finikarides, L., Afzal, I., Lentjes, M., Luben, R., Forouhi, N.G. Randomised trial of coconut oil, olive oil or butter on blood lipids and other cardiovascular risk factors in healthy men and women. *BMJ Open*, 2018, 8, e020167.

18. Maki, K.C., Hasse, W., Dicklin, M.R., Bell, M., Buggia, M.A., Cassens, M.E., Eren, F. Corn oil lowers plasma cholesterol compared with coconut oil in adults with above-desirable levels of cholesterol in a randomized crossover trial. *J Nutr*, 2018, 148, 1556–1563.

19. Nikooei, P., Hosseinzadeh-Attar, M.J., Asghari, S., Norouzy, A., Yaseri,M., Vasheghani-Farahani, A. Effects of virgin coconut oil consumption on metabolic syndrome components and asymmetric dimethylarginine: A randomized controlled clinical trial. *Nutr Metab Cardiovasc Dis*, 2021, 31(3), 939-949.

20. Setyawati, A., Sangkala, M.S., Malasari, S., Jafar, N., Sjattar, E.L., Syahrul, S., Rasyid, H. Virgin coconut oil: a dietary intervention for dyslipidaemia in patients with diabetes mellitus. *Nutrients*, 2023, 15(3), 564.

21. Reiser, R., Probstfield, J.L., Silvers, A., Scott, L.W., Shorney, M.L., Wood, R.D., O'Brien, B.C., Gotto, A.M., Jr., Insull, W., Jr. Plasma lipid and lipoprotein response of humans to beef fat, coconut oil and safflower oil. *Am J Clin Nutr*, 1985, 42, 190–197.

22. Voon, P.T., Ng, T.K., Lee, V.K., Nesaretnam, K. Diets high in palmitic acid (16:0), lauric and myristic acids (12:0 + 14:0), or oleic acid (18:1) do not alter postprandial or fasting plasma homocysteine and inflammatory markers in healthy Malaysian adults. *Am J Clin Nutr*, 2011, 94, 1451-1457.

23. Cox, C., Mann, J., Sutherland, W., Chisholm, A., & Skeaff, M. Effects of coconut oil, butter, and safflower oil on lipids and lipoproteins in persons with moderately elevated cholesterol levels. *J Lipid Res* 1995, 36(8), 1787-1795.

24. McKenney, J.M., Proctor, J.D., Wright, J.T., Jr., Kolinski, R.J., Elswick, R.K., Jr., Coaker, J.S. The effect of supplemental dietary fat on plasma cholesterol levels in lovastatin-treated hypercholesterolemic patients. *Pharmacotherapy*, 1995, 15, 565–572.

25. Vogel, C.É., Crovesy, L., Rosado, E.L., Soares-Mota, M. Effect of coconut oil on weight loss and metabolic parameters in men with obesity: a randomized controlled clinical trial. *Food Funct*, 2020, 11(7), 6588-6594.

Disclaimer/Publisher's Note: The statements, opinions and data contained in all publications are solely those of the individual author(s) and contributor(s) and not of MDPI and/or the editor(s). MDPI and/or the editor(s) disclaim responsibility for any injury to people or property resulting from any ideas, methods, instructions or products referred to in the content.
